# A Simple and Scalable Strategy for Analysis of Endogenous Protein Dynamics

**DOI:** 10.1038/s41598-020-65832-1

**Published:** 2020-06-02

**Authors:** Marie K. Schwinn, Leta S. Steffen, Kris Zimmerman, Keith V. Wood, Thomas Machleidt

**Affiliations:** 1 Promega Corporation, Madison, Wisconsin 53711 United States; 2 Light Bio, Inc., Madison, Wisconsin 53711 United States

**Keywords:** Biochemistry, Biological techniques, Biotechnology, Cell biology

## Abstract

The ability to analyze protein function in a native context is central to understanding cellular physiology. This study explores whether tagging endogenous proteins with a reporter is a scalable strategy for generating cell models that accurately quantitate protein dynamics. Specifically, it investigates whether CRISPR-mediated integration of the HiBiT luminescent peptide tag can easily be accomplished on a large-scale and whether integrated reporter faithfully represents target biology. For this purpose, a large set of proteins representing diverse structures and functions, some of which are known or potential drug targets, were targeted for tagging with HiBiT in multiple cell lines. Successful insertion was detected for 86% of the targets, as determined by luminescence-based plate assays, blotting, and imaging. In order to determine whether endogenously tagged proteins yield more representative models, cells expressing HiBiT protein fusions either from endogenous loci or plasmids were directly compared in functional assays. In the tested cases, only the edited lines were capable of accurately reproducing the anticipated biology. This study provides evidence that cell lines expressing HiBiT fusions from endogenous loci can be rapidly generated for many different proteins and that these cellular models provide insight into protein function that may be unobtainable using overexpression-based approaches.

## Introduction

Of the ~20,000 protein coding genes within the human genome, fewer than 10% are targets of research and drug discovery programs^1^. One factor that may influence which proteins are studied is simply the availability of technologies or reagents to investigate particular targets. Development of tools that enable analysis of any member of the proteome would strengthen understanding of the function of these understudied proteins, as well as accelerate discovery of therapeutic compounds that modulate their activities. Furthermore, technologies that could be easily applied to large numbers of proteins in parallel would benefit the systematic investigation of larger subsets of proteins representing functional complexes or closely related protein families. Current approaches fall short in providing functional analysis of large proteins sets in a manner that is simple, fast, and compatible with live cell analysis. Thus, the availability of a universal and easily implemented method for the study of endogenous proteins would be of significant value for both the study of understudied proteins, as well as the analysis of protein complexes and families.

Mass spectrometry and antibody-based detection are two principal methods for studying expression, localization, processing, modifications, and interactions of individual proteins. Although these well-established techniques have proven invaluable for protein analysis, both face technical limitations that impede their use in functional proteomics. Specifically, mass spectrometry tends to under-represent low abundance proteins, while antibody-based techniques are restricted by the availability of high quality, specific antibodies^2,3^. Of significance, both require cell lysis which prevents real time analysis and disrupts the spatiotemporal dynamics that underlie basic physiology. An ideal method for functional proteomics should permit live cell experimentation in such a way that is quantitative, sensitive, and scalable.

To circumvent the constraints of mass spectrometry and immunoanalysis, target proteins are often overexpressed as fusions to a reporter. This permits functional and quantitative analysis without the need for specific reagents, complex workflows, or cell lysis. Further, transient or stable overexpression of these recombinant reporter fusions offers the ability to evaluate protein dynamics in real time in a variety of cell lines. However, protein overexpression typically yields cellular protein levels that are markedly different from endogenous. Disruption to the natural stoichiometry of proteins within a cell could contribute to expression artifacts such as aggregation, mis-localization and altered functional responses^4^. Additionally, plasmid-based gene overexpression is often driven by synthetic promoters, thereby prohibiting the study of native transcriptional regulatory mechanisms that control expression of endogenous proteins^5^. These risks are concerning in situations where expression levels directly impact function, as is the case for multiprotein complexes and protein-protein interactions.

The potential for overexpression artifacts and dysregulated transcription could be avoided by directly integrating reporters into endogenous genomic loci. With the development of CRISPR/Cas9 genome editing tools, integration of reporter sequences can now be accomplished with greater speed and ease. We recently demonstrated a method to accurately quantitate endogenous proteins by fusing the luminescent HiBiT peptide onto proteins using CRISPR/Cas9. The small (1.3 kDa) HiBiT peptide complements with high affinity to a larger (18 kDa) subunit evolved from NanoLuc (termed LgBiT). The resulting complex (i.e., reconstituted luciferase enzyme) generates bright luminescence that translates to sensitivity (1 amol), broad dynamic range (four orders of magnitude), and rapid kinetics for real time quantitation^6^. While small tags are desirable because of their presumed minimal impact on endogenous biology, they can also be incorporated into the genome much more rapidly and with higher efficiency than full-length reporter proteins. Furthermore, efficient site-specific HiBiT insertion can be achieved without the need for cloning procedures using Cas9/guide RNA (gRNA) ribonucleoprotein (RNP) complexes and synthetic single-stranded oligonucleotide (ssODN) DNA templates. Upon delivery of these components into the cell, the donor template is likely integrated via single-strand template repair (SSTR)^7^. This method is not unique to HiBiT integration and has been applied to knockin of several small tags, including epitope tags (e.g., FLAG, HA, Myc) and fluorescent complementation peptides (e.g., GFP11, mCherry11)^8,9^. However, detection of epitope tags requires cell lysis or permeabilization steps for antibody based detection which prevents real time analysis of protein dynamics in living cells. While fluorescent complementation allows for live cell detection it is limited by relatively slow fluorophore maturation kinetics as well as poor sensitivity and dynamic range due to cellular autofluorescence^10,11^. As previously shown, the luminescent HiBiT tag combines rapid kinetics with exceptional sensitivity and linear range, making it a versatile tag for the quantitation of both low and high abundance proteins in living cells.

A primary objective of this study was to explore the scalability of CRISPR-mediated HiBiT tagging to enable rapid and parallel generation of cell models for most proteins. Previous endogenous tagging studies have concentrated on a relatively small number of targets restricted to functionally or structurally related classes of proteins. In order to establish the broad and scalable use of endogenous HiBiT tagging, we deliberately targeted a larger set of proteins representing diverse functions, structures, and subcellular localizations in multiple cell lines. While previous endogenous tagging studies do not demonstrate this level of scalability, they also fall short in presenting direct benefits of using reporter knockin cell lines. Therefore, a secondary objective of this study was to show that these edited cells better capture biology than traditional overexpression. For this purpose, two targets that are tightly regulated at both the transcriptional and post-translational levels, FOS and NFKBIA, were selected for comparative analysis^12,13^. Taken together, the results will demonstrate that CRISPR-mediated HiBiT tagging of endogenous proteins is a broadly applicable strategy that enables the study of numerous proteins in the human proteome, and they will show that cell lines edited to express reporter fusions more accurately capture biology than overexpression techniques.

## Results

### Large-scale endogenous HiBiT tagging

We previously described rapid on-target integration of HiBiT by delivering RNP complexes consisting of recombinant Cas9 and synthetic gRNA to cells via electroporation in the presence of ssODN donor DNA^6^. This approach avoids the need for cloning, eliminates the risk of illegitimate recombination of donor DNA plasmid into the host genome, and allows for more efficient and specific on-target editing^14^. However, this initial study was limited to the examination of a small number of proteins associated with a single signaling pathway. For CRISPR-mediated HiBiT tagging to be considered scalable and broadly applicable for studying endogenous proteins, it is essential that successful tagging and detection be demonstrated across a panel of targets that captures the diversity of the proteome. It is also critical that a simple standardized protocol for the genome editing procedure can be established such that tag integration can be performed in parallel for different targets without the need for target-specific optimization.

**Figure 1:**
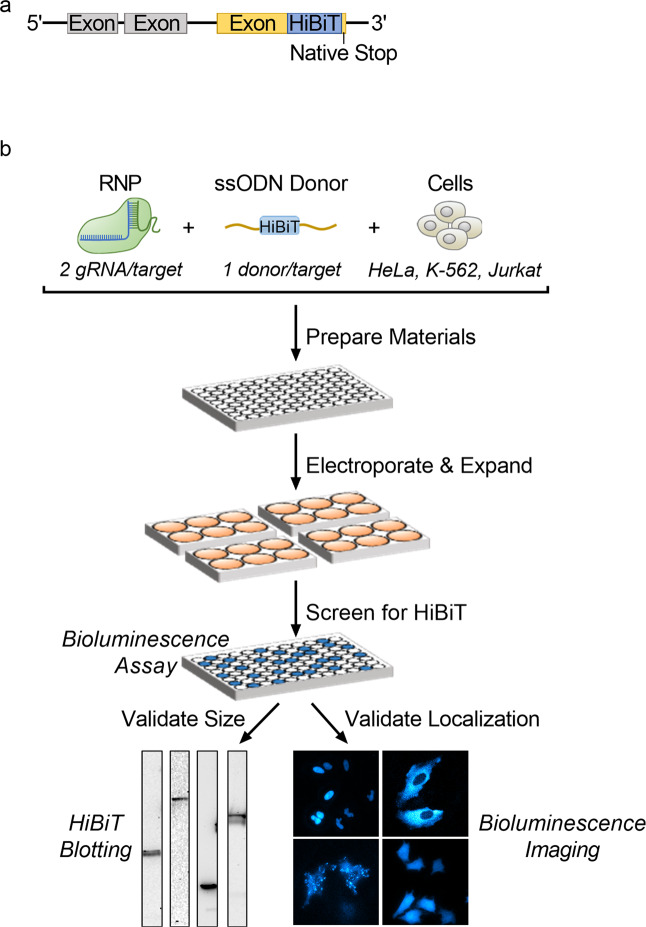
Workflow for large-scale HiBiT endogenous tagging. (**a**) Schematic showing integration of HiBiT sequence immediately upstream of native stop codon to generate C-terminal protein fusion. (**b**) Diagram of editing and validation procedure for large-scale knockin of HiBiT. For each target, two gRNA and one donor were used to edit HeLa, K-562, and Jurkat cells. Editing components were combined in 96-well plates and then delivered into the cells via electroporation. Cells were expanded for 72 h in 6-well plates and then screened for integration using the HiBiT lytic bioluminescence assay. Expression of properly sized proteins and cellular localization were validated using HiBiT blotting and bioluminescence imaging.

Taking these considerations into account, 97 targets were selected which represented diversity in size (11.4 to 350.7 kDa), subcellular localization (nucleus, plasma membrane, cytoplasm, Golgi apparatus, mitochondrion, endoplasmic reticulum, endosomes, and cytoskeleton), physiological function (structural, signaling, apoptosis, metabolism, and epigenetics), and transcript expression levels (Table S1). To maintain simplicity, the carboxy-terminus, immediately upstream of the native stop codon, was selected as integration site for all targets (Fig. 1a). For each gene, two gRNA and one ssODN donor template were tested in parallel in HeLa and K-562 cell lines (Tables S2 and S3). In order for this method to be considered universal, it must also be reasonably effective in cell lines known to be more difficult to edit. For this reason, Jurkat cells, which are generally recognized for being difficult to transfect, were also targeted for editing (Fig. 1b). Since integration is typically higher the closer the double-strand break is to the insertion site, gRNAs directing cuts within 10 nucleotides of the insertion site were selected whenever possible. Furthermore, cut sites within the 3′ untranslated region were preferred to avoid potential NHEJ induced genetic lesions in the coding region of the target protein. All ssODN were designed by placing the HiBiT tag and a valine-serine linker symmetrically between 50 nucleotide homology arms. In situations where the gRNA recognition sequences were not disrupted by HiBiT integration, mutations to the PAM site or seed region of the gRNA were introduced in the donor template to avoid re-cutting. Successful integration of the HiBiT tag was determined 72 h after editing by measuring luminescence in cell lysates in the presence of LgBiT protein in comparison to mock-edited control (cells electroporated with Cas9 and ssODN only). Luminescence detected with this simple screening approach likely originates only from on-target integration. Unlike for full-length reporters, nonspecific signal resulting from off-target integration of HiBiT is extremely unlikely as no repair pathway has been reported that promotes significant integration of single strand templates by NHEJ^7^. In the rare instance of random integration, any off-target expression of HiBiT would require in-frame insertion into a protein encoding gene, as the donor template itself lacks a functional promoter.

**Figure 2:**
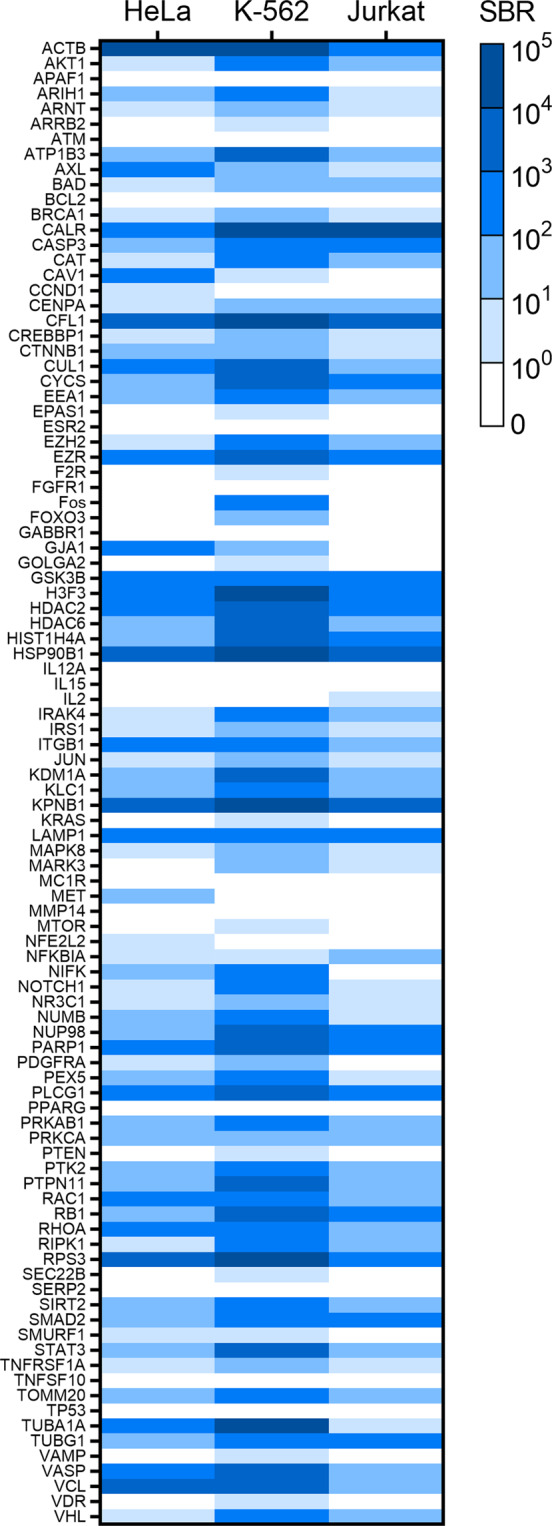
Distribution of luminescence signal across panel of targets. Heat map displaying ranges of SBR for 97 targets in pools of HeLa, K-562, and Jurkat cells. Luminescence measurements were obtained in lytic mode 72 h post-editing and normalized to total cell number. The SBR was calculated as follows: (RLU_edited cells_ – RLU_mock edited cells_)/RLU_mock edited cells_. For HeLa and K-562 cell lines, two different pools were generated for each target using different gRNA. Shown here are the highest SBR of the two pools. For Jurkat cell lines, one pool was generated for each target using the guide that generated the highest SBR in K-562 cells. Colors increase from white to dark blue, where white represents targets with SBR less than one and shades of blue represent targets with SBR greater than or equal to one.

For this approach to be considered scalable, it is important to establish that HiBiT insertion can be achieved without multiple editing attempts or additional optimization. Thus, the results reported here are based on a single round of editing across multiple cellular backgrounds without introducing variations to the protocol. For the purpose of this study, successful integration is defined as detection of luminescence signal that is at least two times that of background (i.e., an absolute signal-to-background ratio, SBR, greater than one). Using this measure, HiBiT tagging was successful for 72% of the targets in HeLa cells, 82% of the targets in K-562 cells, and 65% of the targets in Jurkat cells (Fig. 2, Tables S4–6). To assess reproducibility, two independent editing experiments were performed for 16 targets in HeLa cells. A strong correlation (R^2^ = 0.92) between signal strength obtained in both experiments was observed, suggesting that the editing process is reproducible (Fig. S1). However, variability in targeting efficiency as well as signal strength for a given target was noted across the different cell lines. Given that the editing procedure itself was shown to be reproducible, this likely represents differences in both expression levels as well as integration efficiency between different cell lines. For example, expression of MET and CCND1 transcripts have been reported in HeLa but not K-562 cells which is consistent with the results showing detectable luminescence in the edited HeLa but not K-562 cells. Likewise, F2R transcript expression has been reported for K-562 cells but not HeLa, which is also in agreement with the results of the luminescence assay (Table S7). When the data were combined to account for variabilities in expression between cell lines, 83 of the 97 (86%) protein targets were successfully edited in at least one line. This illustrates that in contrast to overexpression models, the selection of a cell line that expresses the protein target of choice becomes critical. In some situations, the most appropriate line for obtaining disease relevant data may be primary cells which can be challenging to edit due to inefficient uptake of editing components and unfavorable DNA repair mechanisms. To demonstrate the ability to integrate HiBiT and detect fusion expression in primary cells, a round of editing was performed using activated primary T cells isolated from peripheral blood mononuclear cells. Of the 17 genes targeted, 13 (76%) were successfully edited, suggesting that large-scale integration of HiBiT and subsequent detection of protein fusions is principally possible in more challenging primary cells (Fig. S2).

Lack of luminescence may result from a number of issues other than technical failure to insert HiBiT. For instance, genes not expressed in the selected cell lines would fail to generate detectable luminescence. In the case of five of the genes (BCL2, ESR2, IL12A, SERP2, and TNFSF10), no reported expression data were found for either the K-562 or HeLa cells, suggesting lack of signal was due to the absence of gene expression in the chosen lines. For other targets, native protein abundance may be low or absent until pathway stimulation. For example, the protein products of FOS and EPAS1 were undetectable until treatment with PMA or phenanthroline, respectively (Fig. S3a,b). It is also possible that lack of signal may reflect termini sensitivity to tagging. For instance, MMP14 behavior is known to be negatively influenced by disruption of the C terminus^15^. Consistent with this, luminescence was only measurable for MMP14 (in U2-OS cells known to express this gene) when HiBiT was inserted as an internal tag (Fig. S4). Lastly, it is possible that a low percentage of targets were not detected due to editing difficulties resulting from chromatin inaccessibility or suboptimal gRNA design^16^.

### Validation of cell pools

**Figure 3:**
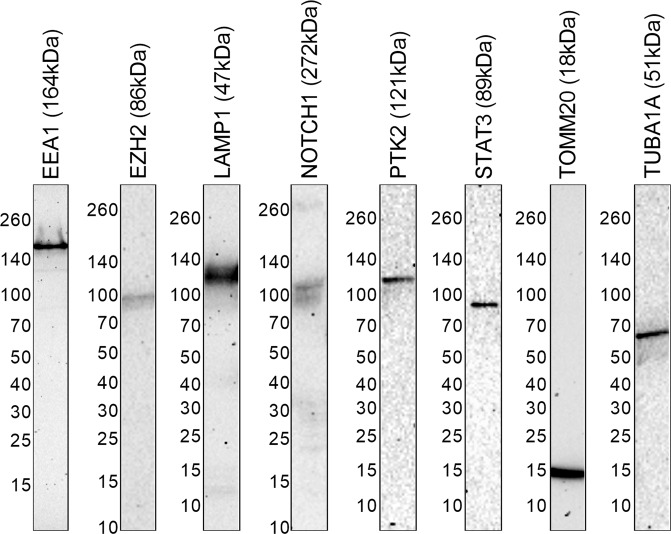
HiBiT blotting to validate size of HiBiT fusions in edited K-562 pools. Represented are sections of different blots for the indicated protein fusion with expected apparent molecular weights in parentheses. Values to the left of each blot represent the migration of the corresponding size standard. Blots were generated by running lysates from edited K-562 pools on SDS-PAGE gels, transferring proteins to nitrocellulose membranes, and detecting luminescence after incubation with LgBiT protein and Nano-Glo Luciferase Assay Substrate. Linear contrast adjustments were made using Fiji in order to make each protein detectable. Uncropped blots are available in Fig. S6a–j.

After each cell pool had been screened for luminescence, additional validation was performed to confirm the expression of HiBiT tagged protein by size and cellular localization pattern. Although validation of protein expression and localization is usually performed using Western blotting and immunofluorescence microscopy, these techniques require target specific antibodies. Implementation on a large-scale or for understudied proteins would be particularly challenging due to the limited availability of quality antibodies, as well as reagent cost and workflow optimization required for each target. In the case of Western blotting, the small size of the HiBiT tag with added linker (1.5 kDa) also makes it challenging to distinguish between fusion and native protein on a blot. We previously demonstrated that HiBiT tag enables protein analysis by blotting and bioluminescence imaging without the need for target-specific antibodies^6^. HiBiT blotting is accomplished by separating lysates on a SDS-PAGE gel, transferring the proteins to a nitrocellulose membrane, incubating the membrane with purified LgBiT and substrate, and then detecting luminescent bands with a gel imager. HiBiT blotting was performed to visualize protein fusion size in each of the 80 edited K-562 cell pools that were initially identified as hits in the luminescence plate assay. Out of these pools, 64 (80%) of the fusion proteins were detectable by blotting and appeared with expected sizes and banding patterns (Figs. 3 and S5a–j, S6a–j). For some targets, band shifts or multiple bands, consistent with reported processing or modification events, were observed which suggests that HiBiT does not interfere with protein biology. For example, the predicted molecular weight of LAMP1-HiBiT is 47 kDa, but as a result of glycosylation, it migrates with an apparent molecular weight near 130 kDa, as observed with HiBiT blotting^17^. Another target, NOTCH1 has a molecular weight of 272 kDa but is proteolytically processed to release a 91 kDa cytosolic fragment^18^. Both intact and cleaved NOTCH1-HiBiT were observed on the HiBiT blot. In general, HiBiT blotting provided fast validation of protein expression and expected processing or modifications without the need for specific antibodies or extensive workflow optimization. It was noted that 15 of the fusions were undetectable by HiBiT blotting, with the majority of these targets (11 of 15) exhibiting low signal strength (SBR < 10) in the luminescence assay. This likely reflects reduced sensitivity of the blotting technique relative to the plate assay. While suitable for detection of most targets, HiBiT blotting is a relatively new technique and may require refinement to approach the level of sensitivity achieved by the plate assay. To improve detection of low abundance proteins, sample processing to increase the amount of protein fusion loaded onto a gel may be necessitated. Fractionation of lysates or selective enrichment of HiBiT using LgBiT immobilized to resin could be incorporated into the workflow to increase the amount of fusion protein in the sample, as demonstrated previously^6^.

**Figure 4:**
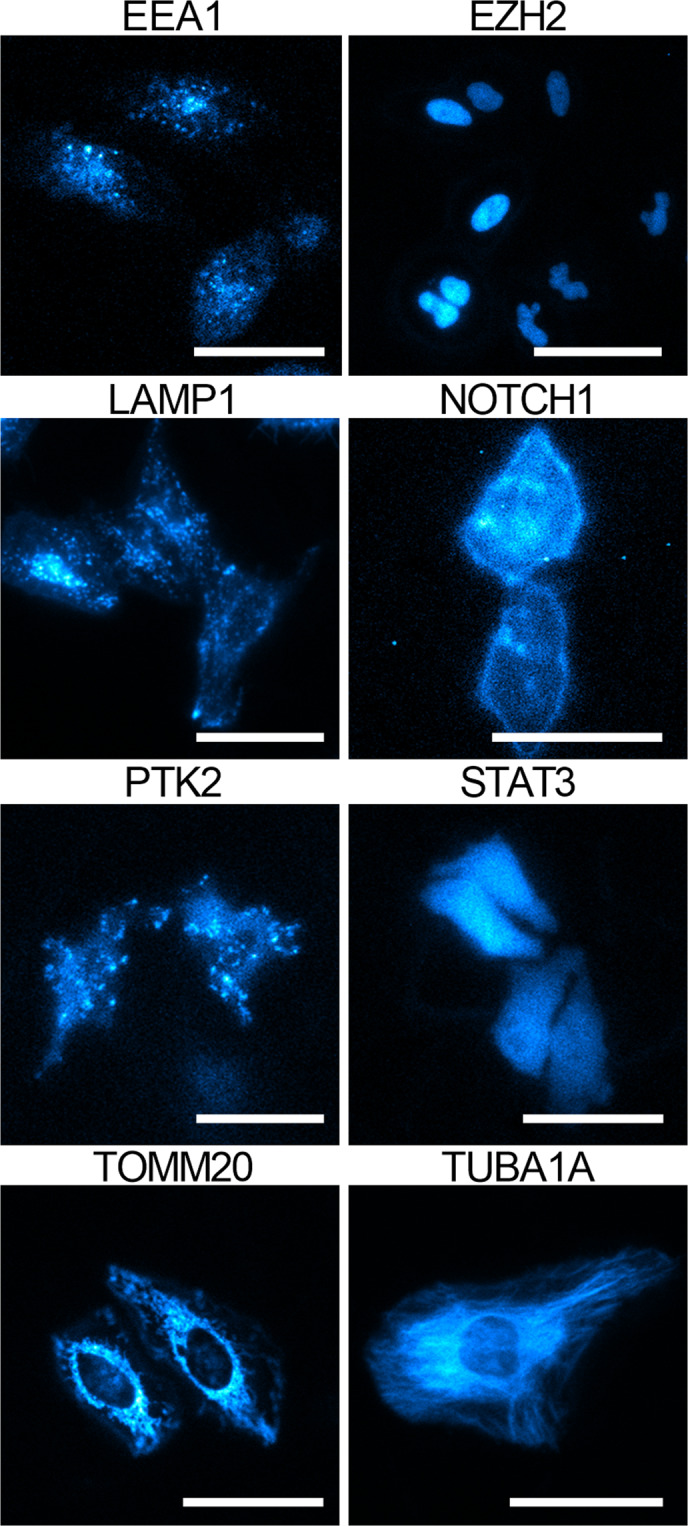
Bioluminescence imaging to validate subcellular localization of HiBiT fusion in edited HeLa pools. Panels show pseudo-colored bioluminescence images of HiBiT fusion in edited HeLa cell pools. Images were acquired using either a 60×, NA 1.4 objective (EEA1, EZH2, LAMP1, PTK2, STAT3, TOMM20, and TUBA1A) or 100×, NA 1.4 objective (NOTCH1) 24 h after plating in the presence of BacMam LgBiT (5% v/v). EM gain and exposure times for each image are found in Table S8. Scale bar = 20 µm.

Another critical aspect of validating edited cell lines is to confirm that the tag does not interfere with proper localization within the cell. Standard imaging of epitope tags, such as FLAG, involves fixation, permeabilization, antibody incubation, and wash steps that can be detrimental to the target protein and can also introduce artifacts^19^. On the other hand, HiBiT fusions can be detected directly in living cells co-expressing LgBiT by bioluminescent imaging. To confirm localization of HiBiT fusions, the cell pools were transduced with BacMam containing an expression cassette for LgBiT, incubated for 24 h, and then imaged in the presence of substrate. Luminescence was visible in 49 of the 68 imaged HeLa cell pools (Figs. 4 and S7). Of the targets that were detectable by imaging, all were confirmed in the expected subcellular compartment. Together the HiBiT-specific blotting and imaging techniques were able to validate expression, size, and localization of the majority of HiBiT tagged proteins in the edited cell pools.

The HiBiT luminescence plate assays, blotting, and bioluminescence imaging demonstrate compatibility of the HiBiT tag with different analytical modalities for simplified cell validation. Multi-format analysis of HiBiT is possible in part due to its sensitivity which is sufficient to permit quantitative analysis of endogenously expressed HiBiT fusions in edited cells pools without the need for enrichment or subcloning. This is particularly valuable in situations where clonal isolation is challenging, such as when working with primary cells. It is also attractive for large-scale projects in which isolation of clones for each cell line is impractical and costly. Yet given the genetic diversity of cells within an edited pool, the stability of HiBiT positive cells over time remains unclear. Any adverse effect associated with editing could potentially reduce cellular fitness, leading to the loss of the edited population. To evaluate pool stability, luminescence was monitored over 62 days (equivalent of 15 passages) for 62 different Jurkat HiBiT-tagged pools (Fig. S8). Over this time, 36 pools showed less than a 3-fold reduction in specific signal over the course of the experiment and could still be used for quantitative analysis. With less time in culture (44 days), 52 out of 62 pools passed the same threshold. However, two of the pools, TUBA1A and H3F3A, exhibited a precipitous decline in signal by day 26. The protein products of TUBA1A and H3F3A, α-tubulin and histone H3.3, execute their biological functions either as a polymer or as part of a protein complex^20,21^. This suggests that in some situations even a small tag, such as HiBiT, may still interfere with protein function. Altogether, these data imply that the majority of pools are sufficiently stable to support experimental work for extended periods of time with the caveat that signal strength should be carefully monitored.

Although the blotting and imaging experiments validate the presence of functional fusion protein in the pool, the changes in luminescence observed over time serve as a reminder that cells within these populations harbor some level of genetic diversity. Double-strand break repair occurs through several different pathways, some of which will inevitably introduce unintended genetic lesions in the target gene. Of particular relevance is the NHEJ pathway which leads to random insertions and deletions at the cut site. These could affect function and expression of the target protein and contribute to a loss of cellular fitness. To unequivocally confirm successful integration of the HiBiT sequence at the intended loci and to assess the presence of other editing events such as indels or single nucleotide variations, next-generation sequencing (NGS) was performed on 4 of the cell pools (AKT1, FOS, IRAK4, and MAPK8). NGS-based deep sequencing of the amplicon spanning the cut site revealed substantial variability in the levels of HiBiT integration for different targets, ranging from an estimated 2–22% integration efficiency (Fig. S9a–d, Table S9). These variations support the notion that genomic context may play an important role in determining the repair response and the editing outcome^16^.

### Isolation and characterization of clonal cell lines

Although HiBiT-edited cell pools exhibit sufficient luminescence output and stability for many experimental needs, clonal cell lines are potentially beneficial where low integration rate, weak target expression, or poor pool stability limit signal strength. To explore the ease at which validated clonal lines can be generated and to assess the performance characteristics of these lines, clones of 15 different targets in 3 different cell lines were generated from the original heterogenous pools by single cell sorting. At approximately 3 weeks post-sorting, clones were screened for luminescence to identify those that carried the HiBiT insertion. In all cases, multiple positive clones were isolated and showed improvements in luminescence between 3- and 142-fold over the corresponding cell pool (Fig. S10a–c). Stability of all clonal lines was evaluated for at least 58 days by measuring luminescence during each passage. Over this time period, no clones lost luminescence which contrasts with the gradual decline in signal over passage that was observed with pools (Fig. S11a–c). For example, a pool of GSK3B-HiBiT edited Jurkat cells declined in luminescence by 57% over 60 days, while clonal GSK3B-HiBiT Jurkat cells showed no significant change in signal even after 134 days (37 passages). These results suggest that cell lines expressing HiBiT fusions at physiological expression levels can be established rapidly using a simple subcloning routine. In contrast to generation of stable cell lines by random integration, no antibiotic selection is required which makes the process faster and more efficient, and since only the endogenous locus is modified with high specificity, the introduction of biological artifacts associated with random integration is less likely.

In addition to stability testing, the clones were also evaluated at a genetic level. As indicated by the NGS analysis, pools of cells contain many different indels and single nucleotide variations in addition to properly integrated sequence. This means that individual clones may contain distinct editing events across the alleles that could impact the expression and function of the target from both the HiBiT-integrated and non-integrated alleles. Targeted amplicon sequencing using target specific primer pairs was performed on all clonal cell lines to confirm that at least one copy of the gene contained an in-frame, non-mutated HiBiT insertion and that non-integrated alleles did not contain mutations in the coding region of the gene (Table S10). Sequence validation may be particularly important if the HiBiT is introduced at N-terminus, where introduction of indels could lead to loss of expression from the impacted allele.

Although Sanger sequencing provides information about nucleotide sequences, it does not precisely quantitate how many copies of a particular sequence are present in a clonal population. This is highly relevant, as many immortalized cell lines, including the cell lines used in this study, exhibit genomic instability and polyploidy^22,23^. Droplet digital PCR (ddPCR) provides a more sensitive and quantitative method for determining if individual clones are homo- or heterozygous for the HiBiT tag. To demonstrate the value of ddPCR for genetic analysis of clones, at least one clone was analyzed by ddPCR for 11 different genes (Table S11, Fig. S12a–k) using target or HiBiT specific primers and probes. Of the multiple clones screened for AKT1 and FOS, each target had at least one homozygous clone in which all gene-specific amplifications were accompanied by HiBiT-specific amplification events. The remaining clones for these genes were heterozygous with 50% of the alleles containing HiBiT. In contrast, all isolated IRAK4 clones were heterozygous for insertion, with frequencies of approximately 33% and 68% suggesting a copy number of 3 for IRAK4. For the remaining 8 targets, ddPCR was used to establish zygosity of single clones. The results showed a variety in the number of alleles (2 to 4) for each target as well as percentage of HiBiT tagged alleles (25–100%), including homozygous clones for RIPK1, STAT3 and CASP3. The relatively high number of homozygous clones is not too surprising considering that the identification of clones relied on specific signal strength, which would favor clones with HiBiT insertion at all alleles. Overall, the ddPCR and sequencing results revealed expected differences in genetic composition at the target alleles of the clones. While many of the clones contained either proper HiBiT integration or indels that did not disrupt the coding region of the gene, there were situations in which mutations occurred in the CDS that could potentially disrupt the biology of the cell. Although clone generation is technically straightforward, genetic characterization is advisable to better understand zygosity and to ensure absence of mutations to coding or regulatory regions.

### Analyzing the effect of endogenous and plasmid-based expression on biological activity

Data presented thus far highlight the overall scalability of generating cell lines expressing endogenous HiBiT fusions. It is often assumed that these endogenous reporters better preserve biology than those that are overexpressed. However, data are lacking that directly compare protein functionality captured by endogenous expression with that measured using overexpression. To investigate whether cell models utilizing endogenously tagged proteins enable real time analysis of a biological process that cannot otherwise be observed using overexpression-based approaches, we selected two genes, FOS and NFKBIA, that are regulated transcriptionally and, in the case of NFKBIA, also post-translationally.

**Figure 5:**
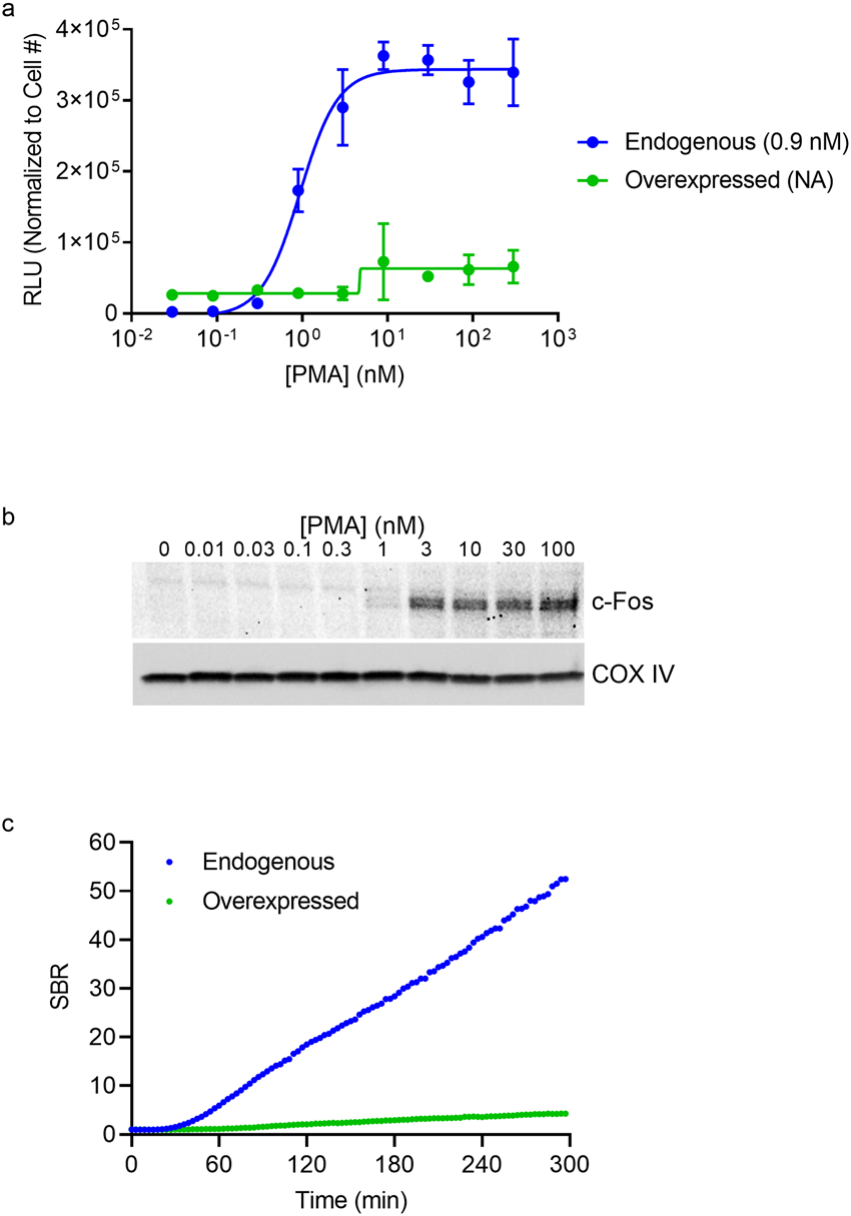
Analyzing c-Fos dynamics using endogenous and overexpressed HiBiT fusions. (**a**) c-Fos-HiBiT levels in K-562 cells following 4 h treatment with different concentrations of PMA and measured in lytic format. Data are expressed as mean luminescence values of three replicates normalized to cell number, with variability expressed as SD. EC_50_ values (0.9 nM for endogenous expression) are listed in parentheses. (**b**) Western blot of lysates from unedited K-562 cells that were treated with different concentrations of PMA for 4 h. Upper panel shows blot for c-Fos and lower panel shows blot for COX IV loading control. (**c**) Real-time measurement of c-Fos-HiBiT in K-562 cells treated with 30 nM PMA. In panels (**a**,**b**), blue represents endogenous expression of c-Fos-HiBiT from CRISPR-edited cells. Green represents plasmid overexpression of c-Fos-HiBiT.

The FOS gene, which belongs to the immediate early genes, rapidly and transiently expresses c-Fos in response to ERK/MAPK pathway activation. c-Fos dimerizes with c-Jun to form transcription factor AP-1 (activator protein 1) which controls the expression of numerous genes involved in differentiation, proliferation, and apoptosis. Overexpression of c-Fos and dysregulation of AP-1 have been linked to inflammatory diseases and cancer. The central role of transcriptional control for the biological function underscores the importance of studying this pathway in the appropriate physiological context without disrupting native regulatory mechanisms^24^. To determine if this tightly regulated pathway could be measured using an endogenous model, K-562 cells expressing c-Fos-HiBiT from the endogenous loci or from a plasmid were treated with various concentrations of phorbol 12-myristate 13-acetate (PMA) for 4 hours to activate the ERK/MAPK pathway. Basal levels of c-Fos-HiBiT were detected in lytic endpoint assays for both endogenous (SBR = 3) and plasmid transfected cells (SBR = 64) (Fig. 5a). However, endogenous cells showed a 182-fold increase in signal after treatment with 30 nM of PMA, while transfected cells failed to show a significant response (1.2-fold). The increase in c-Fos-HiBiT was dependent on PMA dose in the endogenously edited cells (EC_50_ = 0.9 nM) and was confirmed orthogonally by Western blotting (Figs. 5b and S13a–c). These results demonstrate that plasmid-based models, in which protein expression is regulated by constitutive promoters, are unsuitable for analysis of signaling pathways that depend upon transcriptional regulation. In contrast, cell models that utilize expression from endogenous loci leave native regulatory mechanisms intact and preserve the biological context of signaling. c-Fos-HiBiT expression was also monitored in real time to determine if the rapid activation of the FOS gene could be captured. As expected, levels of c-Fos-HiBiT expressed from the endogenous locus doubled within 30 min of PMA stimulation and increased 30-fold after 3 h (Fig. 5c)^25^. In the case of plasmid expression, no increase in c-Fos-HiBiT levels was observed at 30 min stimulation and only a 3-fold increase was detected after 3 h.

**Figure 6:**
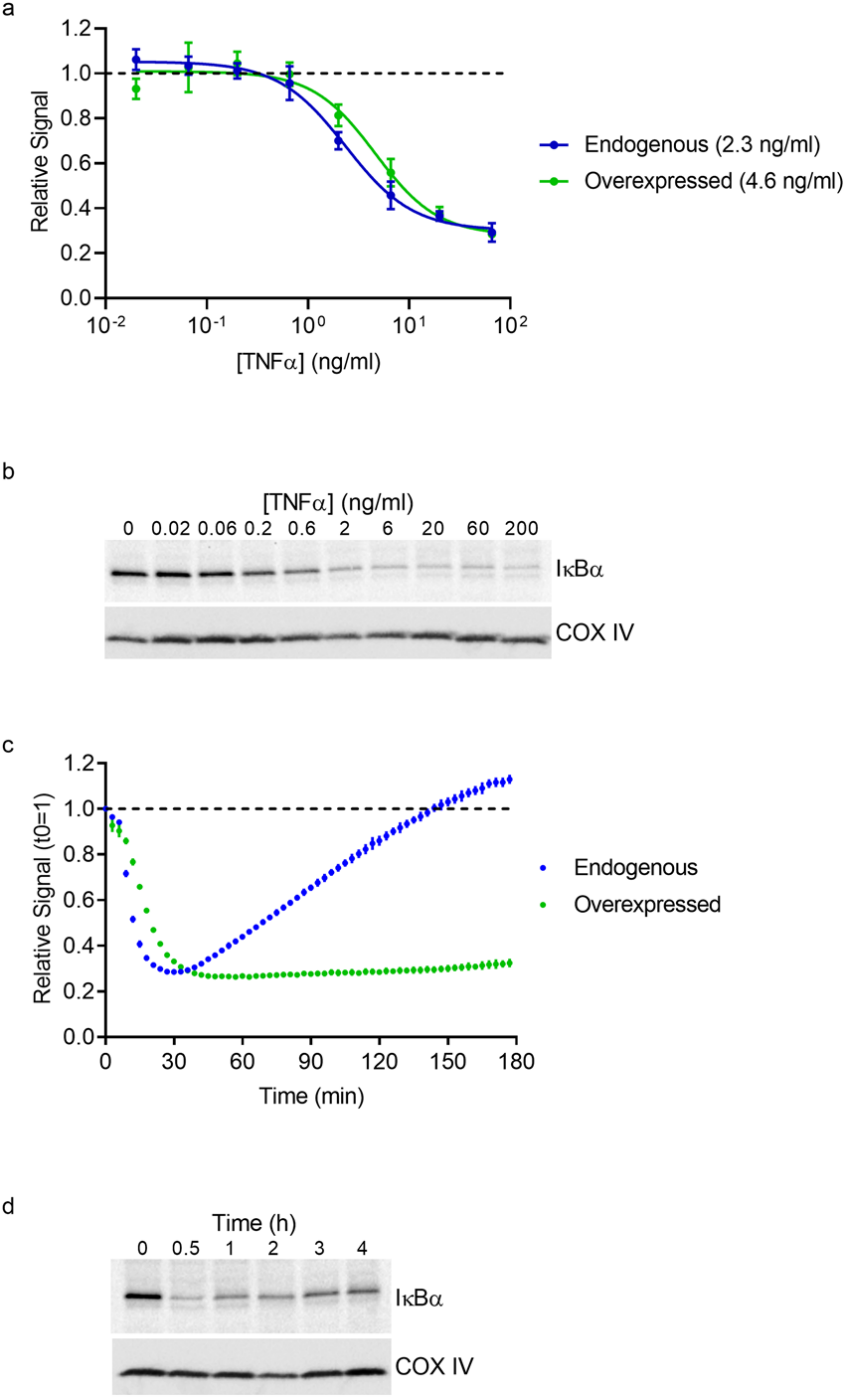
Quantitating IκBα dynamics using endogenous and overexpressed HiBiT fusions. (**a**) Changes in IκBα-HiBiT levels in HeLa cells expressing the fusion endogenously (blue) or from a plasmid (green) following incubation with different concentrations of TNFα for 30 min. Data were collected in lytic format in triplicate and are represented as luminescence relative to untreated cells, with error expressed as SD. IC_50_ values (2.3 ng/ml for endogenous cells and 4.6 ng/ml for overexpressed) are listed in parentheses. (**b**) Western blot of lysates from HeLa cells treated with different concentrations of TNFα for 30 min. Upper panel shows blot for IκBα and lower panel shows blot for COX IV loading control. (**c**) Real time measurement of IκBα-HiBiT in HeLa cells following treatment with 20 ng/ml TNFα. The data are expressed as signal relative to time at 0 s. Shown are the results of a representative experiment performed in triplicate with error expressed as SD. (**d**) Western blot of lysates from HeLa cells treated with 20 ng/ml TNFα for various times. The upper and lower panels show the blots for IκBα and for the COX IV loading control, respectively.

To further exemplify the value of cell models based on endogenous expression, NFKBIA, which encodes the protein IκBα, was selected as a second target for the comparative study. IκBα regulates the NF-κB pathway by binding and sequestering NF-κB in the cytosol. Pathway stimulation triggers sequential phosphorylation, ubiquitination, and degradation of IκBα. This releases NF-κB which then translocates into the nucleus and upregulates genes involved in immune system activation. The duration of NF-κB activity itself is controlled through NF-κB mediated upregulation of NFKBIA transcription. This establishes a negative feedback loop where the newly expressed IκBα will sequester and inactivate NF-κB^26^. To investigate this regulatory loop, a clonal HeLa cell line expressing IκBα-HiBiT, as well as cells transiently expressing IκBα-HiBiT, were treated with TNFα and luminescence was measured to determine total protein levels. Cells expressing IκBα-HiBiT from both the endogenous loci or from a plasmid showed dose-dependent declines in IκBα-HiBiT with similar IC_50_ values (IC_50_ = 2.3 ng/ml and 4.6 ng/ml, respectively) (Fig. 6a). This was consistent with the decline in IκBα protein levels observed by Western blotting in unedited cells (Figs. 6b, and S14a,b). Although both edited and transfected cells showed similar decreases in IκBα-specific signal, differences between the models became apparent when studying dynamics of IκBα recovery, which is initiated through NF-κB-mediated upregulation of IκBα transcription. Cells edited or transfected to express IκBα**-**HiBiT were transduced with BacMam-LgBiT, and luminescence was monitored in real time following stimulation with TNFα. While IκBα-HiBiT declined by comparable magnitude in both cell lines, maximum IκBα-HiBiT degradation was achieved faster in the endogenous line compared to the overexpressed cells (30 min versus 40 min, respectively). Significantly, the subsequent recovery of IκBα-HiBiT protein was only captured in the edited cells whereas the overexpression system showed little recovery of IκBα-within the timeframe (Fig. 6c). Western blotting of unedited cells treated with TNFα confirmed that IκBα dynamics observed with IκBα-HiBiT in edited cells are consistent with the kinetics of unmodified IκBα (Figs. 6d and S14a,b). The increase in signal displayed by the edited cells therefore directly reflects the TNFα-mediated upregulation of the endogenous NFKBIA promotor leading to increased expression of IκBα-HiBiT. In contrast, any change of IκBα-HiBiT levels in transfected cells is a consequence of constitutive expression from the plasmid and is untethered from physiological regulatory mechanisms. This clearly demonstrates the power of endogenous reporter expression for accurately capturing live cell, kinetic processes. This becomes especially vital in situations where kinetics are rapid, biphasic, or depend upon transcriptional or post-translational events.

## Discussion

In this study, we demonstrate that the HiBiT luminescent peptide can be integrated at a variety of loci to generate endogenously expressed protein fusions. While endogenous HiBiT tagging was originally published as a means to detect five different hypoxia-related proteins in two cell lines, the current study establishes the scalability of the approach by showing that 83 of 97 targets among four cell lines could be HiBiT tagged and detected by luminescence in a single experiment. While the majority were successfully tagged by following set limitations of termini placement (C-terminus) and number of gRNA tested (two per edit), these conditions may not be suitable for all proteins. To achieve tagging of an entire set of proteins, an individualized approach may be required for those missed in the original screen. As shown in this study, integration at a desired locus can be significantly impacted by the choice of gRNA, where at times SBR differed significantly between the two guides. This was evident for EZH2 in K-562 cells, where one gRNA led to a SBR of 466, while use of the other resulted in no apparent HiBiT integration. Therefore, successful tagging may require screening of additional gRNA with different PAM sites. For other targets, the position of the tag (N-terminal, C-terminal, or internal) may need to be evaluated. In this report, tagging of MMP14 was unsuccessful at either terminus but was accomplished by inserting HiBiT internally. Follow up analysis of the remaining missed targets would provide insight into the frequency at which the set editing parameters contributed to the inability to detect luminescence.

Overall, endogenous HiBiT tagging achieves a level of scalability not likely to be encountered with other protein analysis techniques, including immunodetection of individual proteins. Investigating the same targets featured in this study by Western blotting would require 97 antibodies with each potentially requiring optimization. Thus, experimentation becomes time-consuming, expensive, and in cases where no antibody exists, impossible. Large-scale endogenous tagging could theoretically be achieved with a small epitope tag such as FLAG. Yet, detection of epitope tags comes with the limitation that antibodies are required which immediately reduces analysis to endpoint in cell lysates. A key advantage to this method is that it permits live cell, kinetic analysis. Preserving the integrity of the cell enables one to study signaling pathways in a native environment without overexpression and the associated potential for artifacts. This report illustrates the significance of this by comparing data obtained using HiBiT fusions expressed from either the endogenous loci or from a plasmid of two transcriptionally regulated proteins, IκBα and c-Fos. In both cases, only endogenous expression accurately captured the proper protein dynamics. In essence, functional assays that more accurately capture cellular protein dynamics can be achieved on a large-scale with minimal effort.

The scalability and ability to quantify steady-state and kinetic events are two aspects of HiBiT endogenous tagging that differentiate it from immunoassays and epitope tagging. This potentially enables studies of entire protein families and full signaling pathways in such a way as to preserve critical cellular functions, including alternative splicing, post-translational modifications, or transcriptional regulation. Although the targets for this report were selected to represent the diversity of the proteome, they primarily fall into the category of intracellular proteins. Application of this workflow to secreted, extracellular matrix, and transmembrane receptors remains to be demonstrated. However, no principal deficiencies of this approach have been identified so far and successful tagging of endogenous GPCRs with HiBiT has been reported^27^. Overall, this suggests that the HiBiT tagging strategy could be extended to all protein families, including those that are not strictly intracellular.

## Methods

### Cell culture, transfection, and treatments

Immortalized cell lines were obtained from American Type Culture Collection and maintained in growth medium supplemented with 10% fetal bovine serum (Seradigm) at 37 °C and 5% CO_2_. All medium, trypsin-EDTA (0.05%), and Dulbecco’s phosphate buffered saline (DPBS) were obtained from ThermoFisher (Gibco). HeLa cells (CCL-2) were grown in Dulbecco’s Modified Eagle’s Medium, K-562 cells (CCL-243) were grown in Iscove’s Modified Dulbecco’s Medium, and Jurkat cells (TIB-152) were grown in RPMI 1640. For overexpression experiments, cells were transfected with plasmids encoding C-terminal HiBiT fusions to c-Fos and IκBα under control of the HSV TK promoter. The transfection was prepared by mixing and incubating 0.5 µg of plasmid and 4.5 µg pGEM3Z carrier DNA (Promega) with 20 µl FuGENE HD Transfection Reagent (Promega) for 10 min at room temperature in 500 µl OptiMEMI. The transfection was then added to 10 ml cell suspension (2 × 10^5^ cells/ml) and plated in 10 cm tissue cultures dishes for 24 h. In some experiments, cells were grown in the presence of varying concentrations of phorbol 12-myristate 13-acetate (PMA, Promega) or human recombinant tumor necrosis factor-α (TNFα, Promega).

### Generation of activated primary human T cells

Primary human T cells were isolated from whole blood using RosetteSep (STEMCELL Technologies) for negative selection followed by density centrifugation using Lymphoprep (STEMCELL Technologies) following manufacturer’s instructions. The cells were washed once in growth medium (RPMI supplemented with 10% FBS) and resuspended in growth medium at a density of 1 × 10^6^ cells per ml. The T cells were activated by adding 25 μl of ImmunoCult Human CD3/CD28 T Cell Activator (STEMCELL Technologies) per 1 ml of culture followed by 3 days of cultivation at 37 °C and 5% CO_2_.

### Human blood collection and compliance with ethical standards

Whole blood (8 tubes × 8 ml) was collected by the Promega Wellness Center following the regulations stated in Promega Protocol 16 which was approved by the Promega Corporation Human Subjects Review Board. Regulations approved by the Promega Corporation Human Subjects Review Board follow HHS regulations 45 CFR Part 46. Written informed consent was obtained from all donors.

### Cas9, gRNA, and donor DNA

Alt-R S.p. Cas9 Nuclease V3, Alt-R CRISPR RNA (crRNA), Alt-R transactivating crRNA (tracrRNA), Nuclease-Free Duplex Buffer, and Ultramer DNA Oligonucleotides (ssODN) were obtained from Integrated DNA Technologies (IDT). Cas9 was diluted to 20 µM in Nuclease-Free Duplex Buffer. gRNA was prepared by incubating 1 nmol crRNA, 1 nmol tracrRNA, and Nuclease-Free Duplex buffer (final volume 50 µl) at 95 °C for 5 min and then cooling to room temperature. ssODN donor templates were resuspended to 100 µM in nuclease-free water. Target-specific crRNA and ssODN sequences can be found in Tables S2 and S3.

### Generation of HiBiT knockin pools

RNP complexes were assembled by incubating 100 pmol Cas9 and 120 pmol gRNA in a final volume of 10 µl Nuclease-Free Duplex Buffer for 10 min at ambient temperature. 2 × 10^5^ cells were resuspended in 20 µl of 4D Nucleofector Solution SE (Lonza) for Jurkat and HeLa cells, Nucleofector Solution SF for K-562 cells, and Nucleofector Solution P3 Primary Cell for T cells. RNP complex and 100 pmol of donor DNA were added to the cell suspension followed by electroporation with the 4D Nucleofector System (Lonza) using programs CN-114 for HeLa, FF-120 for K-562, CL-120 for Jurkat, and FI-11S for T cells. Cells were then incubated at ambient temperature for 5 min and transferred to a six-well plate containing 2 ml growth medium. At 72 h post-electroporation, cells were analyzed for insertion.

### Lytic HiBiT detection

2 × 10^4^ cells were plated in solid white 96-well tissue culture plates (Corning 3917) in 100 µl growth medium. 24 h after seeding, HiBiT was detected using the Nano-Glo HiBiT Lytic Detection System (Promega) following manufacturer’s instructions. Briefly, 100 µl of Nano-Glo HiBiT Lytic Detection Reagent was added directly to the cells and incubated for 5 min on an orbital shaker (300 rpm) before recording luminescence on a GloMax Discover (Promega) with 0.2 s integration time.

### Live cell HiBiT detection

2 × 10^4^ cells were plated in solid white 96-well tissue culture plates in 100 µl growth medium containing 5% (v/v) BacMam CMV-LgBiT reagent (Kempbio, Inc., viral titer approximately 2 × 10^8^ PFU/ml) and grown for 24 h. For single read experiments, cells were washed with DPBS and then incubated with 100 µl of CO_2_-Independent Medium (Gibco) containing Nano-Glo Luciferase Assay Substrate (Promega) per manufacturer’s protocol. For multi-timepoint reads, cells were pre-incubated with 100 µl of CO_2_-Independent medium containing Vivazine Live Cell Substrate (Promega) for 45 min prior to treatments followed by luminescence measurements on a GloMax Discover set to 37 °C with 0.2 s integration time.

### Bioluminescent imaging

5 × 10^4^ cells were grown in 400 µl growth medium containing 5% (v/v) BacMam-LgBiT on an 8-well chambered coverglass (Nunc Lab-Tek II) for 24 h. The medium was replaced with 100 µl of CO_2_-Independent Medium containing Vivazine Live Cell Substrate (1:100 dilution) and incubated at 37 °C for 45 min before imaging. Images were acquired on the LV200 bioluminescence imaging system (Olympus) equipped with an ImagEM X2 EM-CCD camera (Hamamatsu) and a temperature-controlled stage. All images were captured with cellSens software (Olympus) with the indicated objectives, electron multiplying (EM) gain, and exposure settings (Table S8.) Further analysis and processing of images was performed using Fiji and was limited to pseudo-coloring and linear adjustments of contrast and brightness^28^.

### HiBiT and Western blotting

Cells were grown to confluence in a 6-well culture plate, harvested using trypsin-EDTA, and pelleted at 180 × g for 5 min. Cell pellets were resuspended in 50 μl Mammalian Lysis Buffer (Promega) supplemented with Protease Inhibitor Cocktail (Promega) and RQ1 RNase-Free DNase (Promega) and incubated on a shaker for 20 min at ambient temperature. Lysate was diluted with equal volume of 2X SDS-PAGE gel loading buffer (120 mM Tris-HCl, pH 6.8, 1.5 mM bromophenol blue, 25% glycerol, 200 mM dithiothreitol, and 1% SDS), heated at 70 °C for 5 min, and separated on a 4–20% SDS-PAGE gel. For HiBiT blotting, proteins were transferred to a nitrocellulose membrane using the iBlot 2 Gel Transfer Device (ThermoFisher Scientific) and incubated in TBST containing 1 mM dithiothreitol (DTT) for 30 min at ambient temperature with shaking. The membrane was transferred to 1X Nano-Glo Blotting Buffer (Promega) containing a 1:200 dilution of LgBiT protein (Promega) and incubated for 1 h at ambient temperature. Nano-Glo Luciferase Assay Substrate (Promega) was added to the LgBiT-containing buffer at a 1:200 dilution, and the membrane was incubated an additional 5 min. Luminescence was detected using the ChemiDoc MP Imaging System (BioRad) equipped with Image Lab 5.2.1 software. HiBiT blots were analyzed using Fiji with linear contrast adjustments made to view each protein band. The same blots were also imaged in colorimetric mode to visualize the molecular weight standard (Thermo Scientific Spectra Multicolor Broad Range Protein Ladder). For Western blotting, proteins were transferred to PVDF membrane and blocked in 5% bovine serum albumin (Promega) in TBST. Membranes were probed overnight at 4 °C with the rabbit anti-human c-Fos (9F6) antibody (1:1000 dilution, Cell Signaling), the mouse anti-human IκBα (L35A5) antibody (1:1000 dilution, Cell Signaling), or the rabbit anti-human COX IV (3E11) loading control antibody (1:2000 dilution, Cell Signaling). Membranes were incubated with peroxidase conjugated AffiniPure donkey anti-rabbit or anti-mouse IgG secondary antibodies (1:5000 dilution, Jackson ImmunoResearch) for 1 h at ambient temperature, and chemiluminescence was detected using the ECL Western Blotting Substrate (Promega) in combination with the ChemiDoc MP Imaging System. Spectra Multicolor Broad Range Protein Ladder (ThermoFisher Scientific) was loaded on each gel and imaged on the blots with the ChemiDoc MP Imaging System in colorimetric mode. Colorimetric and luminescence images were superimposed to indicate molecular weight of detected proteins.

### Single cell cloning

Pools of edited cells were resuspended to 5 × 10^6^ cells per ml in sorting buffer (Hank’s Balanced Salt Solution, 10 mM HEPES, 0.2% bovine serum albumin, and 10 units per ml of penicillin-streptomycin, passed through a 35 µM mesh filter (Corning Falcon) to disperse clumps, and loaded onto the BD FACSMelody (BD Biosciences) cell sorter. Single cells were sorted into each well of solid white 96 well tissue culture plates (Corning 3917) containing 150 µl of growth medium per well. Cells were grown at 37 °C and 5% CO_2_ until colonies formed (approximately 3 weeks). HeLa cells were screened using the live cell HiBiT detection assay (described above), and luminescence-positive colonies were expanded for additional analyses. To screen Jurkat and K-562 colonies for HiBiT, replica plates were generated by transferring 50 µl of cell suspension to new plate. Luminescence in the replica plated cells was immediately measured using the lytic HiBiT detection assay (described above), and luminescence positive clones from the corresponding well in the parental plate were expanded.

### Amplicon sequencing

Genomic DNA was purified from 4 × 10^5^ cells using the Maxwell RSC Cultured Cells DNA Kit (Promega) and the Maxwell CSC instrument (Promega), according to manufacturer’s protocol. Primers were designed to span the donor template sequence, resulting in amplicons of 400–500 nucleotides (Table S12). 50 ng of DNA was PCR amplified with 10 µM primers to contain homology arms to pF5 CMV-neo Flexi Vector (Promega) and cloned using Gibson Assembly with the HiFi 1-Step Gibson Kit (SGI-DNA, Inc.). Plasmids were transformed into JM109 Competent Cells (Promega). To ensure representation of different editing events, 24 colonies from each plate were picked, purified using the PureYield Plasmid Miniprep System (Promega), and Sanger sequenced by Functional Biosciences (Madison, WI).

### Droplet digital PCR

Primers were designed to span the integration site outside of the repair template sequence and resulted in amplicons ranging from 150 to 400 base pairs (Table S13). Hydrolysis probes were designed for both the HiBiT and target gene. 30 ng of genomic DNA was digested with 10 units of XbaI (Promega) for 10 min and then added to ddPCR Supermix for Probes (No dUTP) (BioRad) containing 900 nM primers and 250 nM probes. Droplets were formed using BioRad DG8 Cartridges and the QX200 Droplet Generator, per manufacturer’s protocol. Reactions were amplified with these conditions: 95 °C for 1 min followed by 40 cycles of 94 °C for 30 sec and 58 °C for 1 min and 98 °C for 10 min. Following amplification, droplets were analyzed on the QX200 Droplet Reader (BioRad) and the QuantaSoft Software. The ratio of HiBiT-positive droplets to total target-positive droplets was used to calculate percent HiBiT integration and allelic frequency.

### Next-generation sequencing

100 ng of genomic DNA was amplified using the 16 S Metagenomics Sequencing Library Preparation protocol (Illumina, 15044223 Rev. B). Each reaction contained 12.5 µl of 2X KAPA HiFi HotStart ReadyMix (Roche), 200 nM primers, and DNA in final volume of 25 µl. Cycling conditions were as follows: 95 °C for 3 minutes; 25 cycles of 95 °C for 30 seconds, 60 °C for 30 seconds, and 72 °C for 30 seconds; 72 °C for 5 minutes; hold at 4 °C. Primers were the same as those used for ddPCR with universal primer tails TCGTCGGCAGCGTCAGATGTGTATAAGAGACAG (forward primer) and GTCTCGTGGGCTCGGAGATGTGTATAAGAGACAG (reverse primer). Reactions were purified using the ProNex Size-Selective Purification System (Promega) using the manufacture’s protocol for dual size-selection with chemistry ratios of 1.24X and 1.64X final (0.4X added) and eluted in 26 µl Elution Buffer. Indices and Illumina sequencing adaptors were added in a second stage PCR, using the Nextera XT Index Kit (Illumina) according to the 16 S Metagenomics Sequencing Library protocol (Illumina), with the following cycling conditions: 95 °C for 3 minutes; 8 cycles of 95 °C for 30 seconds, 55 °C for 30 seconds, and 72 °C for 30 seconds; 72 °C for 5 minutes; hold at 4 °C. PCR reactions cleaned up using ProNex Size-Selective Purification System with the manufacturer’s protocol for single-sided selection and a 2X chemistry ratio. Final libraries were eluted with 27.5 µl Elution Buffer and quantified using QuantiFluor ONE dsDNA System (Promega) and the ProNex NGS Library Quant Kit (Promega). Amplicon and library sizes were assessed using D1000 High Sensitivity or D1000 ScreenTapes on the 4200 TapeStation (Agilent). A final pooled library with samples at 4 nM was diluted and denatured as described in the 16 S Metagenomics Sequencing Library Protocol. PhiX Control v3 (Illumina) was added to 5% and final library was loaded at 10 pM in a MiSeq v3 Kit (600-cycle) cartridge (Illumina) for paired end reads (2 × 300 bp) on a MiSeq System (Illumina). Primary data analysis was performed by the University of Wisconsin-Madison Bioinformatics Resource Center. For this analysis, FASTQ files were analyzed for each sample using CRISPRESSO with a custom Python3 script. The custom script combines all reads with identical sequences for SNPs, insertions, and deletions relative to the genome, as well as the presence and direction of HiBiT insertions.

## Supplementary information

Supplementary information.
