# Supplementary material for: A Simple and Scalable Strategy for Analysis of Endogenous Protein Dynamics
Source: Sci Rep. 2020 Jun 2;10:8953. doi: 10.1038/s41598-020-65832-1 (PMC7265437; doi:10.1038/s41598-020-65832-1)
Supplement: Supplementary file 1 — Supplementary information. [file 41598_2020_65832_MOESM1_ESM.pdf]

Supplementary Information for:

A Simple and Scalable Strategy for Analysis of Endogenous Protein Dynamics

Marie K. Schwinn[*,†], Leta S. Steffen[†], Kris Zimmerman[†], Keith V. Wood[‡], and Thomas Machleidt[†]

[†]Promega Corporation, Madison, Wisconsin 53711 United States
[‡]Light Bio, Inc., Madison, Wisconsin 53711 United States

[*]To whom correspondence should be addressed:
2800 Woods Hollow Road, Madison, Wisconsin, 53711 United States
Email: marie.schwinn@promega.com

Supplementary Table S1. Gene targets with corresponding transcript expression levels and protein sizes, functions, and subcellular localizations. Expression data were obtained from the Human Protein Atlas version 19.1 (http://www.proteinatlas.org) and are reported as normalized expression (NX). Protein-specific data were gathered from The UniProt Consortium (http://www.uniprot.org).

Gene	NX (HeLa)	NX (K562)	MW (kDa)	Function	Localization
ACTB	145.3	71.9	41.7	Structural	Cytoskeleton
AKT1	18.1	12.1	55.7	Signaling	Cytoplasm, Nucleus, Membrane
APAF1	6.3	5.6	141.8	Apoptosis	Cytoplasm
ARIH1	12.2	7.2	64.1	Metabolism	Cytoplasm, Nucleus
ARNT	6.5	7.0	86.6	Signaling	Nucleus
ARRB2	9.9	2.7	46.1	Signaling	Cytoplasm, Nucleus, Membrane, Vesicle
ATM	4.7	8.2	350.7	Signaling	Nucleus, Vesicles
ATP1B3	32.5	22.7	31.5	Metabolism	Membrane
AXL	14.0	2.0	98.3	Signaling	Membrane
BAD	27.2	3.2	18.3	Apoptosis	Mitochondria, Cytoplasm
BCL2	0.6	0.2	26.3	Apoptosis	Mitochondria, Nucleus, ER
BRCA1	19.8	25.4	207.7	Metabolism	Nucleus, Cytoplasm
CALR	56.0	53.2	48.1	Metabolism	ER, Cytosol, Extracellular Matrix
CASP3	3.6	15.1	31.6	Apoptosis	Cytoplasm
CAT	7.3	26.5	59.8	Metabolism	Peroxisome
CAV1	8.9	0.0	20.5	Structural	Golgi, Membrane
CCND1	4.7	0.0	33.6	Metabolism	Cytoplasm, Nucleus, Cytoskeleton, Membrane
CENPA	3.2	12.7	16.0	Metabolism	Nucleus
CFL1	92.3	62.6	18.5	Structural	Nucleus, Cytoskeleton, Membrane
CREBBP	13.2	13.7	265.4	Epigenetics	Cytoplasm, Nucleus
CTNNB1	40.1	21.2	85.5	Signaling	Cytoplasm, Nucleus, Membrane
CUL1	16.9	38.4	89.7	Metabolism	Cytoplasm, Nucleus
CYCS	28.6	75.6	11.7	Metabolism	Mitochondria
EEA1	3.5	7.3	162.5	Structural	Cytoplasm, Endosome
EPAS1	1.8	0.5	96.5	Signaling	Nucleus
ESR2	0.7	0.6	59.2	Signaling	Nucleus
EZH2	9.8	27.1	85.4	Epigenetics	Nucleus
EZR	28.9	17.3	69.4	Structural	Membrane, Cytoskeleton
F2R	0.7	4.2	47.4	Signaling	Membrane
FGFR1	1.5	2.2	92.0	Signaling	Membrane, Nucleus, Cytosol, Vesicle
FOS	3.8	0.4	40.7	Signaling	Nucleus, ER, Cytosol
FOXO3	2.9	9.7	71.2	Signaling	Cytosol, Nucleus, Mitochondria
GABBR1	3.4	6.6	108.3	Signaling	Membrane
GJA1	2.9	0.0	43.0	Structural	Membrane, ER
GOLGA2	34.0	23.8	113.1	Structural	Golgi
GSK3B	10.3	7.1	46.7	Signaling	Cytoplasm, Nucleus, Membrane
H3F3A	45.5	90.1	15.3	Epigenetics	Nucleus
HDAC2	21.7	62.0	55.4	Epigenetics	Nucleus, Cytoplasm

HDAC6	16.2	23.1	131.4	Epigenetics	Nucleus, Cytoplasm
HIST1H4A	1.5	1.3	11.4	Epigenetics	Nucleus
HSP90B1	48.5	49.8	92.5	Metabolism	ER
IL12A	0.1	0.0	24.9	Signaling	Secreted
IL15	13.7	3.8	28.2	Signaling	Secreted, Cytoplasm, Nucleus
IRAK4	3.0	8.0	51.5	Signaling	Cytoplasm
IRS1	0.9	1.6	131.6	Signaling	Cytosol, Nucleus, Membrane
ITGB1	33.2	10.6	130.8	Signaling	Membrane
JUN	0.9	3.7	35.9	Signaling	Nucleus
KDM1A	26.0	34.4	92.9	Epigenetics	Nucleus
KLC1	26.0	18.0	65.3	Structural	Vesicle, Cytoskeleton
KPNB1	59.4	69.6	97.2	Structural	Cytoplasm, Nucleus
KRAS	7.9	11.2	21.7	Signaling	Membrane, Cytosol
LAMP1	30.1	14.1	44.9	Structural	Membrane, Lysosomes, Endosomes
MAPK8	5.0	13.1	48.3	Signaling	Cytoplasm, Nucleus
MARK3	17.1	21.0	88.4	Signaling	Membrane, Cytoplasm
MC1R	3.0	1.8	34.7	Signaling	Membrane
MET	27.1	0.9	155.5	Signaling	Membrane
MMP14	0.1	0.1	65.9	Metabolism	Membrane, Cytoplasm
MTOR	28.6	25.1	288.9	Signaling	Cytoplasm, Nucleus
NFE2L2	22.3	14.5	67.8	Signaling	Cytosol, Nucleus
NFKBIA	8.3	3.2	35.6	Signaling	Cytoplasm, Nucleus
NIFK	12.2	43.5	34.2	Signaling	Nucleus
NOTCH1	3.0	1.4	272.5	Signaling	Membrane, Cytoplasm
NR3C1	27.8	3.3	85.7	Signaling	Cytoplasm, Nucleus
NUMB	9.5	13.9	70.8	Structural	Membrane
NUP98	19.2	38.9	97.8	Structural	Nucleus
PARP1	20.4	51.2	113.1	Metabolism	Nucleus
PDGFRA	0.1	0.1	122.7	Signaling	Membrane
PEX5	8.8	12.3	70.9	Structural	Cytoplasm, Peroxisome
PLCG1	11.4	8.9	148.5	Signaling	Cytosol, Membrane
PPARG	1.9	0.0	57.6	Signaling	Nucleus, Cytoplasm
PRKAB1	5.6	37.9	30.4	Signaling	Cytosol, Nucleus
PRKCA	7.3	8.0	76.8	Signaling	Cytoplasm, Membrane
PTEN	12.8	3.1	47.2	Signaling	Cytoplasm, Nucleus
PTK2	12.9	19.8	119.2	Signaling	Membrane, Cytoskeleton, Nucleus
PTPN11	31.0	40.7	68.4	Signaling	Cytoplasm, Nucleus
RAC1	27.2	37.4	21.5	Signaling	Membrane, Cytoplasm
RB1	10.1	7.1	106.2	Signaling	Nucleus
RHOA	57.0	41.0	21.8	Signaling	Membrane, Cytoskeleton
RIPK1	4.5	13.0	75.9	Signaling	Cytoplasm
RPS3	53.0	122.1	26.7	Metabolism	Cytoplasm, Nucleus, Mitochondria
SEC22B	18.6	4.6	24.6	Structural	ER, Golgi
SERP2	0.0	0.0	7.4	Metabolism	Membrane
SIRT2	5.8	5.2	43.2	Epigenetics	Nucleus, Cytoplasm, Cytoskeleton
SMAD2	48.7	13.7	52.3	Signaling	Cytoplasm, Nucleus

SMURF1	4.6	7.0	86.1	Signaling	Cytoplasm, Membrane
STAT3	19.3	22.2	88.1	Signaling	Cytoplasm, Nucleus
TNFRSF1A	16.0	6.2	50.5	Signaling	Membrane, Golgi, Secreted
TNFSF10	0.4	0.0	32.5	Signaling	Membrane, Secreted
TOMM20	25.4	56.0	16.3	Metabolism	Mitochondria
TP53	20.0	0.9	46.7	Signaling	Cytoplasm, Nucleus
TUBA1A	2.0	3.1	50.1	Structural	Cytoskeleton
TUBG1	41.0	24.6	51.2	Structural	Nucleus
VAMP1	10.2	0.5	12.9	Structural	Membrane
VASP	8.0	24.3	39.8	Structural	Cytoplasm, Cytoskeleton
VCL	15.8	8.1	123.8	Structural	Membrane, Cytoskeleton
VDR	1.3	0.7	48.3	Signaling	Nucleus
VHL	10.5	16.4	24.2	Signaling	Cytoplasm, Nucleus

Supplementary Table S2. Guide RNA sequences.

Gene	Guide 1	Guide 2
ACTB	CCACCGCAAATGCTTCTAGG	AGTCCGCCTAGAAGCATTTG
AKT1	AGCGGCACGGCCTGAGGCGG	GAGGCGGCGGTGGACTGCGC
APAF1	AACTTTTTAAATTTTTGAAT	TATGTGACTGTGGATAATCT
ARIH1	TTCAGAGTTCATTTTATGCA	AGTACATTGAGGACTGAGAA
ARNT	CCTTTTCAGAATAGAACTAT	GAACTATTGGGGTGAGGATA
ARRB2	AGGAAGCGGGGTGGGAAGAA	TGATCAACTCTGCTAGGAAG
ATM	TTTCTAAAGGCTGAATGAAA	TTTCCCAGGATGGAAAGCTT
ATP1B3	ACAACACAACATTTACTCTG	AGCACGTGCATAGTATGAGT
AXL	TCCTGAGAGGGAGTACCAGG	GAGAGGGAGTACCAGGTGGA
BAD	AGTTTCGGGATGTGGAGCGA	GGATGTGGAGCGAAGGTCAC
BCL2	TTTGCATATTTGTTTGGGGC	AGGCATGTTGACTTCACTTG
BRCA1	TGGCTGGCTGCAGTCAGTAG	TACTGACTGCAGCCAGCCAC
CALR	GCCTCTCTACAGCTCGTCCT	GCCAAGGACGAGCTGTAGAG
CASP3	TCACTAAAGAAATGGTTGGT	TTTATCACTAAAGAAATGGT
CAT	AGCTTCGCTGCACAGGTGCA	AAGGCAAATCTGTGAGGCCG
CAV1	GAAATATAAATGACATTTCA	TTTCTTTCTGCAAGTTGATG
CCND1	TCTGAGGGCGCCAGGCAGGC	CGTGGACATCTGAGGGCGCC
CENPA	CGGGGCCTTGAGGAGGGACT	CTGACAGAAACACTGGGTGC
CFL1	CTCCAGGCAGGGGGCCAGAA	AAGCCTTTGTGAGCCCCTTC
CREBBP	ACGCTAGAGAAGTTTGTGGA	CCAAGAACATGAAAGGGAAA
CTNNB1	TTTTAAAACTTCTTACCTAA	TGACCTGTAAATCATCCTTT
CUL1	CACAGTCAGACCCTTCCAGA	TTGGCTTAACCCTTCTGGAA
CYCS	TTTTGTAATAAATAAGGCAG	AGCTACTAATGAGTAATAAT
EEA1	CAATGACTTGCAAGGATAAT	TCAATGACTTGCAAGGATAA
EPAS1	TGGACCAGGCCACCTGAGCC	GGTAGAAGGCCTGGCTCAGG
ESR2	TGACCCTCTAATCAACTCGG	CAGTGACCCTCTAATCAACT
EZH2	GCTGTTTCAGAGGAGGGGGG	GGAGGAGGTAGCAGATGTCA
EZR	TTCGAGGCCCTGTAACAGCC	AACAGCCAGGCCAGGACCAA
F2R	ACTTAGGAAAAGGGACTGCT	AAGCTGTTAACTTAGGAAAA
FGFR1	GGCGTGTGGGTGGCAGTCAG	GTCTGGGGAGGGCGTGTGGG
FOS	GCTGGCCCTGTGAGGGGGCA	CCTGTGAGGGGGCAGGGAAG
FOXO3	GCCAGGCTGAAGGATCACTG	GCTGAAGGATCACTGAGGAA
GABBR1	TTATAAGTGAGGGTAGGGTG	TTTGCTTTATAAGTGAGGGT
GJA1	TGACCTGGAGATCTAGATAC	AAGCCTGTATCTAGATCTCC
GOLGA2	GATCACTGTCATCTAAAAGC	CAGGCTTTGCTGACAGTAGC
GSK3B	CGGGACTGTTCAGGTGGAGT	GCTGCTCGGGACTGTTCAGG
H3F3A	TGCTTAAGAATCCACTATGA	TGAAATGTTTCCCATCATAG
HDAC2	GGTGAGACTGTCAAATTCAG	TTTTAATGATTTTCTGAAAT
HDAC6	CCACACTAAGCCCCAGAATA	CGTATTCTGGGGCTTAGTGT
HIST1H4A	CTTTATGGCTTTGGCGGTTA	TGGTTCAGAAATGCAAGCTG
HSP90B1	CTCTCCACACAGGATCCAAA	TAAATTATACTCTCACCATT
IL12A	TTGGAGGGACCTCGCTTTTT	TGAATGCTTCCTAAAAAGCG
IL15	AAGAAGTGTTGATGAACATT	
IRAK4	ACAGCTTCTTAAAACTTTAT	

IRS1	ATGTCCAGTTGAGCTACTGA	AGGACCGTCAGTAGCTCAAC
ITGB1	ACTCATTTTCCCTCATACTT	GTGGTCAATCCGAAGTATGA
JUN	TTTTGAAGAGAGACCGTCGG	ACATTTTGAAGAGAGACCGT
KDM1A	ATGCATCTGTCTCACATGCT	TGTGAGACAGATGCATTCTA
KLC1	CACCGCTAACGTGAGTCCCA	GGCCGTGGGACTCACGTTAG
KPNB1	AGGCAAGATCTTACCAAGCT	GGAAACTGAAGAACCAAGCT
KRAS	TTAAGGCATACTAGTACAAG	CAATTTGTACTTTTTTCTTA
LAMP1	GGCTACCAGACTATCTAGCC	GTGCACCAGGCTAGATAGTC
MAPK8	TTGACAGACGACGATGATGA	ACAGGTGCAGCAGTGATCAA
MARK3	GGGTTACAGCTTTAGCTCAT	TAATTTACATCATAATCACT
MC1R	TGACATGCTCCTGGTGAGCG	CCTGGTGAGCGCGGTGCACG
MET	CACACGACCAGCCTCCTTCT	ACACACGACCAGCCTCCTTC
MMP14	CAAGGTCTGACGCCCACCGC	GGCGTCAGACCTTGTCCAGC
MMP14 (internal)	CTCAACCCAGGACTACCTCC	
MTOR	TAGGTGCCCTTTCTGGTAAC	GTGCCCTTTCTGGTAACTGG
NFE2L2	ATCTAGTTTTTCTTAACATC	TGTTAAGAAAAACTAGATTT
NFKBIA	CTGACGTTATGAGCGCAAAG	
NIFK	CTCAAACACCTACACATTCA	CTTTTTTTCCGTGAATGTGT
NOTCH1	GGGGCGCGCCGTTTACTTGA	TCCGGAGGCCTTCAAGTAAA
NR3C1	TAAGGCAACCATTCTTATTA	AGTGACTGCCTTAATAAGAA
NUMB	GTATCTTGTCCATACCAGAC	TGAACTTTAAGCAATCATTA
NUP98	GTTGGGAGCCTGTGAGCCCC	GCAAAGTGCCTGGGGCTCAC
PARP1	AAGACCTCCCTGTGGTAATT	CTCCCTGTGGTAATTGGGAG
PDGFRA	AGACAGCTTCCTGTAACTGG	GGAAGACAGCTTCCTGTAAC
PEX5	TGGCCTGCCCCAGTGACAGT	TGGCCTGCCCCAGTGACAGT
PLCG1	GGCTGGGGTACAACTAGAGG	CGAGGCTGGGGTACAACTAG
PPARG	GGAAATGTTGGCAGTGGCTC	GCTCCTGCAGGAGATCTACA
PRKAB1	ACAAGCCCATATGAAGAGCT	TCCGCCCCCAGCTCTTCATA
PRKCA	TCATACTGCACTCTGTAAGA	GGGGAGGTGTTTGTTCTCGC
PTEN	GTATGCTGATCTTCATCAAA	CTGAATTTTTTTTTATCAAG
PTK2	GACCACACTGAGCCTCCCCT	CTCCTAGGGGAGGCTCAGTG
PTPN11	TTCTGTGCTGAAGTTTTGGC	CTATTTCTGTGCTGAAGTTT
RAC1	GGGACAGGACCAAGAACGAG	GCTGAGACATTTACAACAGC
RB1	GGAAGAGAAATGAGGATCTC	TCAAACAAGGAAGAGAAATG
RHOA	TAAGGGCTGTGCTTGCAGCA	CAGCAAGGTTTCACAAGACA
RIPK1	CAGCCAGAACTAACCCTGGA	ACGTCAGCCAGAACTAACCC
RPS3	GCATAACAGGTATGTCTGCA	GCCAGTCCCCACAGCATAAC
SEC22B	TATTTCACAGCCACCAGAAT	GAAATAATGAATACAGTCAC
SERP2	GGGAGGTGGTGTCAAATCCC	ATGGGCATGTGAGAAAGCCA
SIRT2	CTGGGAGATGCAGCTGTCAC	CAGTGACAGCTGCATCTCCC
SMAD2	GTCTTTTCATGGGACTTGAT	GTTACATTAAGTCTTTTCAT
SMURF1	GAGCTAGACTCTGTTGCCTT	GTGGAGTGAAAAGCAACCAA
STAT3	CCCATGTGAGGAGCTGAGAA	TGCGCTACCTCCCCCATGTG
TNFRSF1A	GTCCTTAGAGCTGCCCGCAG	GCGCCCAGTCTTCTCAGATG
TNFSF10	GGTCAGTTAGCCAACTAAAA	TTTTAGTTGGCTAACTGACC
TOMM20	GAGCTTGGCTGAAGATGATG	AATTGTAAGTGCTCAGAGCT

TP53	TGTCAGTGGGGAACAAGAAG	GGAGAATGTCAGTCTGAGTC
TUBA1A	CTAAAGTTAAAACGTCACAA	AGAGGGTGAGGAAGAAGGAG
TUBG1	GATGAGGGTCCCTGTCCTGG	AGCAGTGAGTCCCCCAGGAC
VAMP1	AATGGACAACAGGGAAGGGG	GGCAATGGACAACAGGGAAG
VASP	CGGGGTTCTCCCTGACCACA	GCGGGGTTCTCCCTGACCAC
VCL	CCCTGGTACCAGTAGGCACC	AGTAGGCACCTGGCTGAGCC
VDR	GGCAATGAGATCTCCTGACT	CTCCTGACTAGGACAGCCTG
VHL	GCGCATTGCACATCAACGGA	CGCATTGCACATCAACGGAT

Supplementary Table S3. Donor DNA sequences. VS-HiBIT sequences are in green, stop codons are in red, and homology arms are in black. Point mutations are underlined.

Gene	Donor DNA
ACTB	AAGCAGGAGTATGACGAGTCCGGCCCCTCCATCGTCCACCGCAAATGCTTCGTCTCCGTGAGCGGCTGGCGGCTGTTCAAGAAGATTAGCTAGGCGGACTATGACTTAGTTGCGTTACACCCTTTCTTGACAAAACCTAA
AKT1	GAGCGCAGGCCCCACTTCCCCCAGTTCTCCTACTCGGCCAGCGGCACGGCCGTCTCCGTGAGCGGCTGGCGGCTGTTCAAGAAGATTAGCTGAGGCGGCGGTGGACTGCGCTCGACGATAGCTTGGAGGGATGGAGAGGC
APAF1	TATGTGACTGTGGATAACCTTGGTATTTTATATATTTTACAGACTTTAGAAGTCTCCGTGAGCGGCTGGCGGCTGTTCAAGAAGATTAGCTAAAATAGTTAAGCATTAATGTAGTTGAACTTTTTAAATTTTTGAATTGG
ARIH1	CAGCATGTGCATGAAGGCTATGAAAAAGATCTGTGGGAGTACATTGAGGACGTCTCCGTGAGCGGCTGGCGGCTGTTCAAGAAGATTAGCTGAGAATGGCGCTGCATAAAATGAACTCTGAAAACTTTACCATCTAGAGT
ARNT	TACAACAATGAAGAATTCCCTGATCTAACTATGTTTCCCCCCTTTTCAGAAGTCTCCGTGAGCGGCTGGCGGCTGTTCAAGAAGATTAGCTAGAACTATTGGGGTGAGGATAAGCCGTGGGGGAGAAAAAATCACTGTTT
ARRB2	CGGCTTCGGCTGAAGGGGATGAAGGATGACGACTATGATGATCAACTCTGCGTCTCCGTGAGCGGCTGGCGGCTGTTCAAGAAGATTAGCTAGGAAGCGGGGTGGGAAGAAGCGAGCGGATGGGGTTGGGAGAGGTGAGG
ATM	ATAGACCCCAAAAATCTCAGCCGACTTTTCCCAGGATGGAAAGCCTGGGTGGTCTCCGTGAGCGGCTGGCGGCTGTTCAAGAAGATTAGCTGATCTTCAGTATATGAATTACGCTTTCATTCAGCCTTTAGAAATTATAT
ATP1B3	GATCGTGACAAGTTTTTGGGACGAGTTATGTTCAAAATCACAGCACGTGCAGTCTCCGTGAGCGGCTGGCGGCTGTTCAAGAAGATTAGCTAGTATGAGTAGGATATCTCGACAGAGTAAATGTTGTGTTGTCTGTCTTC
AXL	CAGCCTGCTGATAGGGGCTCCCCAGCAGCCCCAGGGCAGGAGGATGGTGCCGTCTCCGTGAGCGGCTGGCGGCTGTTCAAGAAGATTAGCTGAGACAACCCTCGACCTGGTACTCCCTCTCAGGATCCAAGCTAAGCACT
BAD	CAGTCCTGGTGGGATCGGAACTTGGGCAGGGGAAGCTCCGCCCCCTCTCAGGTCTCCGTGAGCGGCTGGCGGCTGTTCAAGAAGATTAGCTGACCTTCGCTCGACATCCCGAAACTCCACCCGTTCCCACTGCCCTGGGC
BCL2	TTGGCCCTGGTGGGAGCTTGCATCACCCTGGGTGCCTATCTGGGTCACAAGGTCTCCGTGAGCGGCTGGCGGCTGTTCAAGAAGATTAGCTGAAGTCAACATGCCTGCGCCAAACAAATATGCAAAAGGTTCACTAAAGC
BRCA1	TGCCAGGAGCTGGACACCTACCTGATACCCCAGATCCCCCACAGCCACTACGTCTCCGTGAGCGGCTGGCGGCTGTTCAAGAAGATTAGCTGACTGCAGCCAGCCACAGGTACAGAGCCACAGGACCCCAAGAATGAGCT
CALR	AGGAGGAAGATGAGGAGGAAGATGTCCCCGGCCAGGCCAAGGACGAGCTGGTCTCCGTGAGCGGCTGGCGGCTGTTCAAGAAGATTAGCTAGAGAGGCCTGCCTCCAGGGCTGGACTGAGGCCTGAGCGCTCCTGCCGCAGA
CASP3	CAGATTCCATGTATTGTTTCCATGCTCACAAAAGAACTCTATTTTTATCACGTCTCCGTGAGCGGCTGGCGGCTGTTCAAGAAGATTAGCTAAAGAAATGGTTGGTTGGTGGTTTTTTTTAGTTTGTATGCCAAGTGAGA

CAT	ACCTTTGTGCAGTCCGGATCTCACTTGGCGGCAAGGGAGAAGGCAAATCTGGTCTCCGTGAGCGGCTGGCGGCTGTTCAAGAAGATTAGCTGAGGCCGGGGCGCTGCACCTGTGCAGCGAAGCTTAGCGTTCATCCGTGT
CAV1	GCTGTTGGGAAAATATTCAGCAATGTACGCATCAACTTGCAGAAAGAAATAGTCTCCGTGAGCGGCTGGCGGCTGTTCAAGAAGATTAGCTAAATGACATTTCAAGGATAGAAGTATACCTGATTTTTTTTCCTTTTAAT
CCND1	GAGGAGGTGGACCTGGCTTGCACACCCACCGACGTGCGGGACGTGGACATCGTCTCCGTGAGCGGCTGGCGGCTGTTCAAGAAGATTAGCTGAGGGCGCCAGGCAGGCGCGCGCCACCGCCACCCGCAGCGAGGGCGGAG
CENPA	AAGGATGTGCAACTGGCCCGGAGGATCCGGGGCCTTGAGGAGGCACTCGGCGTCTCCGTGAGCGGCTGGCGGCTGTTCAAGAAGATTAGCTGAGCTCCTGCACGCAGTGTTTCTGTCAGTCTTTCCTGCTCAGCCAGGGG
CFL1	GCAGAGAAGCTGGGGGGCAGTGCCGTCATCTCCCTGGAGGGCAAGCCTTTGGTCTCCGTGAGCGGCTGGCGGCTGTTCAAGAAGATTAGCTGAGCCGCTTCTGGCCCCCTGCCTGGAGCATCTGGCAGCCCCACACCTGC
CREBBP	CTGGTCGGGGACACCACGGGGGACACGCTAGAGAAGTTTGTAGAGGGCTTGGTCTCCGTGAGCGGCTGGCGGCTGTTCAAGAAGATTAGCTAGCATTGTGAGAGCATCACGTTTTCCCTTTCATGTTCTTGGACCTTTTG
CTNNB1	GGGCTGCCTCCAGGTGACAGCAATCAGCTGGCCTGGTTTGATACTGACCTGGTCTCCGTGAGCGGCTGGCGGCTGTTCAAGAAGATTAGCTAAATCATCGTTTAGGTAAGAAGTTTTAAAAAGCCAGTTTGGGTAAAATA
CUL1	GAATATTTGGAGCGAGTGGATGGTGAAAAGGACACCTACAGTTACTTGGCTGTCTCCGTGAGCGGCTGGCGGCTGTTCAAGAAGATTAGCTAACCGTTCTGGAAGGGTCTGACTGTGTGACCCGCAGCAAATAGTTCATG
CYCS	AAGGAAGAAAGGGCAGACTTAATAGCTTATCTCAAAAAAGCTACTAATGAGGTCTCCGTGAGCGGCTGGCGGCTGTTCAAGAAGATTAGCTAATAATTGGCGACTGCCTTATTTATTACAAAACAGAAATGTCTCATGAC
EEA1	TCCAAGAAGCCTGTTCGTGTCTGTGATGCATGTTTCAATGACTTGCAAGGAGTCTCCGTGAGCGGCTGGCGGCTGTTCAAGAAGATTAGCTAATGGGTTATCACAACTTCAGAGTAATATTACACTAACATTAGATTTTT
EPAS1	TCCACGCTCCTGCAAGGAGGGGACCTCCTCAGAGCCCTGGACCAGGCCACCGTCTCCGTGAGCGGCTGGCGGCTGTTCAAGAAGATTAGCTGAGCCAGGCCTTCTACCTGCGCAGCACCTCTGCCGACGCCGTCCCACCA
ESR2	TCATTTGGAATGAAGATGGAGACTCTTTTGCCTGAAGCAACGATGGAGCAGGTCTCCGTGAGCGGCTGGCGGCTGTTCAAGAAGATTAGCTGACCCTCTAATCAACTCCGTGGCCTAAAGAAAAATCTTGGGTAACATTT
EZH2	CAGGCTGATGCCCTGAAGTATGTCGGCATCGAAAGAGAAATGGAAATCCCTGTCTCCGTGAGCGGCTGGCGGCTGTTCAAGAAGATTAGCTGACATCTGCTACCTCCTCCCCCCTCCTCTGAAACAGCTGCCTTAGCTTC
EZR	CAGATCCGGCAGGGCAACACCAAGCAGCGCATCGACGAGTTCGAGGCCCTGGTCTCCGTGAGCGGCTGGCGGCTGTTCAAGAAGATTAGCTAACAGCCAGGCCAGGACCAAGCGCAGAGGGGTGCTCATAGCGGGCGCTG
F2R	GATACCTGCTCTAGTAACCTGAATAACAGCATATACAAAAAGCTGTTAACTGTCTCCGTGAGCGGCTGGCGGCTGTTCAAGAAGATTAGCTAGGAAAAGGGACTGCTGGGAGGTTAAAAAGAAAAGTTTATAAAAGTGAA

FGFR1	TGCCTGCCCCGACACCCAGCCCAGCTTGCCAATGGCGGACTCAAACGCCGCGTCTCCGTGAGCGGCTGGCGGCTGTTCAAGAAGATTAGCTGACTGCCACCGACACGCCCTCCCCAGACTCCACCGTCAGCTGTAACCCT
FOS	AGCAATGAGCCTTCCTCTGACTCGCTCAGCTCACCCACGCTGCTGGCCCTGGTCTCCGTGAGCGGCTGGCGGCTGTTCAAGAAGATTAGCTGAGGGGGCAGGGAAGGCGAGGCAGCCGGCACCCACAAGTGCCACTGCCC
FOXO3	GGGAACTTCACTGGTGCTAAGCAGGCCTCATCTCAGAGCTGGGTGCCAGGCGTCTCCGTGAGCGGCTGGCGGCTGTTCAAGAAGATTAGCTGAAGGATCACTGAGGAAGCGGAAGTGGGCAAAGCAGGTCAGTGCCGAAT
GABBR1	CCCGACCGGCTTAGCTGTGATGGGAGTCGAGTGCATTTGCTTTATAAGGTCTCCGTGAGCGGCTGGCGGCTGTTCAAGAAGATTAGCTGAGGGTAGGGTGAGGGAGGACAGGCCAGTAGGGGGAGGGAAAGGGAGAGGGG
GJA1	GCCAGCAGTCGTGCCAGCAGCAGACCTCGGCCTGATGACCTGGAGATCGTCTCCGTGAGCGGCTGGCGGCTGTTCAAGAAGATTAGCTAGATACAGGCTTGAAAGCATCAAGATTCCACTCAATTGTGGAGAAGAAAAAA
GOLGA2	TTTTTTTACCGGGCTGACGAGAATGATGAGGTGAAGATCACTGTCATCGTCTCCGTGAGCGGCTGGCGGCTGTTCAAGAAGATTAGCTAAAAGCGGGCTACTGTCAGCAAAGCCTGAAGAAGTGGGGCTGGATACCCTGC
GSK3B	ACCGTGGACAGACCAATAATGCTGCTTCTGCATCAGCTTCCAACTCCACCGTCTCCGTGAGCGGCTGGCGGCTGTTCAAGAAGATTAGCTGAACAGTCCCGAGCAGCCAGCTGCACAGGAAAAACCACCAGTTACTTGAGTG
H3F3A	ATGCCAAAAGACATCCAGCTAGCACGCCGCATACGTGGAGAACGTGCTGTCTCCGTGAGCGGCTGGCGGCTGTTCAAGAAGATTAGCTAAGAATCGACTATGATGGGAAACATTTCATTCTCAAAAAAAAAAAAAAAAAT
HDAC2	CACATTGCCTCTTTTAACAGAACCAAATCAGAACAGCTCAGCAACCCCGTCTCCGTGAGCGGCTGGCGGCTGTTCAAGAAGATTAGCTGAATTTGACAGTCTCACGAATTTCAGAAAATCATTAAAAAGAAAATATTGAA
HDAC6	AACATCGCCCACCAGAACAAGTTTGGGGAGGATATGCCCCACCCACACGTCTCCGTGAGCGGCTGGCGGCTGTTCAAGAAGATTAGCTAAGCCCCAGAATACGGTCCCTCTTCACCTTCTGAGGCCCACGATAGACCAGC
HIST1H4A	GTCTACGCGCTTAAGCGCCAGGGACGCACCCTTTATGGCTTTGGCGGTGTCTCCGTGAGCGGCTGGCGGCTGTTCAAGAAGATTAGCTAAGGTTGCTGATTTCTCGACAGCTTGCATTTCTGAACCAAAGGCCCTTTTCA
HSP90B1	GTTTTTTTTATTTATTTACAGGAATCTACAGCTGAAAAAGATGAATTGGTCTCCGTGAGCGGCTGGCGGCTGTTCAAGAAGATTAGCTAAATTATACTCTCACCATTTCGATCCTGTGTGGAGAGGGAATGTGAAATTTA
IL12A	GAATTCGGGCAGTGACTATTGATAGAGTGATGAGCTATCTGAATGCTTCCGTCTCCGTGAGCGGCTGGCGGCTGTTCAAGAAGATTAGCTAAAAAGCGAGGTCCCTCCAAACCGTTGTCATTTTTATAAAACTTTGAAATGA
IL15	AATTTTTGCAGAGTTTTGTACATATTGTGCAAATGTTCATCAACACTTCTGTCTCCGTGAGCGGCTGGCGGCTGTTCAAGAAGATTAGCTGATTGCAATTGATTCTTTTTAAAGTGTTTCTGTTATTAACAAACATCACTCT
IRAK4	TTCTTTCTTTTTAAAAGGTTCAACAGCTGCTGCAAGAGATGACAGCTTCTGTCTCCGTGAGCGGCTGGCGGCTGTTCAAGAAGATTAGCTAAAACTTTATTGGAAAAGACTCTTGACTTTTTATATACACCTATCTCAACCA

IRS1	TAAGCGCCTATGCCAGCATCAGTTTCCAGAAGCAGCCAGAGGACCGTCAGGTCTCCGTGAGCGGCTGGCGGCTGTTCAAGAAGATTAGCTAGCTCAACTGGACATCACAGCAGGTGCGTTTCATGGTGACAAAGTCAGAAGA
ITGB1	TTTATAAGAGTGCCGTAACAACTGTGGTCAATCCGAAGTACGAGGGAAAAGTCTCCGTGAGCGGCTGGCGGCTGTTCAAGAAGATTAGCTGAGTACTGCCCGTGCAAATCCCACAACACTGAATGCAAAGTA
JUN	ACGTTAACAGTGGGTGCCAACTCATGCTAACGCAGCAGTTGCAAACATTTGTCTCCGTGAGCGGCTGGCGGCTGTTCAAGAAGATTAGCTGAAGAGAGACCGTCGGGGGCTGAGGGGCAACGAAGAAAAAAATAACACAGA
KDM1A	TGCCTCGCCAGGCCACACCAGGTGTTCCTGCACAGCAGTCCCCAAGCATGGTCTCCGTGAGCGGCTGGCGGCTGTTCAAGAAGATTAGCTGAGACAGATGCATTCTAAGGGAAGAGGCCCATGTGCCTGTTTCTGCCATGTA
KLC1	TTGTGGAAAACGACAGCAGCAGCAGTGGCCTGGAAGACGCCACCGCTAACGGTCTCCGTGAGCGGCTGGCGGCTGTTCAAGAAGATTAGCTGAGTCCCACGGCCTGCAGCCCCAGGAAGCAGGCAGCTGGGCCAGGGAGGG
KPNB1	CCCTTGCTACATGGGCAACAAAAGAACTGAGGAAACTGAAGAACCAAGCTTGGGTCTCCGTGAGCGGCTGGCGGCTGTTCAAGAAGATTAGCTAAGATCTTGCCTCCACTGTCCTCTTTCTTCTACTTCCTGGAGGAGGGGG
KRAS	AAGATGGTAAAAAGAAGAAAAAGAAGTCAAAGACAAAGTGTGTAATTATGGTCTCCGTGAGCGGCTGGCGGCTGTTCAAGAAGATTAGCTAAATACAATTTGTACTTTTTTCTTAACGCATACTAGTACAAGTCGTAATTTT
LAMP1	TCGCCTACCTCGTCGGCAGGAAGAGGAGTCACGCAGGCTACCAGACTATCGTCTCCGTGAGCGGCTGGCGGCTGTTCAAGAAGATTAGCTAGCCTGGTGCACGCAGGCACAGCAGCTGCAGGGGCCTCTGTTCCTTTCTCTG
MAPK8	CTGTCTGCAACTGATTTGCTGTTTTGTTTCTCATAGCACAGGTGCAGCAGGTCTCCGTGAGCGGCTGGCGGCTGTTCAAGAAGATTAGCTGATCAATGGCTCTCAGCATTCATCATCATCGTCGTCTGTCAATGATGTGTCT
MARK3	CCATAGCCTTCAAAAATATTGCTTCCAAAATTGCCAATGAGCTAAAGCTGGTCTCCGTGAGCGGCTGGCGGCTGTTCAAGAAGATTAGCTAACTCAGTGATTATGATGTAAATTAAGTAGCAATTAAAGTGTTTTCCTGAA
MC1R	ACAGCCAGGAGCTCCGCAGGACGCTCAAGGAGGTGCTGACATGCTCCTGGGTCTCCGTGAGCGGCTGGCGGCTGTTCAAGAAGATTAGCTGAGCGCGGTGCACGCGGCTTTAAGTGTGCTGGGCAGAGGGAGGTGGTGATAT
MET	ACGCTGATGATGAGGTGGACACACGACCAGCCTCATTCTGGGAGACATCAGTCTCCGTGAGCGGCTGGCGGCTGTTCAAGAAGATTAGCTAGTGCTAGTACTATGTCAAAGCAACAGTCCACACTTTGTCCAATGGTTTTTT
MMP14	GGACCCCCAGGCGACTGCTCTACTGCCAGCGTTCCCTGCTGGACAAGGTCGTCTCCGTGAGCGGCTGGCGGCTGTTCAAGAAGATTAGCTGACGCCCACCGCCGGCCCGCCCACTCCTACCACAAGGACTTTGCCTCTGAAG
MMP14 (internal)	TGCCCCTCGTGTTTTCTGCCCCAGGGGGTGAGTCAGGGTTCCCCACCAAGATGCCCCCTCAACCCAGGACTGGCTCGAGCGGCGTGAGCGGCTGGCGGCTGTTCAAGAAGATTAGCGGCTCGAGCGGCACCTCCCGGCCTTCTGTTCCTGATAAACCCAAAAACCCCACCTATGGGCCCAACATCTGTGACGGGAACTTT
MTOR	AATTATTAAAATATTCCTTTTCTTTCTGCCTTCTTAGGTGCCCTTTCTGGGTCTCCGTGAGCGGCTGGCGGCTGTTCAAGAAGATTAGCTAACTGGAGGCCCAGATGTGCCCATCACGTTTTTTCTGAGGCTTTTGTACTTT
NFE2L2	GCAATGTTTTCCTTGTTCCCAAAAGTAAGAAGCCAGATGTTAAGAAAAACGTCTCCGTGAGCGGCTGGCGGCTGTTCAAGAAGATTAGCTAGATTTAGGAGGATTTGACCTTTTCTGAGCTAGTTTTTTTGTACTATTATAC

NFKBIA	TCCCCTTGTTTTCAGCTGCCCTATGATGACTGTGTGTTTGGAGGCCAGCGTCTGACGTTAGTGAGCGGCTGGCGGCTGTTCAAGAAGATTAGCTAATGAGCGCAAAGGGGCTGAAAGAACATGGACTTGTATATTTGTACAAAAAAAAAGTTTTAT
NIFK	CTCAAACACCTACTCATTCACGTAAAAAAAGACGAAGAAGCAGCAATCAGGTCTCCGTGAGCGGCTGGCGGCTGTTCAAGAAGATTAGCTGATTTTCAATGTATTATATTTCTTTTGAAAAATATAATATTTTTATGAGAGT
NOTCH1	CTCCCACCAGCATGCAGTCCCAGATCGCCCGCATTCCGGAGGCCTTCAAGGTCTCCGTGAGCGGCTGGCGGCTGTTCAAGAAGATTAGCTAAACGGCGCGCCCCACGAGACCCCGGCTTCCTTTCCCAAGCCTTCGGGCGTC
NR3C1	TACCAAAATATTCAAATGGAAATATCAAAAAACTTCTGTTTCATCAAAAGGTCTCCGTGAGCGGCTGGCGGCTGTTCAAGAAGATTAGCTGACTGCGTTATTAAGAATCGTTGCCTTAAAGAAAGTCGAATTAATAGCTTTT
NUMB	CTACCAACCCTTTCTCCAGTGACTTACAGAAGACGTTTGAAATTGAACTTGTCTCCGTGAGCGGCTGGCGGCTGTTCAAGAAGATTAGCTAAGCAATCATTATGGCTATGTATCTTGTCCATACCAGACAAGGAGCAGGGGG
NUP98	TGCGCAGCCTTACCCAGTCCTATCTGCGAGAACTGGCTGTTGGGAGCCTGGTCTCCGTGAGCGGCTGGCGGCTGTTCAAGAAGATTAGCTGAGCCCCAGGCACTTTGCATCACAGTCACATGCCCATTCACACCACACAGAG
PARP1	ATCTGAAGTATCTGCTGAAACTGAAATTCAATTTTAAGACCTCCCTGTGGGTCTCCGTGAGCGGCTGGCGGCTGTTCAAGAAGATTAGCTAATTGGGAGAGGTAGCCGAGTCACACCCGGTGGCTCTGGTATGAATTCACCC
PDGFRA	TGGATGACATCGGCATAGACTCTTCAGACCTGGTGGAAGACAGCTTCCTGGTCTCCGTGAGCGGCTGGCGGCTGTTCAAGAAGATTAGCTAACTGGCGGATTCGAGGGGTTCCTTCCACTTCTGGGGCCACCTCTGGATCCC
PEX5	CCGACGCGCGGGATCTGTCCACCCTCCTAACTATGTTTGGCCTGCCCCAGGTCTCCGTGAGCGGCTGGCGGCTGTTCAAGAAGATTAGCTGACAGTGGGACGGGCTGCCCTGTGAGTGTCCACCTGGAGGGATCCCCGCTTT
PLCG1	TTCTGTCCAGGGCCCCAAGAAGGACTCGGGTCAATGGAGACAACCGCCTCGTCTCCGTGAGCGGCTGGCGGCTGTTCAAGAAGATTAGCTAGTTGTACCCCAGCCTCGTTGGAGAGCAGCAGGTGCTGTGCGCCTTGTAGAA
PPARG	CAGACATGAGTCTTCACCCGCTCCTGCAGGAGATCTACAAAGACTTGTACGTCTCCGTGAGCGGCTGGCGGCTGTTCAAGAAGATTAGCTAGCAGAGAGTTCTGAGCCACTGCCAACATTTCCCTTCTTCCAGTTGCACTAT
PRKAB1	CCCACCGGTACAAGAAGAAGTACGTCACCACCTTGTTATACAAGCCCATAGTCTCCGTGAGCGGCTGGCGGCTGTTCAAGAAGATTAGCTGAAGAGCTGGGGGCGGATCGTGGCCCAGGAGACAGCACACCACCAGGCTCCA
PRKCA	TCTCGTATGTCAACCCCCAGTTTGTGCACCCCATCTTACAGAGTGCAGTAGTCTCCGTGAGCGGCTGGCGGCTGTTCAAGAAGATTAGCTGAAACTCACGAGCGAGAACAAACACCTCCCCAGCCCCCAGCCCTCCCCGCAG
PTEN	CAGAGAATGAACCTTTCGATGAAGATCAGCATACACAAATTACAAAAGTCGTCTCCGTGAGCGGCTGGCGGCTGTTCAAGAAGATTAGCTGAATTTTTTTTTATCAAGACGGATAAAACACCATGAAAATAAACTTGAATAA
PTK2	ATGTCATTGACCAAGCAAGACTGAAAATGCTTGGGCAGACGAGACCACACGTCTCCGTGAGCGGCTGGCGGCTGTTCAAGAAGATTAGCTGAGCCTCCCCTAGGAGCACGTCTTGCTACCCTCTTTTGAAGATGTTCTCTAG

PTPN11	CTAGAGTCTATGAAAACGTGGGCCTGATGCAACAGCAGAAAAGTTTCAGAGTCTCCGTGAGCGGCTG<br>GCGGCTGTTCAAGAAGATTAGCTGAGAAAACTTGCGAAAACTTCAGCACAGAAATAGGTATTTAAAT<br>GCAAGTGC
RAC1	TCCTCTGCCCGCCTCCCGTGAAGAAGAGGAAGAGAAAATGCCTGCTGTTGGTCTCCGTGAGCGGCTG<br>GCGGCTGTTCAAGAAGATTAGCTAAATGTCTCAGCGCCTCGTTCTTGGTCCTGTCCCTTGGAACCTTTG<br>TACGCT
RB1	AAAAGCAGAAAATGAATGATAGCATGGATACCTCAAACAAGGAAGAGAAAGTCTCCGTGAGCGGCT<br>GGCGGCTGTTCAAGAAGATTAGCTGAGGATCTCAGGACCTTGGTGGACACTGTGTACACCTCTGGAT<br>TCATTGTCT
RHOA	CTGCTCTGCAAGCTAGACGTGGGAAGAAAAAATCTGGGTGCCTTGTCTTGGTCTCCGTGAGCGGCTG<br>GCGGCTGTTCAAGAAGATTAGCTGAAATCTTGCTGCAAGCACAGCCCTTATGCGGTTAATTTTGAAGT<br>GCTGTTT
RIPK1	AGTGTTCCAGGATCGACCTTCTGAGCAGCTTGATTTACGTCAGCCAGAACGTCTCCGTGAGCGGCTGG<br>CGGCTGTTCAAGAAGATTAGCTAACCCTGGATGGGCTACGGCAGCTGAAGTGGACGCCTCACTTAGT<br>GGATAAC
RPS3	AGGGTGGGAAGCCAGAGCCGCCTGCCATGCCCCAGCCAGTCCCCACAGCAGTCTCCGTGAGCGGCTG<br>GCGGCTGTTCAAGAAGATTAGCTAACAGGTATGTCTGCAAGGGCAGGGGCCTCTTGGGGCATAATAG<br>GGTCCTTC
SEC22B	TAGCTGTATTTTTCATCATGTTAATAGTGTATGTCCGATTCTGGTGGCTGGTCTCCGTGAGCGGCTGGC<br>GGCTGTTCAAGAAGATTAGCTGAAATAATGAATACAGTCACTCGTAAGGGAGAACCTAGAACCCAGT<br>AGGTGTT
SERP2	TCCTCTTTTCAGCTATCTTTCAGATCATTCAGAGCATAAGGATGGGCATGGTCTCCGTGAGCGGCTGG<br>CGGCTGTTCAAGAAGATTAGCTGAGAAAGCGAGGGATTTGACACCACCTCCCTCCCACTGGAGG
SIRT2	GCCACCTGCCAAGGACGAGGCCAGGACAACAGAGAGGGAGAAACCCCAGGTCTCCGTGAGCGGCTG<br>GCGGCTGTTCAAGAAGATTAGCTGACAGCTGCATCTCCCAGGCGGGATGCCGAGCTCCTCAGGGACA<br>GCTGAGCC
SMAD2	AAGTATTAACTCAGATGGGATCCCCTTCAGTGCGTTGCTCAAGCATGTCAGTCTCCGTGAGCGGCTGG<br>CGGCTGTTCAAGAAGATTAGCTAAAGCTTCACAAATCAAGTTCCATGAAAAGACTTAATGTAACAACT<br>CTTCTG
SMURF1	ACGAGAAGCTGCTGACAGCCGTGGAGGAGACCTGCGGGTTTGCTGTGGAGGTCTCCGTGAGCGGCT<br>GGCGGCTGTTCAAGAAGATTAGCTGAAAAGCAACAAAAGGCAACAGAGTCTAGCTCATGGCCACCA<br>GACCAAAAGC
STAT3	CCCTCACCTTTGACATGGAGTTGACCTCGGAGTGCGCTACCTCCCCCATGGTCTCCGTGAGCGGCTGG<br>CGGCTGTTCAAGAAGATTAGCTGAGGAGCTGAGAACGGAAGCTGCAGAAAGATACGACTGAGGCGC<br>CTACCTGC
TNFRSF1A	AGGCGCTTTGCGGCCCCGCCGCCCTCCCGCCCGCGCCCAGTCTTCTCAGAGTCTCCGTGAGCGGCTGG<br>CGGCTGTTCAAGAAGATTAGCTGAGGCTGCGCACCTGCGGGCAGCTCTAAGGACCGTCCTGCGAGAT<br>CGCCTTC
TNFSF10	TGATAGACATGGACCATGAAGCCAGTTTTTTTGGGGCCTTTTTAGTTGGCGTCTCCGTGAGCGGCTGG<br>CGGCTGTTCAAGAAGATTAGCTAACTGACCTGGAAAGAAAAAGCAATAACCTCAAAGTGACTATTCA<br>GTTTTCA
TOMM20	TCTTTCAGAGAATTGTAAGTGCTCAGAGCTTGGCTGAAGATGATGTTGAAGTCTCCGTGAGCGGCTG<br>GCGGCTGTTCAAGAAGATTAGCTGAGAAACAAATGTCAACATAATAAAATCTCAGTTAAAAATATTTT<br>AAAAATT
TP53	CCTCCCGCCATAAAAAACTCATGTTCAAGACAGAAGGGCCTGACTCAGACGTCTCCGTGAGCGGCTG<br>GCGGCTGTTCAAGAAGATTAGCTGACATTCTCGACTTCTTGTTCCCCACTGACAGCCTCCCAC

TUBA1A	TTGGTGTGGATTCTGTTGAAGGAGAGGGTGAGGAAGAAGGAGAAGAATACGTCTCCGTGAGCGGCTGGCGGCTGTTCAAGAAGATTAGCTAAAGTTAAAACGTCACAAAGGTGCTGCTTTTACAGGGAAGCTTATTCTGTTT
TUBG1	ACCATGCGGCCACACGGCCAGACTACATCTCCTGGGGCACCCAGGAGCAGGTCTCCGTGAGCGGCTGGCGGCTGTTCAAGAAGATTAGCTGAGTACCCCAGGACAGGGACCCTCATCTGCCTTACTGGTTGGCCCAAGCCCT
VAMP1	CTTTTCCGTCTGGGACTTCCTGATTCCTGTGTCCAGTCTACTTTTTTACTGTCTCCGTGAGCGGCTGGCGGCTGTTCAAGAAGATTAGCTGAGAATGTACCACGCCTTCCCTGTTGTCCATTGCCATCCACATTCATGTCCT
VASP	CTTGTTTCCTTCCAGCCTTCGTCCAGGAGCTGAGGAAGCGGGGTTCTCCCGTCTCCGTGAGCGGCTGGCGGCTGTTCAAGAAGATTAGCTGACCACAGGGACCCAGAAGACCCGCTTCTCCTTTCCGCACACCCGGCCTGTC
VCL	CAGATGCTGGATTTACACTGCGCTGGGTTAGAAAGACTCCCTGGTACCAGGTCTCCGTGAGCGGCTGGCGGCTGTTCAAGAAGATTAGCTAGGCACCTGGCTGAGCCTGGCTGGCACAGAAACCTCTACTAAAAAGAAGGAA
VDR	GCATGAAGCTAACGCCCCTTGTGCTCGAAGTGTTTGGCAATGAGATCTCCGTCTCCGTGAGCGGCTGGCGGCTGTTCAAGAAGATTAGCTGACTAGGACAGCCTGTGGCGGTGCCTGGGTGGGGCTGCTCCTCCAGGGCCAC
VHL	ACCTGGAGCGGCTGACACAGGAGCGCATTGCACATCAACGTATGGGAGATGTCTCCGTGAGCGGCTGGCGGCTGTTCAAGAAGATTAGCTGAAGATTTCTGTTGAAACTTACACTGTTTCATCTCAGCTTTTGATGGTACTG

Supplementary Table S4. Average RLU, standard deviation, and SBR for each target and guide in HeLa cells.

Gene	Guide	Average RLU	STDEV	SBR
ACTB	1	98914	47364	130.3
	2	15909425	3152778	21123.6
AKT1	1	4822	140	5.4
	2	2845	238	2.8
APAF1	1	1106	19	0.5
ARIH1	1	14628	83	18.4
	2	8717	159	10.6
ARNT	1	2598	115	2.4
	2	1994	210	1.6
ARRB2	1	542	31	-0.3
	2	743	52	0.0
ATM	1	1004	13	0.3
	2	1096	119	0.5
ATP1B3	1	5132	72	5.8
	2	12378	158	15.4
AXL	1	15124	146	19.1
	2	84767	33287	111.6
BAD	1	4227	837	4.6
	2	1363	108	0.8
BCL2	1	910	49	0.2
	2	1073	54	0.4
BRCA1	1	1179	262	0.6
	2	2247	158	2.0
CALR	1	329065	5737	435.9
	2	373329	49531	494.7
CASP3	1	32403	1076	42.0
	2	37321	3989	48.6
CAT	1	2688	534	2.6
	2	4522	394	5.0
CAV1	1	261604	47395	346.4
	2	6884	905	8.1
CCND1	1	1555	115	1.1
	2	1193	91	0.6
CENPA	1	1509	33	1.0
	2	2481	132	2.3
CFL1	1	3406911	251124	4522.7
	2	3539263	20764	4698.5

CREBBP1	1	1028	15	0.4
	2	1741	127	1.3
CTNNB1	1	20474	1144	26.2
	2	25989	1623	33.5
CUL1	1	16357	3314	20.7
	2	90258	6229	118.8
CYCS	1	72953	13341	95.9
	2	228100	204	301.9
EEA1	1	48944	632	64.0
	2	46712	940	61.0
EPAS1	1	907	14	0.2
	2	762	64	0.0
ESR2	1	754	32	0.0
	2	899	0	0.2
EZH2	1	706	45	-0.1
	2	5691	95	6.6
EZR	1	374516	72866	496.3
	2	540214	24503	716.3
F2R	1	840	55	0.1
	2	1203	112	0.6
FGFR1	1	714	19	-0.1
	2	651	68	-0.1
FOS	1	794	48	0.1
	2	983	47	0.3
FOXO3	1	960	43	0.3
	2	1388	88	0.8
GABBR1	1	772	45	0.0
	2	769	59	0.0
GJA1	1	180420	2347	238.6
	2	62221	2569	81.6
GOLGA2	1	658	55	-0.1
	2	785	67	0.0
GSK3B	1	81238	3258	106.9
	2	115405	5321	152.2
H3F3	1	320091	48867	424.0
	2	110653	31355	145.9
HDAC2	1	153471	14266	202.8
	2	1319	521	0.8
HDAC6	1	54195	1164	71.0
	2	33594	150	43.6
HIST1H4A	1	64846	7904	85.1

	2	16047	519	20.3
HSP90B1	1	1555003	18405	2063.7
	2	1798695	34408	2387.3
IL12A	1	793	14	0.1
	2	759	37	0.0
IL15	1	829	32	0.1
IRAK4	1	7213	915	8.6
IRS1	1	5814	1347	6.7
	2	3904	493	4.2
ITGB1	1	174622	28815	230.9
	2	133647	6577	176.5
JUN	1	2409	399	2.2
	2	4952	654	5.6
KDM1A	1	46782	5235	61.1
	2	25943	716	33.4
KLC1	1	10455	236	12.9
	2	5963	150	6.9
KPNB1	1	797127	9499	1057.4
	2	562440	45829	745.8
KRAS	1	979	79	0.3
	2	1044	133	0.4
LAMP1	1	2020	706	1.7
	2	249080	16727	329.7
MAPK8	1	951	79	0.3
	2	1866	84	1.5
MARK3	1	891	16	0.2
	2	894	48	0.2
MC1R	1	693	27	-0.1
	2	700	7	-0.1
MET	1	12228	398	15.2
	2	5200	126	5.9
MMP14	1	804	41	0.1
	2	803	21	0.1
MTOR	1	712	47	-0.1
	2	722	22	0.0
NFE2L2	1	980	82	0.3
	2	2962	149	2.9
NFKBIA	1	6806	143	8.0
NIFK	1	10434	775	12.9
	2	4806	181	5.4
NOTCH1	1	1305	2	0.7

	2	2940	93	2.9
NR3C1	1	6653	636	7.8
	2	4889	781	5.5
NUMB	1	1578	78	1.1
	2	43504	2593	56.8
NUP98	1	2614	91	2.5
	2	68688	531	90.2
PARP1	1	485716	49166	643.9
	2	1555	231	1.1
PDGFRA	1	1127	0	0.5
	2	852	51	0.1
PEX5	1	3947	60	4.2
	2	17999	1798	22.9
PLCG1	1	205180	3676	271.4
	2	60312	1548	79.1
PPARG	1	865	22	0.1
	2	894	0	0.2
PRKAB1	1	29320	788	37.9
	2	12816	155	16.0
PRKCA	1	20858	616	26.7
	2	40700	1352	53.0
PTEN	1	1394	39	0.9
	2	926	61	0.2
PTK2	1	21713	647	27.8
	2	26142	3386	33.7
PTPN11	1	2921	176	2.9
	2	27424	753	35.4
RAC1	1	66211	7133	86.9
	2	131884	22381	174.1
RB1	1	8226	731	9.9
	2	9427	335	11.5
RHOA	1	22981	2580	29.5
	2	77023	6600	101.3
RIPK1	1	4270	316	4.7
	2	5758	608	6.6
RPS3	1	100070	2489	131.9
	2	985695	82324	1307.8
SEC22B	1	1293	30	0.7
	2	1081	39	0.4
SERP2	1	747	1	0.0
	2	741	18	0.0

SIRT2	1	23133	795	29.7
	2	14641	919	18.4
SMAD2	1	34491	2801	44.8
	2	37104	1258	48.3
SMURF1	1	1168	130	0.6
	2	2356	300	2.1
STAT3	1	34243	852	44.5
	2	28383	3882	36.7
TNFRSF1A	1	2340	157	2.1
	2	7437	380	8.9
TNFSF10	1	974	1	0.3
	2	948	41	0.3
TOMM20	1	62968	6116	82.6
	2	13639	2026	17.1
TP53	1	1164	57	0.5
	2	1295	24	0.7
TUBA1A	1	18178	2220	23.1
	2	105215	137924	138.7
TUBG1	1	33387	181	43.3
	2	20817	535	26.6
VAMP	1	813	5	0.1
	2	858	20	0.1
VASP	1	78276	3353	102.9
	2	49406	3625	64.6
VCL	1	2355283	13637	3126.4
	2	519820	3574	689.2
VDR	1	1417	160	0.9
	2	762	64	0.0
VHL	1	1807	184	1.4
	2	7217	527	8.6
Unedited HeLa	NA	753	156	NA

Supplementary Table S5. Average RLU, standard deviation, and SBR for each target and guide in K-562 cells.

Gene	Guide	Average RLU	STDEV	SBR
ACTB	1	19816769	1950429	36589.2
	2	51543312	10893553	95170.0
AKT1	1	106840	3753	196.3
	2	73210	9727	134.2
APAF1	1	366	6	-0.3
ARIH1	1	87170	1434	160.0
	2	24454	1873	44.2
ARNT	1	25864	704	46.8
	2	15913	785	28.4
ARRB2	1	740	48	0.4
	2	1442	238	1.7
ATM	1	584	23	0.1
	2	407	15	-0.2
ATP1B3	1	795702	31310	1468.2
	2	1241592	13434	2291.5
AXL	1	2339	508	3.3
	2	30357	1641	55.1
BAD	1	8983	1413	15.6
	2	2427	394	3.5
BCL2	1	668	177	0.2
	2	531	130	0.0
BRCA1	1	19713	551	35.4
	2	34500	1480	62.7
CALR	1	30598839	426077	56497.5
	2	44712880	1071151	82558.1
CASP3	1	220670	2453	406.5
	2	215549	5795	397.0
CAT	1	18796	5248	33.7
	2	244924	49213	451.2
CAV1	1	1572	104	1.9
	2	347	22	-0.4
CCND1	1	724	6	0.3
	2	439	31	-0.2
CENPA	1	23332	1428	42.1
	2	22670	1570	40.9
CFL1	1	9343114	246176	17250.4
	2	13660878	452776	25222.8

CREBBP1	1	1086	63	1.0
	2	16638	2506	29.7
CTNNB1	1	11946	1572	21.1
	2	6497	525	11.0
CUL1	1	625884	48269	1154.6
	2	900451	132292	1661.6
CYCS	1	4589212	239576	8472.6
	2	3838295	143063	7086.1
EEA1	1	323387	86488	596.1
	2	209142	10718	385.2
EPAS1	1	591	20	0.1
	2	476	92	-0.1
EPAS1 (+Phenanthroline)	1	4059	796	6.5
ESR2	1	1003	48	0.9
	2	949	89	0.8
EZH2	1	615	20	0.1
	2	252807	2583	465.8
EZR	1	2182469	267634	4028.8
	2	2858680	776138	5277.3
F2R	1	1719	57	2.2
	2	2613	122	3.8
FGFR1	1	529	147	0.0
	2	573	17	0.1
FOS	1	486	27	-0.1
	2	533	26	0.0
FOS (+PMA)	2	102416	14179	188.1
FOXO3	1	3226	235	5.0
	2	8753	1108	15.2
GABBR1	1	362	33	-0.3
	2	387	16	-0.3
GJA1	1	25527	1520	46.1
	2	12943	1949	22.9
GOLGA2	1	2542	115	3.7
	2	1343	51	1.5
GSK3B	1	274368	26152	505.6
	2	453320	43273	836.0
H3F3	1	12852586	391696	23730.4
	2	13043441	2502966	24082.8
HDAC2	1	1101251	168171	2032.4
	2	2259	442	3.2
HDAC6	1	806510	128019	1488.2

	2	1104777	119924	2038.9
HIST1H4A	1	553442	62602	1020.9
	2	358110	17809	660.2
HSP90B1	1	27061460	746070	49966.0
	2	29505423	1407996	54478.6
IL12A	1	944	214	0.7
	2	1035	78	0.9
IL15	1	342	10	-0.4
IRAK4	1	147277	13146	270.9
IRS1	1	11515	1336	20.3
	2	7792	1297	13.4
ITGB1	1	384181	39579	708.4
	2	517471	24794	954.5
JUN	1	30779	926	55.8
	2	12268	1016	21.7
KDM1A	1	432092	50267	796.8
	2	1037633	175902	1914.9
KLC1	1	217762	9727	401.1
	2	54111	3366	98.9
KPNB1	1	7123558	574193	13152.1
	2	7986699	711984	14745.9
KRAS	1	4185	46	6.7
	2	608	33	0.1
LAMP1	1	1290	90	1.4
	2	505980	39044	933.3
MAPK8	1	54094	2571	98.9
	2	47286	8375	86.3
MARK3	1	1193	125	1.2
	2	15044	730	26.8
MC1R	1	374	14	-0.3
	2	474	53	-0.1
MET	1	366	22	-0.3
	2	371	30	-0.3
MMP14	1	401	132	-0.3
	2	309	41	-0.4
MTOR	1	1371	8	1.5
	2	2393	179	3.4
NFE2L2	1	413	11	-0.2
	2	544	79	0.0
NFKBIA	1	3716	99	5.9
NIFK	1	139318	5867	256.2

	2	47267	4341	86.3
NOTCH1	1	18200	1461	32.6
	2	82129	11643	150.6
NR3C1	1	19970	1038	35.9
	2	29367	955	53.2
NUMB	1	13190	306	23.4
	2	141555	20336	260.4
NUP98	1	21249	2059	38.2
	2	923006	281079	1703.3
PARP1	1	2379765	128661	4393.1
	2	10528	2180	18.4
PDGFRA	1	350	9	-0.4
	2	359	12	-0.3
PEX5	1	7431	747	12.7
	2	140405	2432	258.2
PLCG1	1	1344633	38204	2481.8
	2	463386	13559	854.6
PPARG	1	549	43	0.0
	2	446	36	-0.2
PRKAB1	1	245973	48076	453.2
	2	13737	1330	24.4
PRKCA	1	24669	553	44.5
	2	441	37	-0.2
PTEN	1	1279	302	1.4
	2	488	31	-0.1
PTK2	1	509022	88186	938.9
	2	301829	9233	556.3
PTPN11	1	36609	5034	66.6
	2	634709	28033	1170.9
RAC1	1	239683	17288	441.6
	2	269310	8786	496.3
RB1	1	421047	14254	776.4
	2	558318	42497	1029.9
RHOA	1	232603	48334	428.5
	2	414066	25049	763.5
RIPK1	1	303169	61929	558.8
	2	450610	32119	831.0
RPS3	1	2352039	50616	4341.9
	2	8214568	38471	15166.6
SEC22B	1	1868	123	2.4
	2	1895	223	2.5

SERP2	1	346	14	-0.4
	2	376	42	-0.3
SIRT2	1	364084	44960	671.3
	2	14548	1111	25.9
SMAD2	1	182107	29879	335.2
	2	137589	43219	253.0
SMURF1	1	1518	124	1.8
	2	5500	1171	9.2
STAT3	1	956432	33959	1765.0
	2	354000	84165	652.6
TNFRSF1A	1	31319	8799	56.8
	2	38004	13767	69.2
TNFSF10	1	540	35	0.0
	2	368	48	-0.3
TOMM20	1	467705	89835	862.6
	2	146754	18055	270.0
TP53	1	501	49	-0.1
	2	404	39	-0.3
TUBA1A	1	434797	37963	801.8
	2	6581615	3834224	12151.5
TUBG1	1	362016	68132	667.4
	2	32261	486	58.6
VAMP	1	1509	37	1.8
	2	2619	54	3.8
VASP	1	1310878	66508	2419.4
	2	213710	14642	393.6
VCL	1	2797556	144286	5164.5
	2	856667	23367	1580.8
VDR	1	1648	22	2.0
	2	371	13	-0.3
VHL	1	2253	159	3.2
	2	151050	15518	277.9
Unedited K-562	NA	542	176	NA

Supplementary Table S6. Guide, RLU, and SBR for each target in Jurkat cells.

Gene	Guide	RLU	SBR
ACTB	2	2300249	917.6
AKT1	1	103422	40.3
APAF1	1	2518	0.0
ARIH1	1	15956	5.4
ARNT	1	15930	5.4
ARRB2	2	2272	-0.1
ATM	1	2088	-0.2
ATP1B3	2	183000	72.1
AXL	2	7605	2.0
BAD	1	77942	30.1
BCL2	1	3801	0.5
BRCA1	2	9148	2.7
CALR	2	46948651	18748.5
CASP3	1	444765	176.6
CAT	2	31495	11.6
CAV1	1	2447	0.0
CCND1	1	2321	-0.1
CENPA	1	68970	26.5
CFL1	2	6811325	2719.2
CREBBP	2	8766	2.5
CTNNB1	1	14280	4.7
CUL1	2	204364	80.6
CYCS	1	454557	180.5
EEA1	1	156650	61.6
EPAS1	1	2595	0.0
ESR2	1	2527	0.0
EZH2	2	65382	25.1
EZR	2	2258044	900.8
F2R	2	3392	0.4
FGFR1	2	2415	0.0
FOS	1	2809	0.1
FOXO3	2	2850	0.1
GABBR1	2	3026	0.2
GJA1	1	2441	0.0
GOLGA2	1	2354	-0.1
GSK3B	2	725804	288.9
H3F3A	1	494141	196.3
HDAC2	1	1362783	543.2

HDAC6	2	106640	41.6
HIST1H4A	1	704993	280.5
HSP90B1	2	4197297	1675.2
IL12A	2	2552	0.0
IL15	1	2465	0.0
IRAK4	1	75705	29.2
IRS1	2	10202	3.1
ITGB1	1	28095	10.2
JUN	1	10021	3.0
KDM1A	2	192572	75.9
KLC1	1	103554	40.4
KPNB1	2	3539859	1412.7
KRAS	1	3655	0.5
LAMP1	2	899845	358.4
MAPK8	1	5130	1.0
MARK3	2	6202	1.5
MC1R	2	2496	0.0
MET	2	3062	0.2
MMP14	1	2867	0.1
MTOR	2	2774	0.1
NFE2L2	2	3955	0.6
NFKBIA	1	50329	16.1
NIFK	1	3027	0.2
NOTCH1	2	20103	7.0
NR3C1	2	17599	6.0
NUMB	2	13363	4.3
NUP98	2	332672	106.4
PARP1	1	824735	328.4
PDGFRA	1	2818	0.1
PEX5	2	15395	5.1
PLCG1	1	1566136	624.5
PPARG	1	2802	0.1
PRKAB1	1	84861	32.9
PRKCA	1	119156	46.6
PTEN	1	2799	0.1
PTK2	1	63973	24.5
PTPN11	2	36112	13.4
RAC1	2	95642	37.2
RB1	2	1707963	681.1
RHOA	2	240197	94.9
RIPK1	2	38766	14.5

RPS3	2	2071420	826.2
SEC22B	2	3031	0.2
SERP2	2	3016	0.2
SIRT2	1	142790	56.0
SMAD2	1	265004	104.8
SMURF1	2	3930	0.6
STAT3	1	117107	45.8
TNFRSF1A	2	16376	5.5
TNFSF10	1	3084	0.2
TOMM20	1	119672	46.8
TP53	1	2871	0.1
TUBA1A	2	18249	6.3
TUBG1	1	309467	122.6
VAMP	2	3084	0.2
VASP	1	224836	88.8
VCL	1	239961	94.8
VDR	1	2836	0.1
VHL	2	58619	22.4
Unedited Jurkat	NA	2504	NA

Supplementary Figure S1. Reproducibility of editing experiments. MAPK8, JUN, RIPK1, CAT, NR3C1, CTNNB1, TOMM20, RHOA, CUL1, H3F3A, LAMP1, ITGB1, HDAC2, PARP1, and ACTB (listed from lowest to highest signal) were independently targeted for C-terminal HiBiT integration with two different passages of HeLa cells. Plot shows correlation of SBR obtained from each editing experiment for the selected targets.

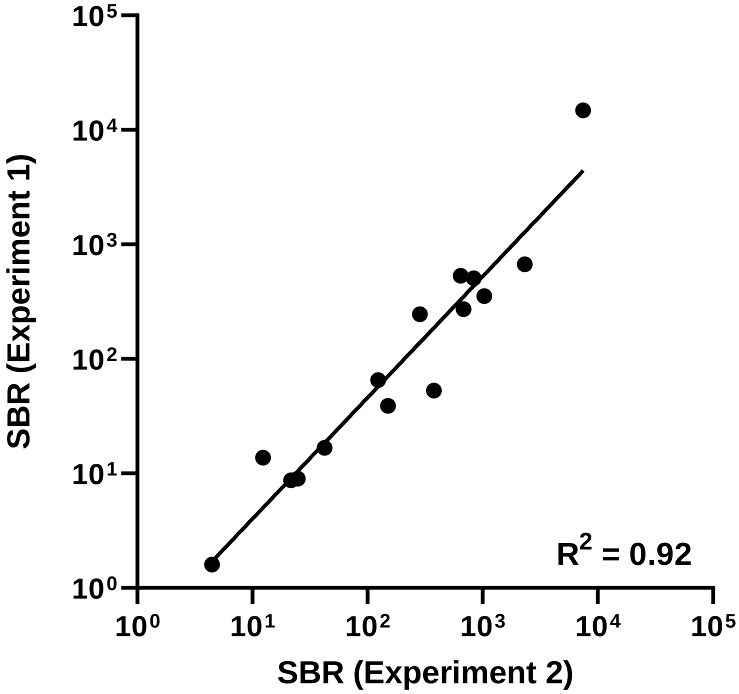

Supplemental Table S7. Reported normalized expression (NX) and calculated SBR for targets that were not detected in either one of the two cell lines, HeLa or K-562, or both of the cell lines. Yellow highlighting indicates no expression (NX <1). Blue highlighting indicates no HiBiT signal (SBR <1).

Target	NX (HeLa)	NX (K-562)	SBR (HeLa)	SBR (K-562)
APAF1	6.3	5.6	0.5	0.0
ARRB2	9.9	2.7	0.0	1.7
ATM	4.7	8.2	0.5	0.1
BCL2	0.6	0.2	0.4	0.2
CCND1	4.7	0.0	1.1	0.3
EPAS1	1.8	0.5	0.2	6.5
ESR2	0.7	0.6	0.2	0.9
F2R	0.7	4.2	0.6	3.8
FGFR1	1.5	2.2	0.0	0.1
FOS	3.8	0.4	0.3	188.1
FOXO3	2.9	9.7	0.8	15.2
GABBR1	3.4	6.6	0.0	0.0
GOLGA2	34.0	23.8	0.0	1.5
IL12A	0.1	0.0	0.1	0.9
IL15	13.7	3.8	0.1	0.0
KRAS	7.9	11.2	0.4	6.7
MARK3	17.1	21.0	0.2	26.8
MC1R	3.0	1.8	0.0	0.0
MET	27.1	0.9	15.2	0.0
MMP14	0.1	0.1	0.1	0.0
MTOR	28.6	25.1	0.0	1.5
NFE2L2	22.3	14.5	2.9	0.0
PPARG	1.9	0.0	0.2	0.0
PTEN	12.8	3.1	0.9	1.4
SEC22B	18.6	4.6	0.7	2.5
SERP2	0.0	0.0	0.0	0.0
TNFSF10	0.4	0.0	0.3	0.0
TP53	20.0	0.9	0.7	0.0
VAMP1	10.2	0.5	0.1	3.8
VDR	1.3	0.7	0.9	2.0

Supplementary Figure S2. Distribution of relative luminescence signal across a panel of targets in primary T cells. Luminescence measurements were obtained in lytic mode and normalized to total cell number. Colors increase from white to dark blue, with white representing targets with SBR less than one and shades of blue representing targets with SBR greater than or equal to one.

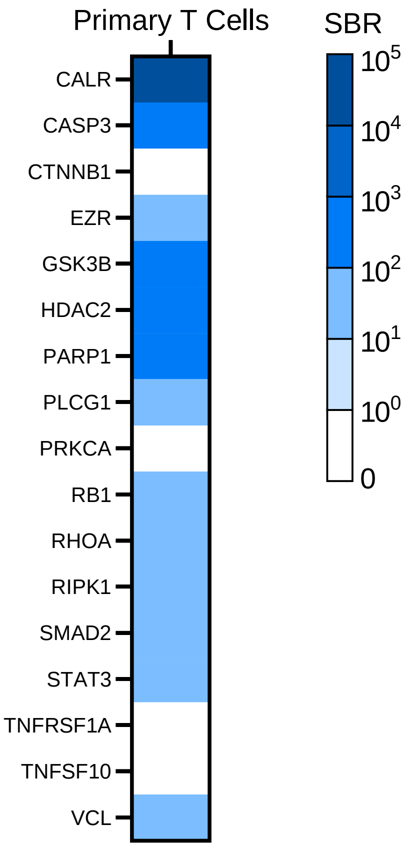

Supplementary Figure S3. Detection of HiBiT tag in FOS and EPAS1 edited K-562 cell pools. (a) FOS-edited cells were incubated in the presence or absence of 30 nM PMA for 4 h prior to luminescence measurement. (b) EPAS1 cells were incubated in the presence or absence of 20 µM phenanthroline for 6 h. Data are represented as mean luminescence values normalized to cell number as measured in lytic format with variability expressed as SD (n = 3). SBR for each condition is represented in parentheses. Dashed line indicates luminescence produced by unedited K-562 cells

a

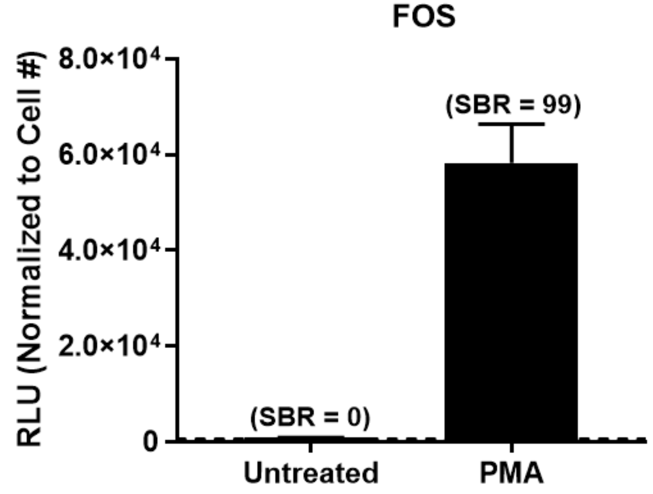

b

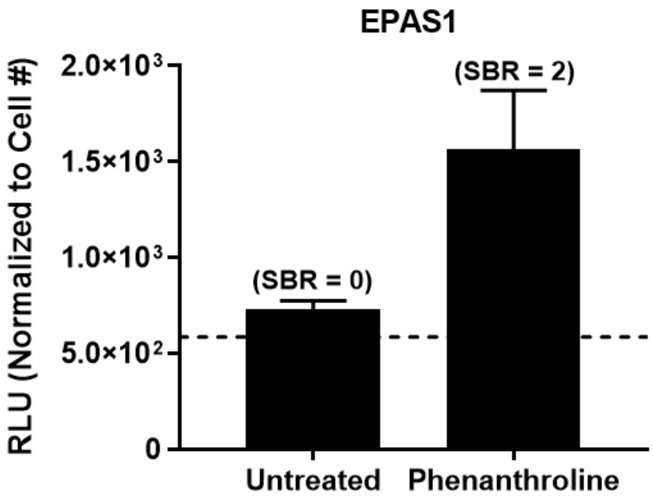

Supplementary Figure S4. Luminescence output of endogenously tagged MMP14 in U-2 OS cells. Integration of HiBiT was directed to the C-terminus using 2 different guides (Guide 1 and Guide2) and internally at Thr300 using one guide (Guide 3). Data are represented as mean luminescence values normalized to cell number (n = 3) as measured in lytic format with variability expressed as SD. SBR for each condition is represented in parentheses. Dashed line indicates luminescence produced by unedited U-2 OS cells.

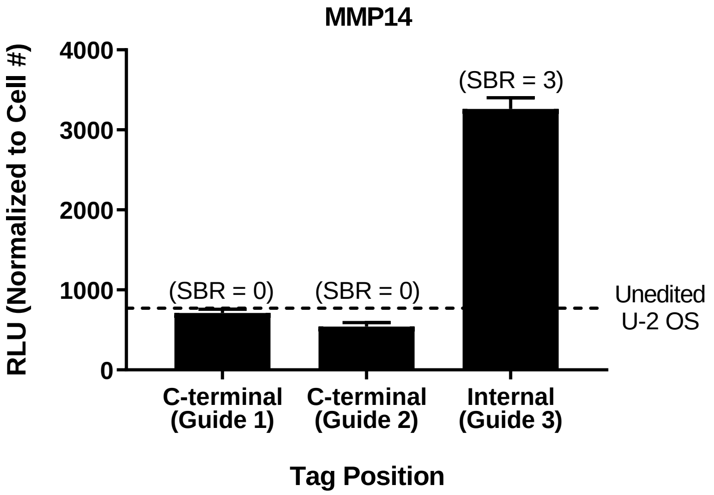

Supplementary Figure S5. Represented are sections of different HiBiT blots for the indicated protein fusion with expected apparent molecular weights in parentheses. Values to the left of each blot represent the migration of the corresponding size standard. Blots were cropped for each target and analyzed using Fiji with linear contrast adjustments to view each protein band. Uncropped blots are available in Fig. S6a-j.

a

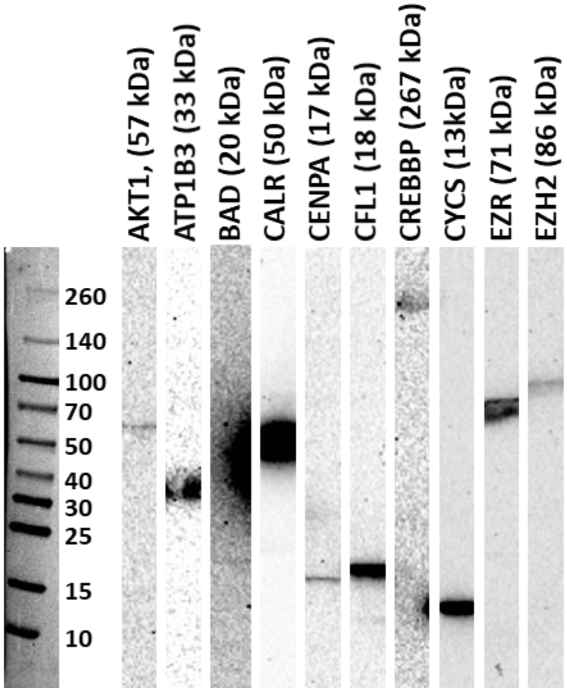

b

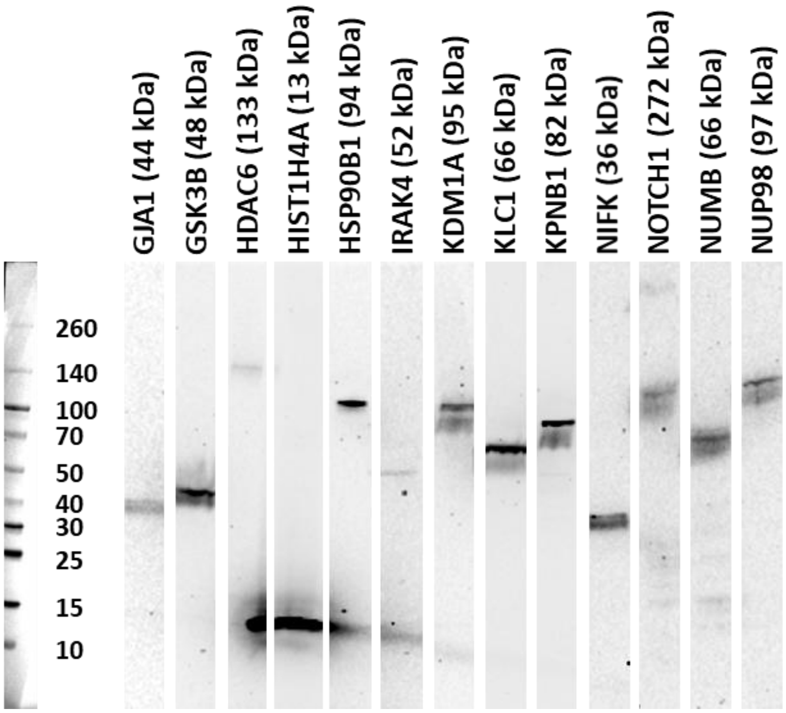

Supplementary Figure S5 (continued).

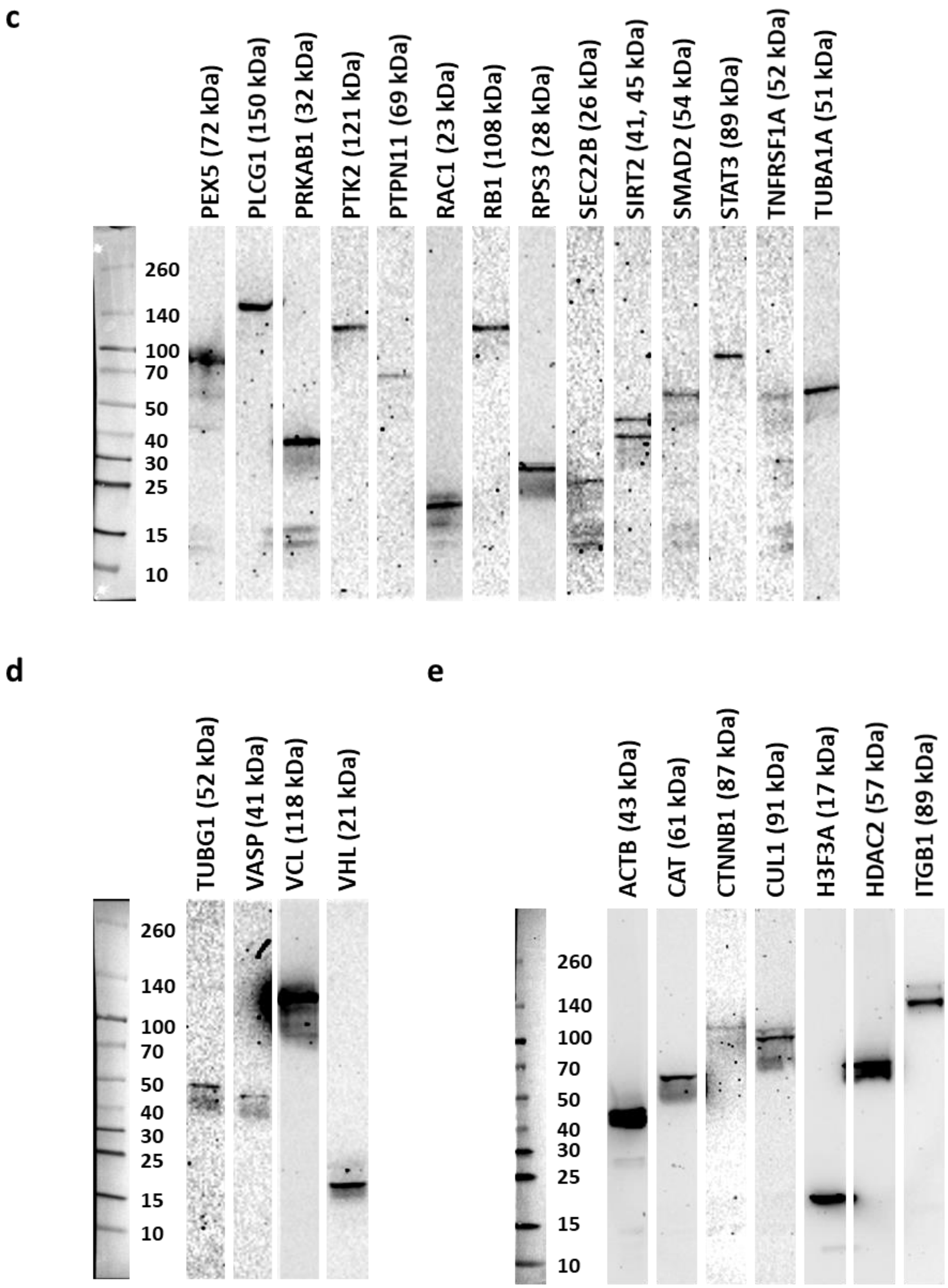

Supplementary Figure S5 (continued).

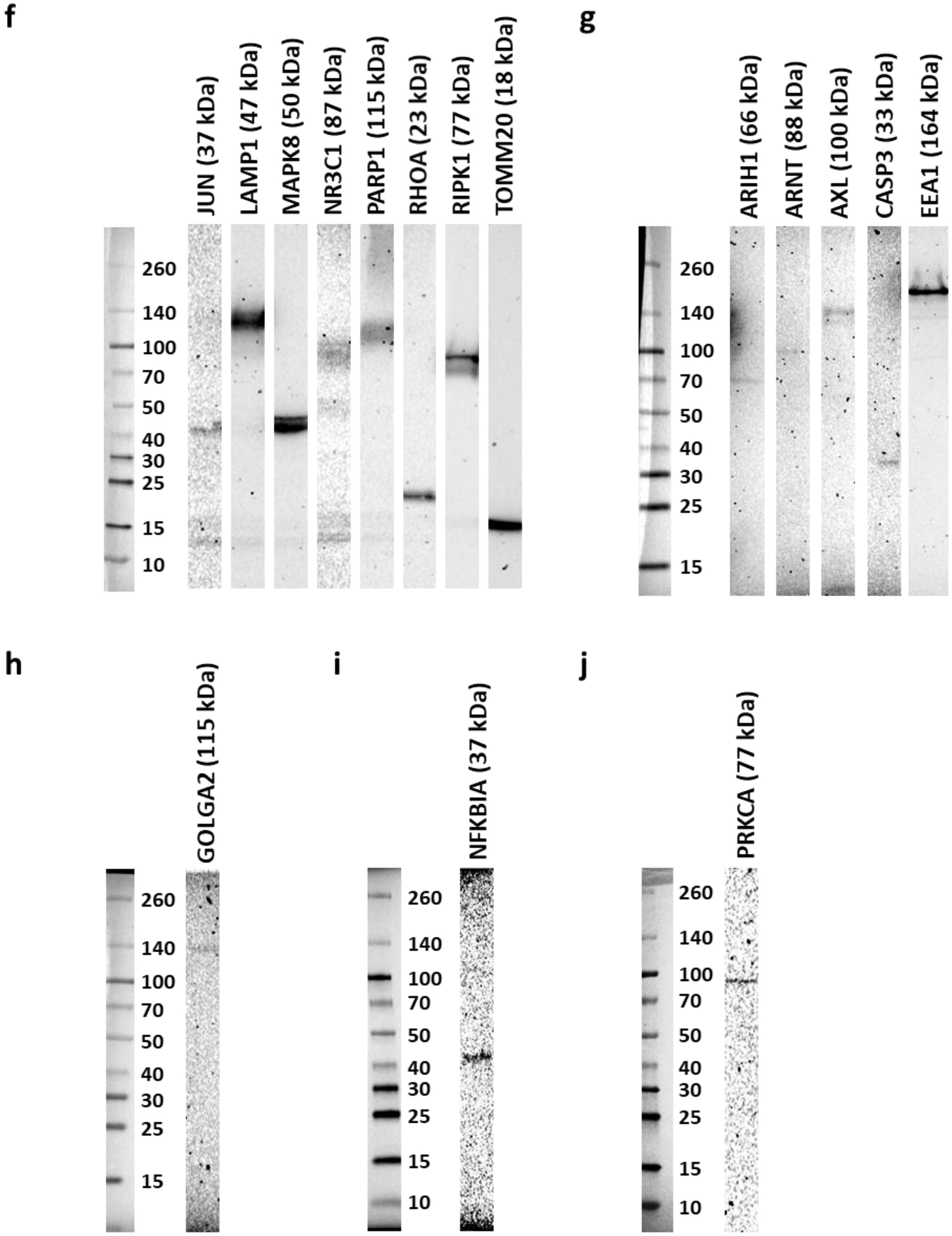

Supplementary Figure S6. Full-size unedited HiBiT blots shown in in Fig. 3 and Fig. S5a-j. Left panels show colorimetric images of membranes with visible molecular weight ladder. Right panels show luminescence images of the same membranes. Luminescence images were collected using BioRad Image Lab 5.2.1 software with exposures of 5 min (e, g), 10 min (b, f), 15 min (a, c, d), and 60 min (h, i, j). Red boxes indicate where blots were cropped to generate figures.

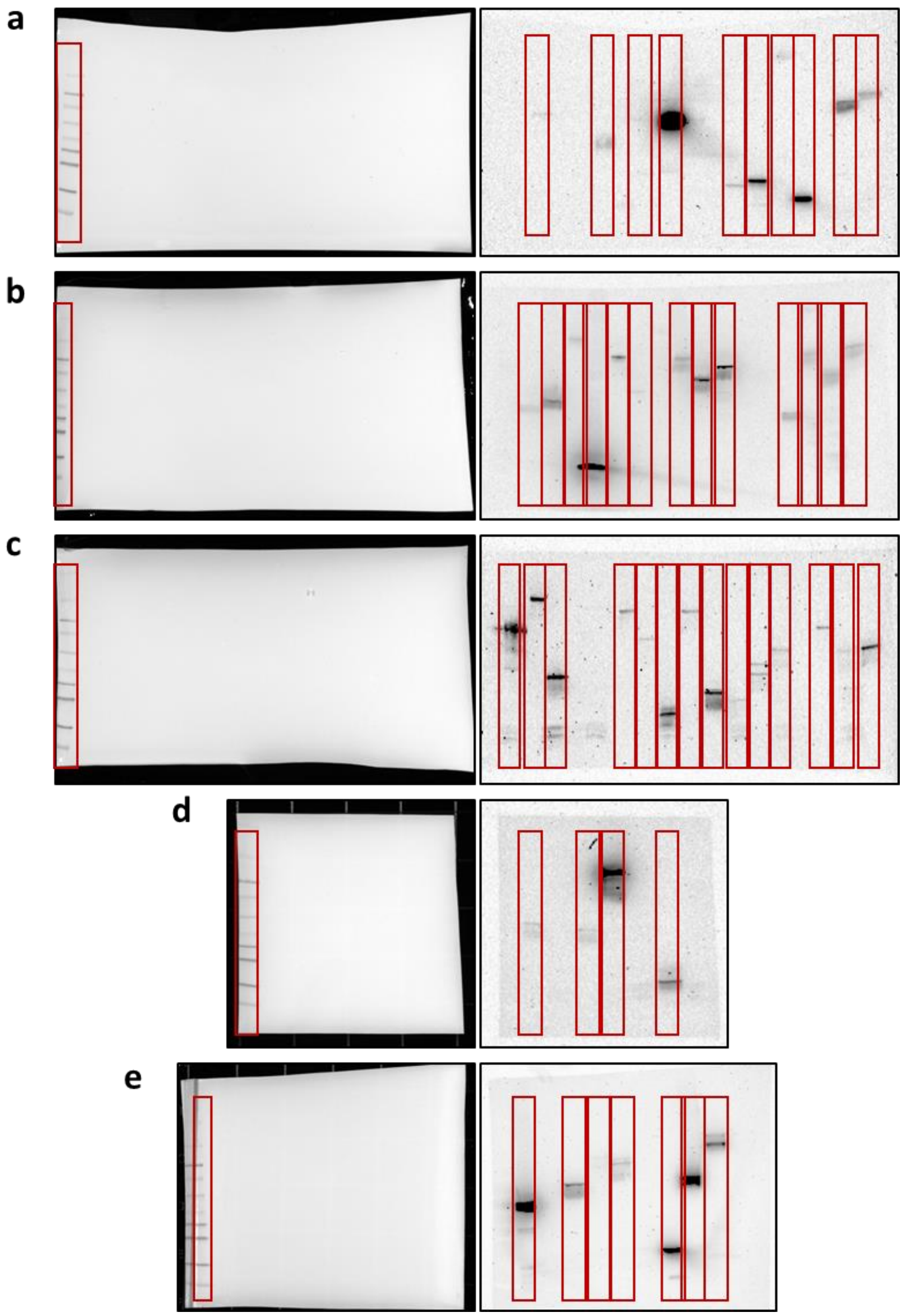

Supplementary Figure S6 (continued).

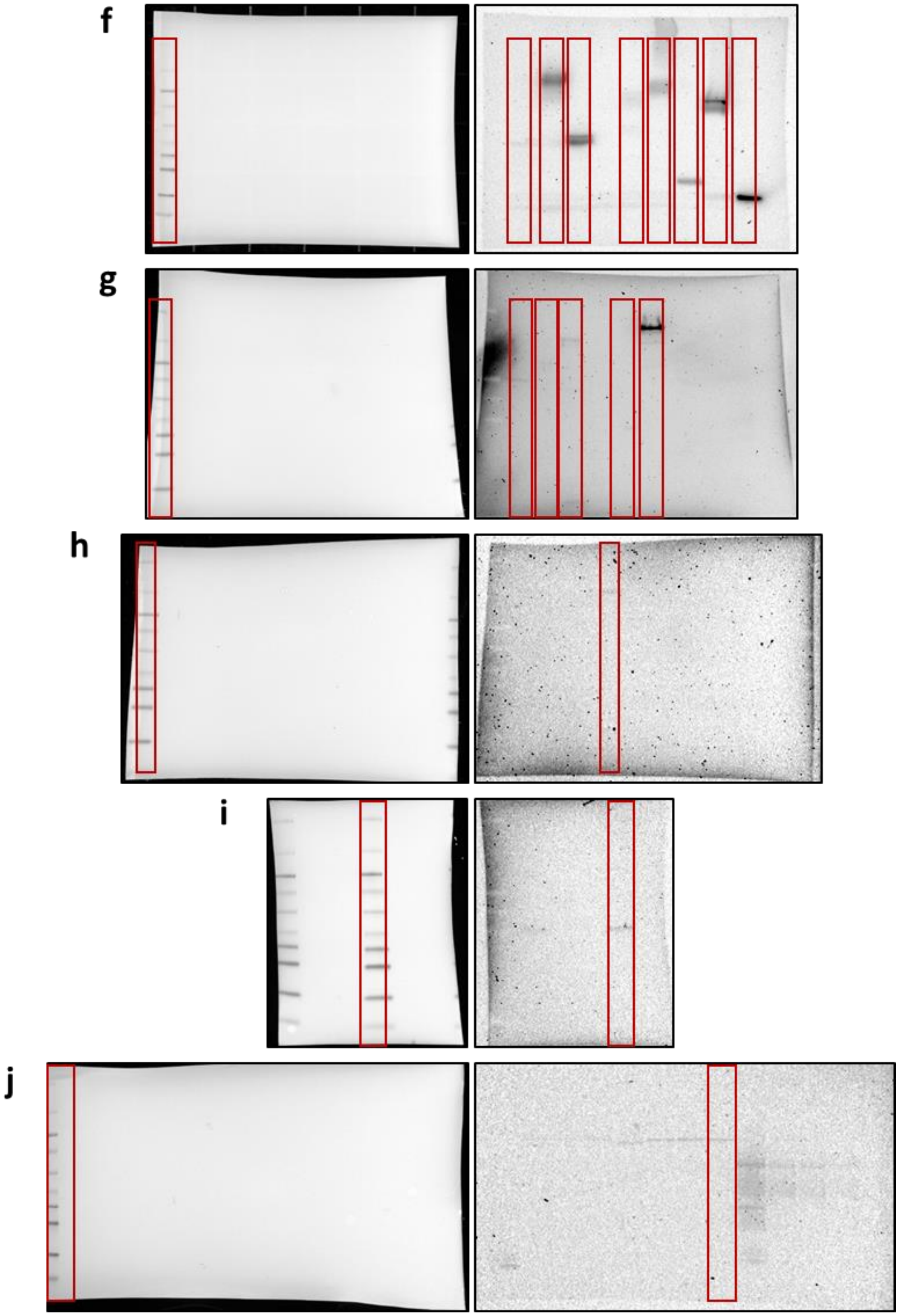

Supplementary Figure S7. Bioluminescence imaging of HiBiT tagged targets in HeLa cells. Top panels show pseudo-colored bioluminescence images of HiBiT fusions in edited HeLa pools. Bottom panels are an overlay of the bioluminescence image with the corresponding brightfield image. Objectives, EM, and exposure times for each image are found in Table S8. Scale bar = 20 µm.

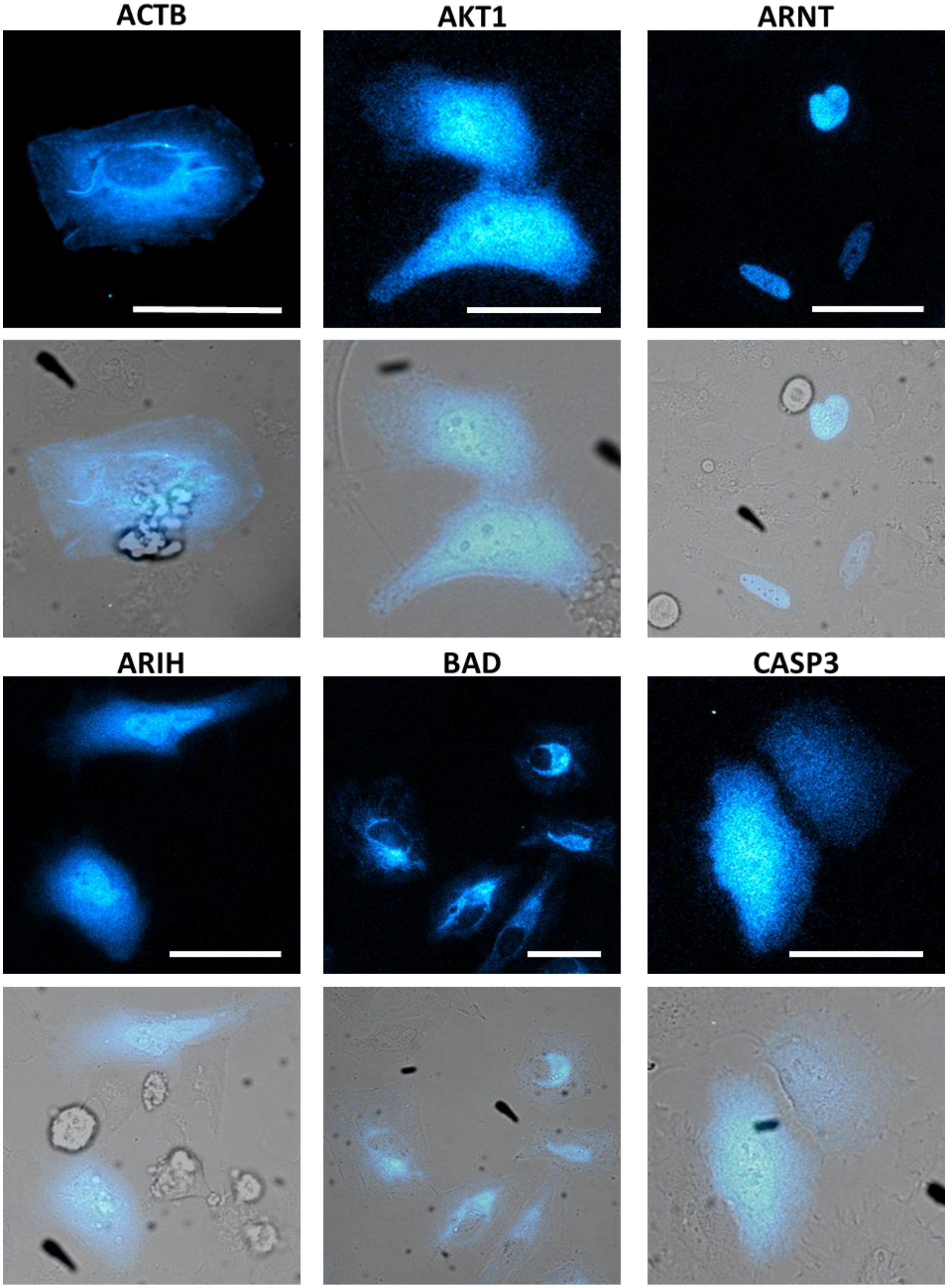

Supplementary Figure S7 (continued).

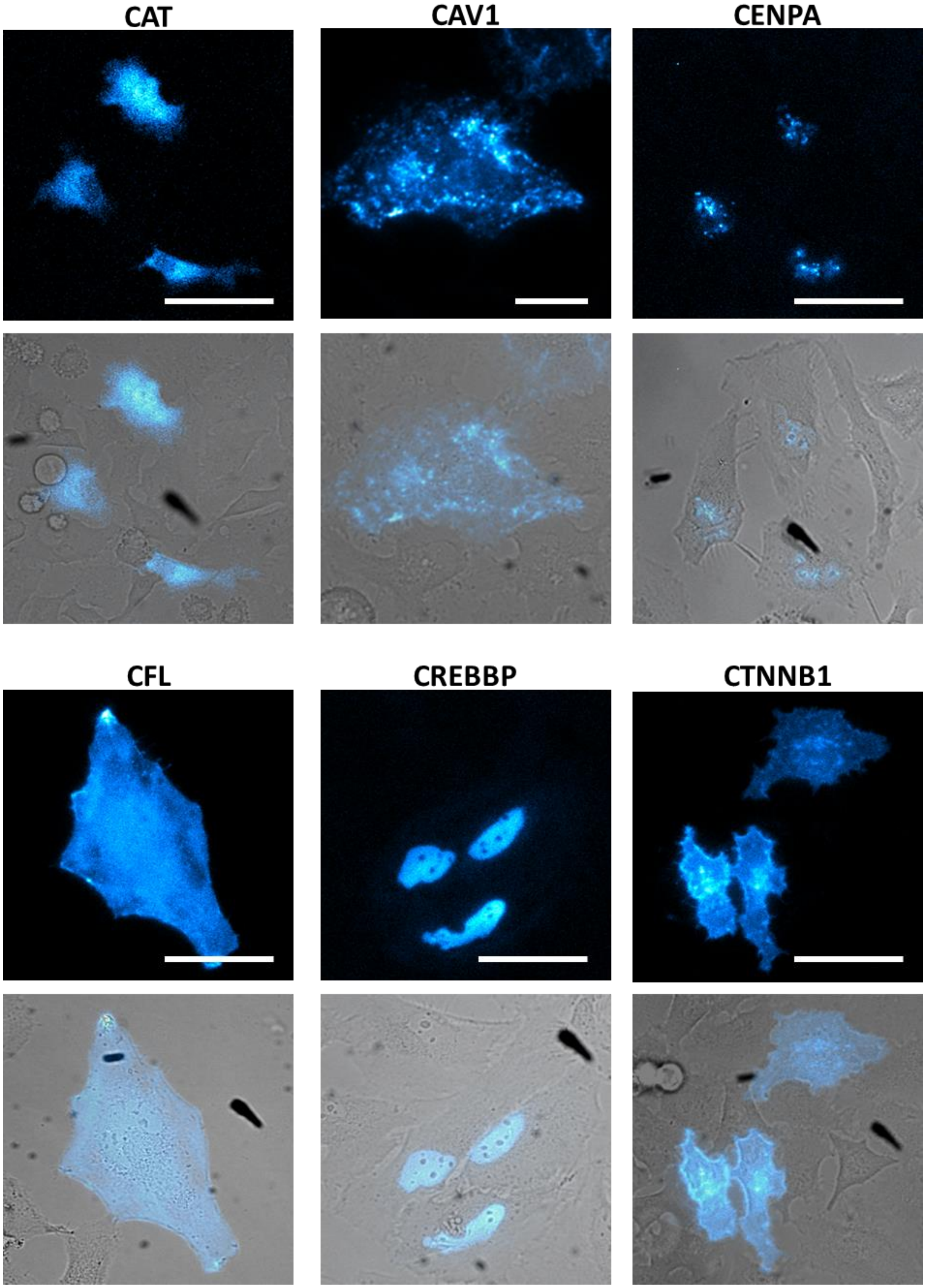

Supplementary Figure S7 (continued).

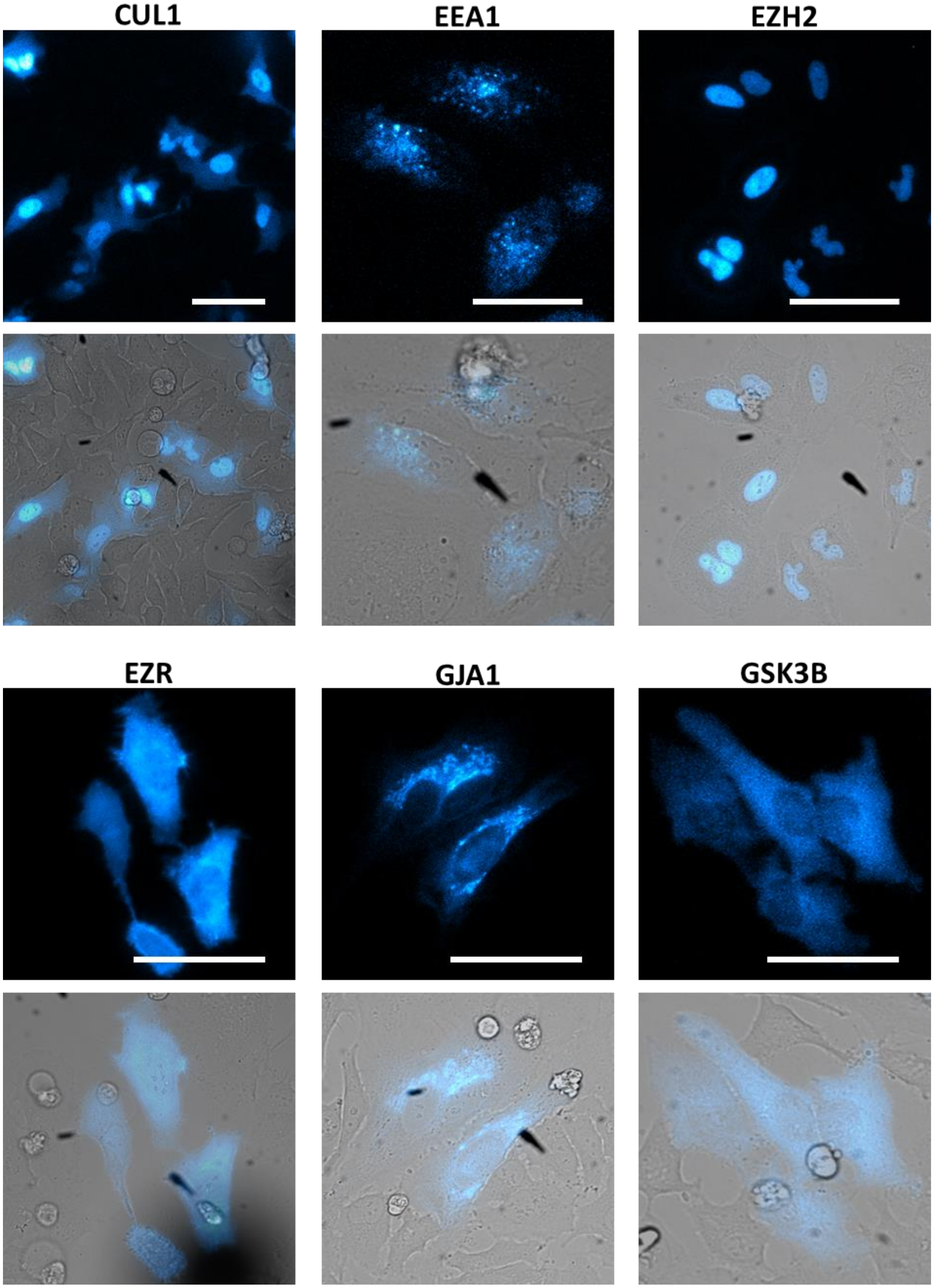

Supplementary Figure S7 (continued).

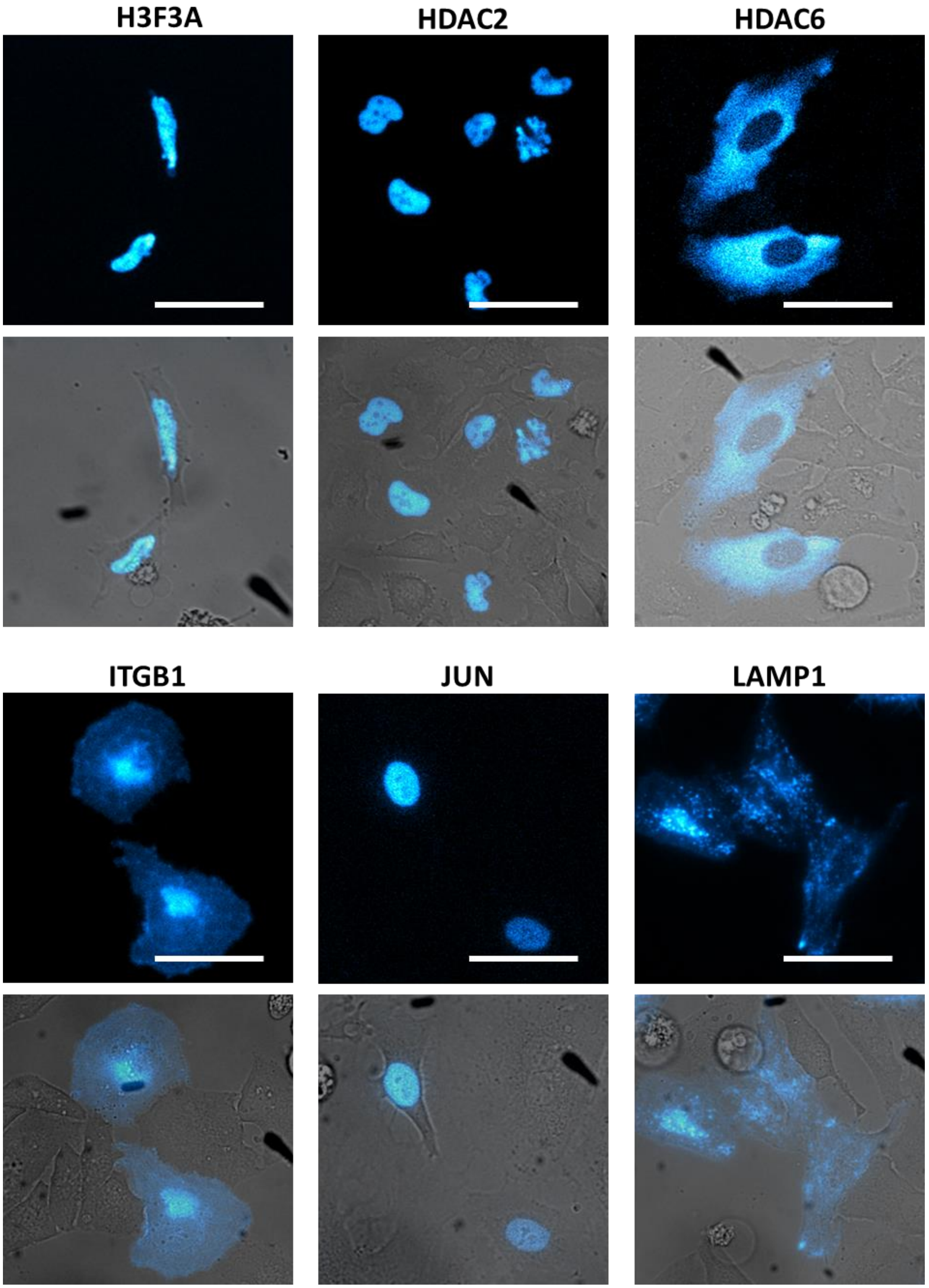

Supplementary Figure S7 (continued).

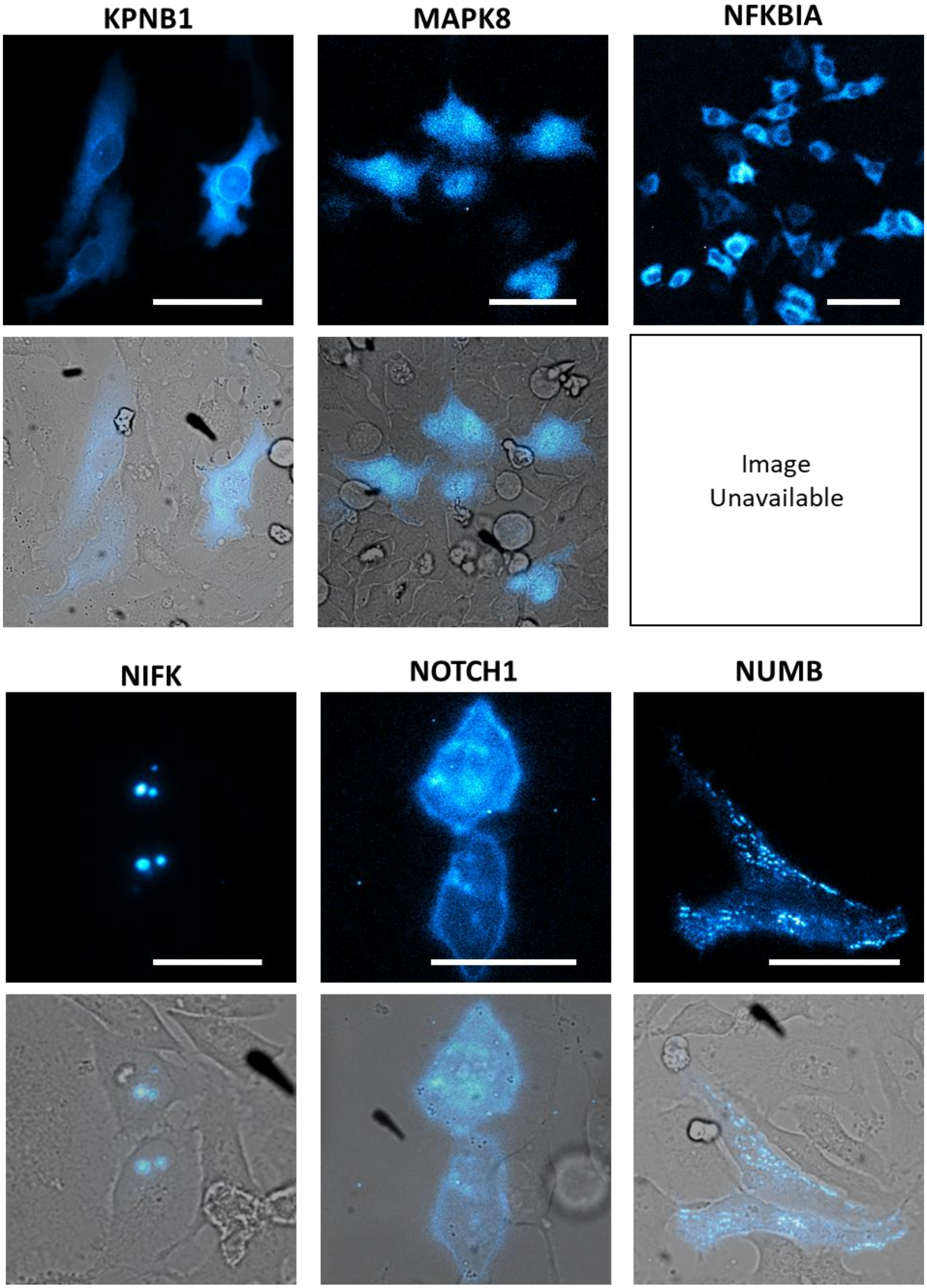

Supplementary Figure S7 (continued).

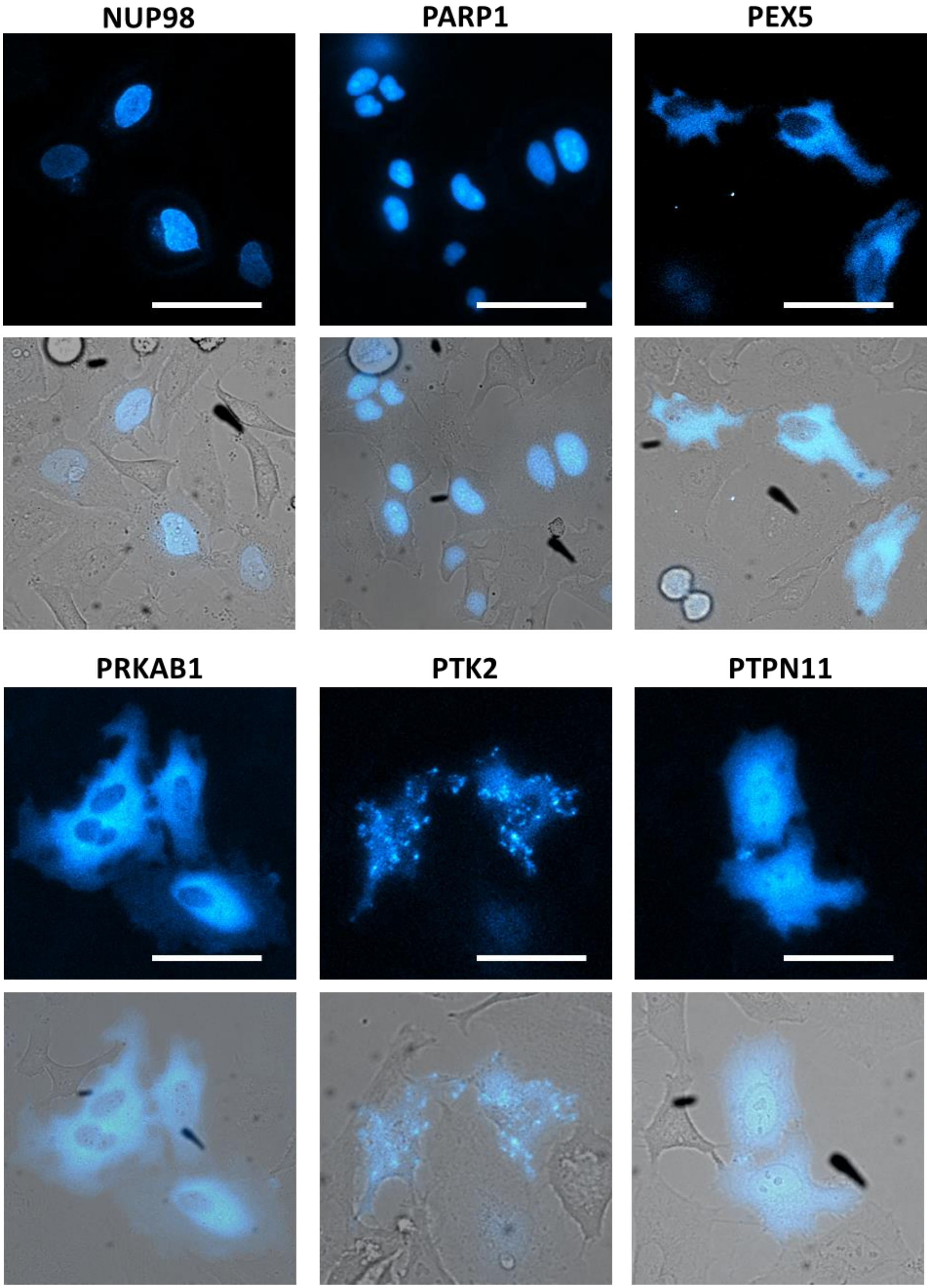

Supplementary Figure S7 (continued).

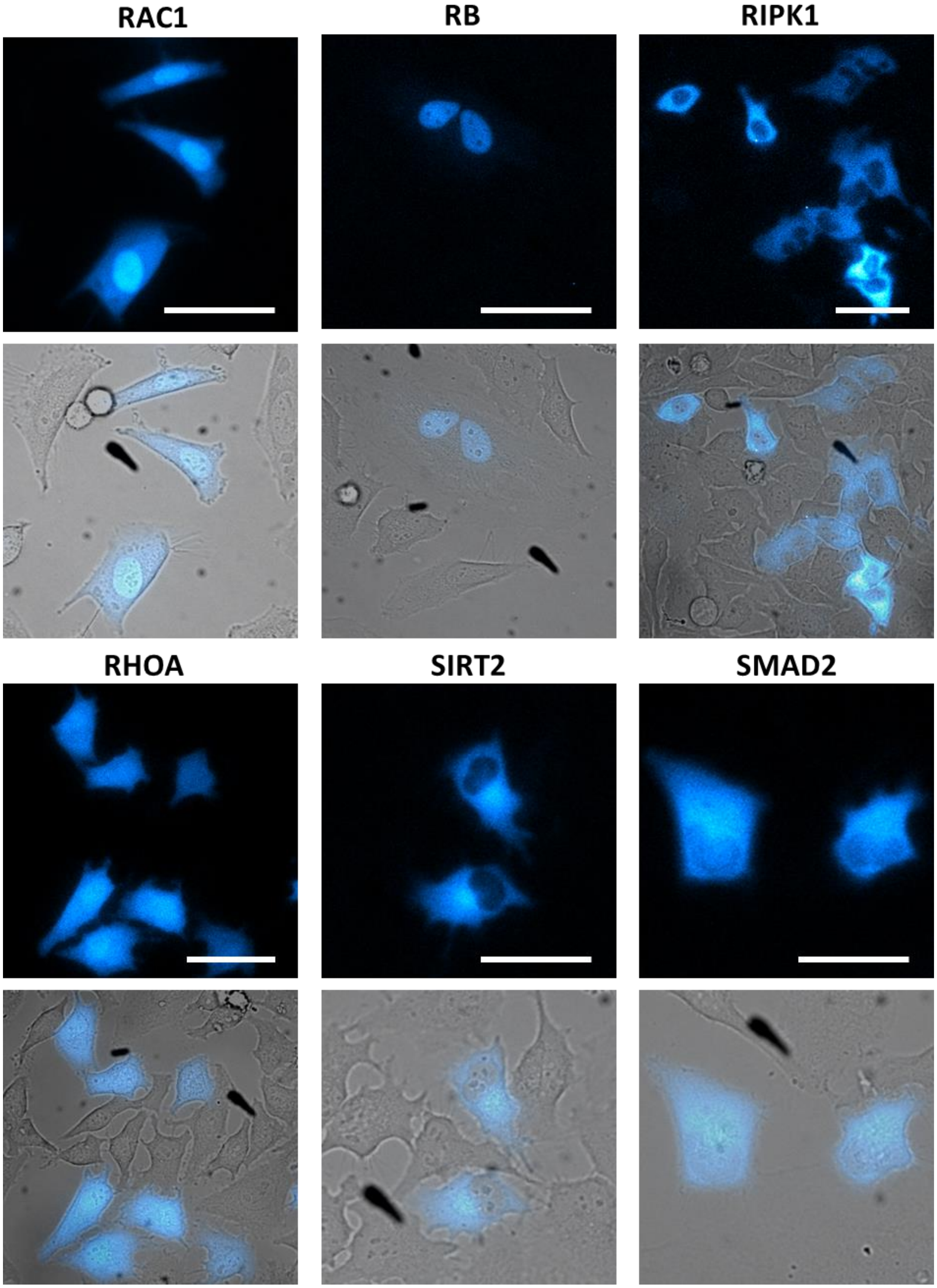

Supplementary Figure S7 (continued).

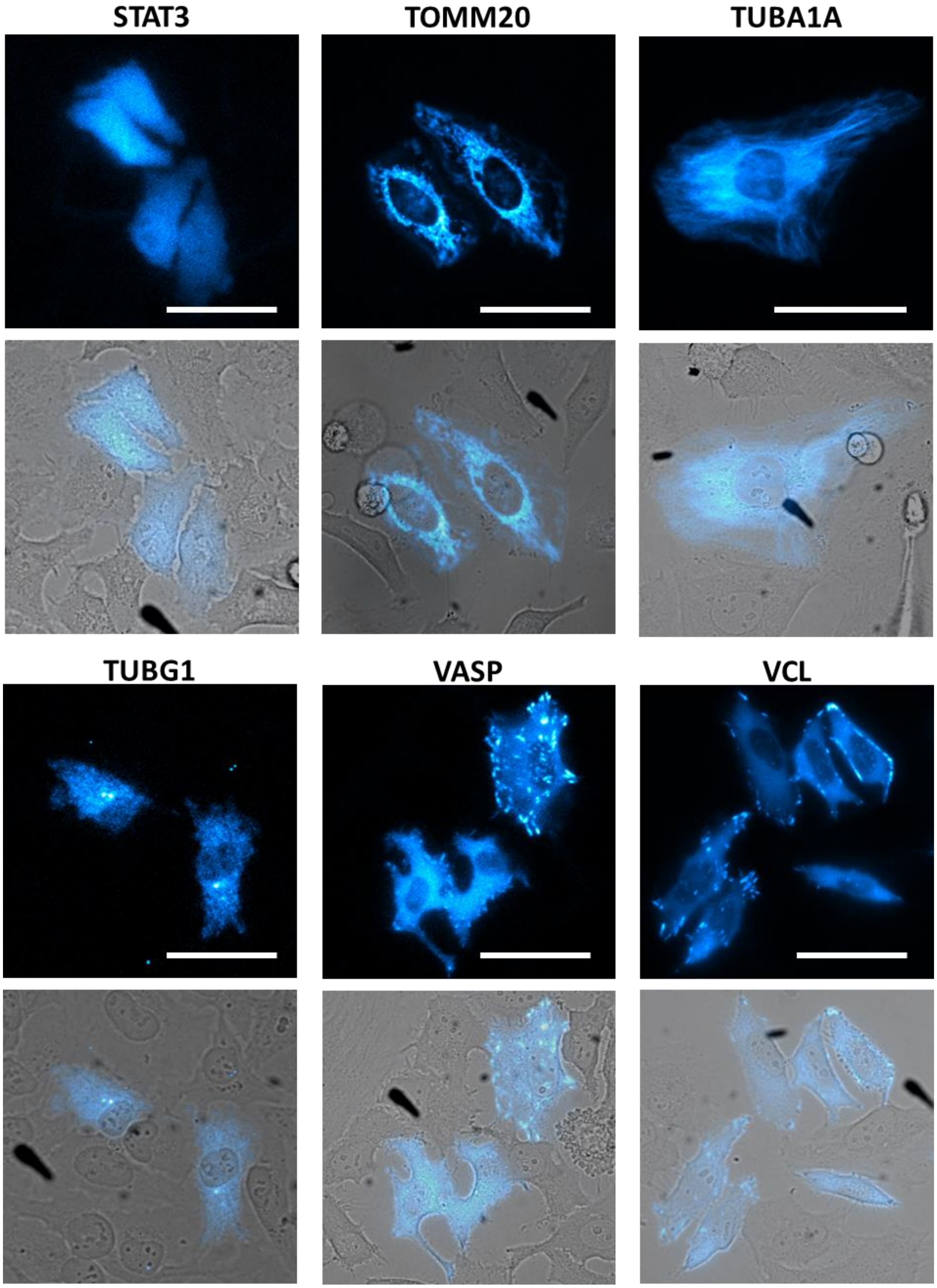

Supplementary Figure S7 (continued).

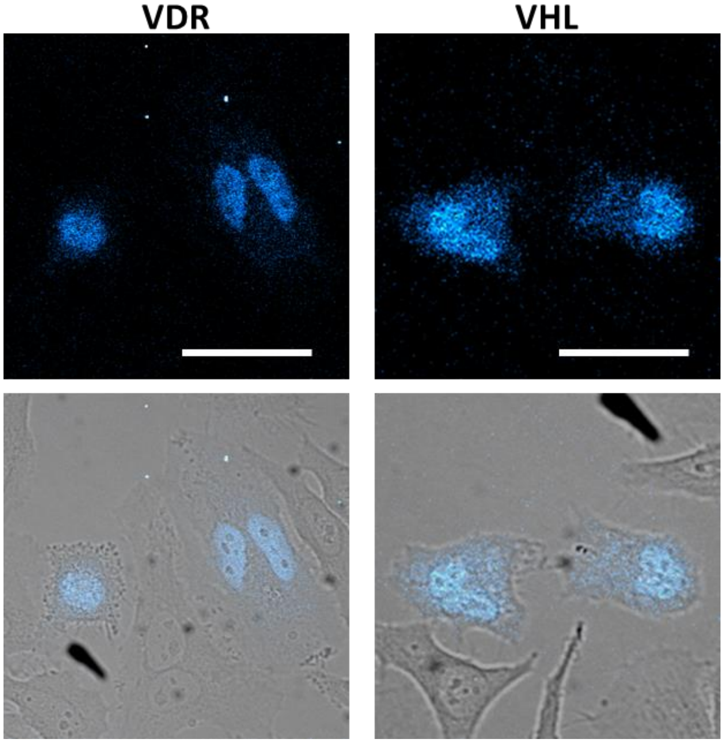

Supplementary Table S8. Instrument settings for bioluminescence imaging.

Target	Objective	Electron Multiplication	Exposure
ACTB	60x / NA 1.4 / Oil	300	2 s
AKT1	60x / NA 1.4 / Oil	1200	10 s
ARIH	60x / NA 1.4 / Oil	1200	15 s
ARNT	60x / NA 1.4 / Oil	1200	10 s
BAD	60x / NA 1.4 / Oil	1200	15 s
CASP3	60x / NA 1.4 / Oil	600	5 s
CAT	40x / NA 0.95 / Air	300	2 s
CAV1	60x / NA 1.4 / Oil	600	4 s
CENPA	60x / NA 1.4 / Oil	600	10 s
CFL1	60x / NA 1.4 / Oil	600	1 s
CREBBP	60x / NA 1.4 / Oil	600	10 s
CTNNB1	60x / NA 1.4 / Oil	1200	10 s
CUL1	40x / NA 0.95 / Air	600	3 s
EEA1	60x / NA 1.4 / Oil	600	4 s
EZH2	60x / NA 1.4 / Oil	1200	10 s
EZR	60x / NA 1.4 / Oil	600	5 s
GJA1	60x / NA 1.4 / Oil	600	2 s
GSK3B	60x / NA 1.4 / Oil	600	5 s
H3F3A	40x / NA 0.95 / Air	300	0.2 s
HDAC2	60x / NA 1.4 / Oil	300	1 s
HDAC6	60x / NA 1.4 / Oil	600	5 s
ITGB1	40x / NA 0.95 / Air	600	2 s
JUN	60x / NA 1.4 / Oil	600	3 s
LAMP1	60x / NA 1.4 / Oil	600	1 s
KPNB1	60x / NA 1.4 / Oil	300	2 s
MAPK8	40x / NA 0.95 / Air	1200	10 s
NFKBIA	40x / NA 0.95/ Air	1200	10 s
NIFK	60x / NA 1.4 / Oil	600	1 s
NOTCH1	100x / NA 1.4 / Oil	1200	30 s
NUMB	60x / NA 1.4 / Oil	600	5 s
NUP98	60x / NA 1.4 / Oil	600	5 s
PARP1	40x / NA 0.95 / Air	300	2 s
PEX5	60x / NA 1.4 / Oil	600	10 s
PRKAB1	60x / NA 1.4 / Oil	600	10 s
PTK2	60x / NA 1.4 / Oil	1200	10 s
PTPN11	60x / NA 1.4 / Oil	1200	10 s
RAC1	60x / NA 1.4 / Oil	1200	10 s
RB1	60x / NA 1.4 / Oil	1200	5 s
RHOA	40x / NA 0.95 / Air	300	2 s
RIPK1	40x / NA 0.95 / Air	1200	10 s
SIRT2	60x / NA 1.4 / Oil	1200	10 s
SMAD2	60x / NA 1.4 / Oil	1200	10 s
STAT3	60x / NA 1.4 / Oil	1200	10 s
TOMM20	60x / NA 1.4 / Oil	600	1 s
TUBA1A	60x / NA 1.4 / Oil	300	1 s
TUBG1	60x / NA 1.4 / Oil	1200	10 s
VASP	60x / NA 1.4 / Oil	600	5 s
VCL	60x / NA 1.4 / Oil	300	5 s
VDR	60x / NA 1.4 / Oil	1200	5 s
VHL	60x / NA 1.4 / Oil	1200	10 s

Supplementary Figure S8. Signal stability testing of Jurkat HiBiT pools for 69 targets over a period of 60 days (15 passages). HiBiT Luminescence was measured in lytic format at each passage. Data are represented as percent of luminescence signal relative to day 11 (100%). Green represents targets that maintained at least 33% of the original signal at passage 10, and gray represents those with at least 10% of the starting signal. Red represents targets that maintained less than 10% of signal by passage 10.

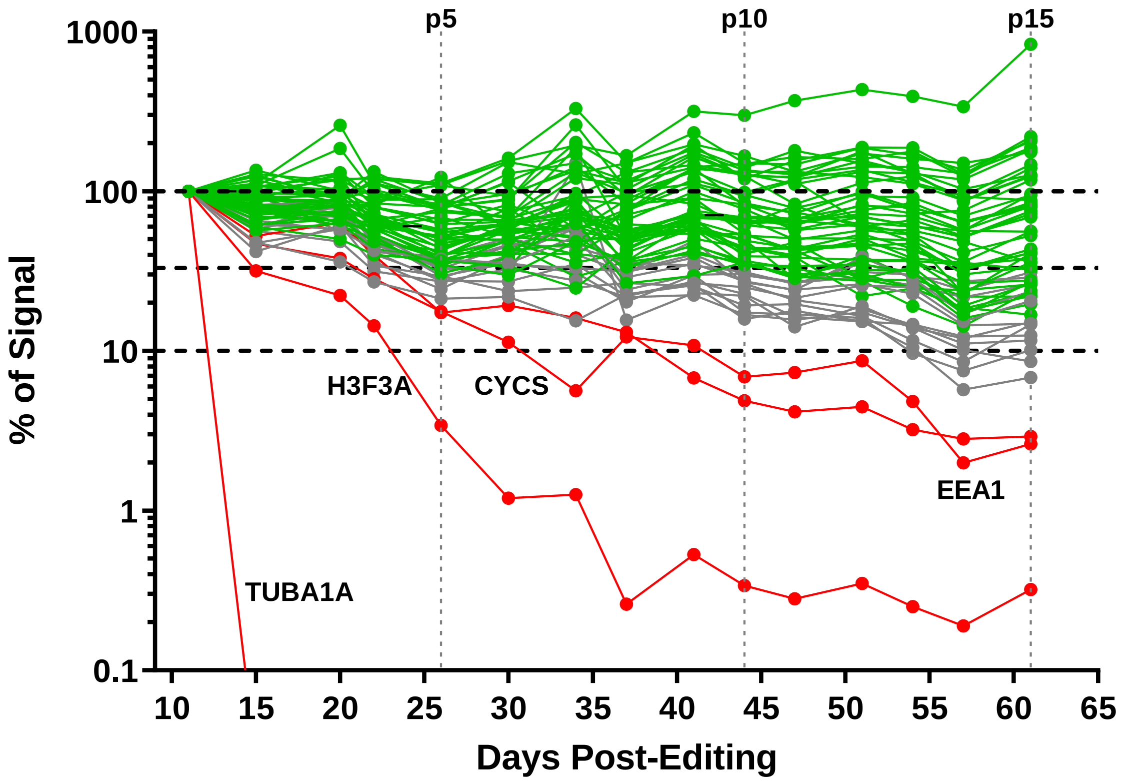

Supplementary Figure S9. Indel distribution in edited K-562 pools. Next-generation sequencing was performed on amplicons spanning the cut site for the (a) AKT1, (b) FOS, (c) IRAK4, and (d) MAPK8 targets. Bars indicate the number of reads for each indel size. Blue bar denotes an indel size of 0 bp, while red represents indel size of 39 bp (HiBiT). Indel sizes with read counts above 100 (dashed line) were used to calculate percent of wild type, HiBiT, or indel in the pools (see Supplementary Table S9).

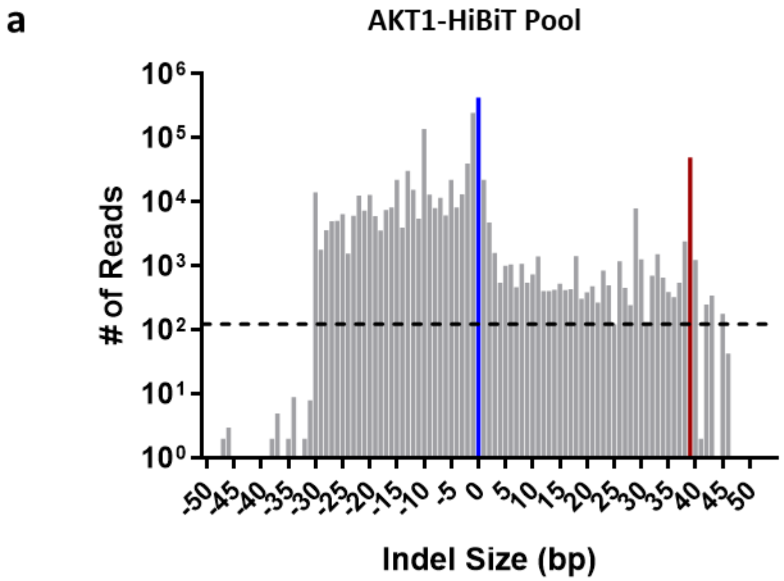

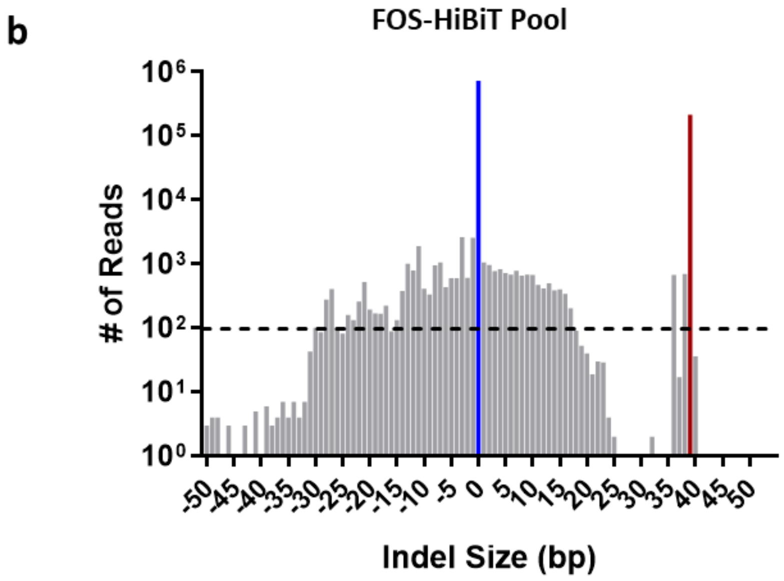

Supplementary Figure S9 (continued).

c

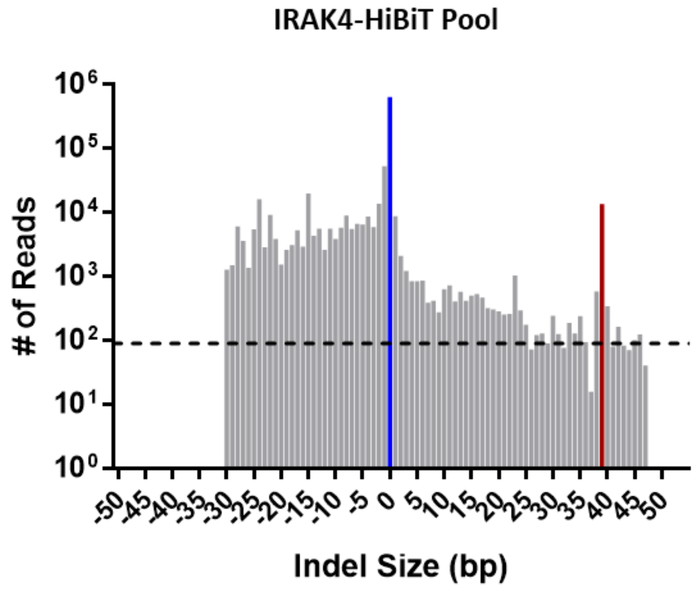

d

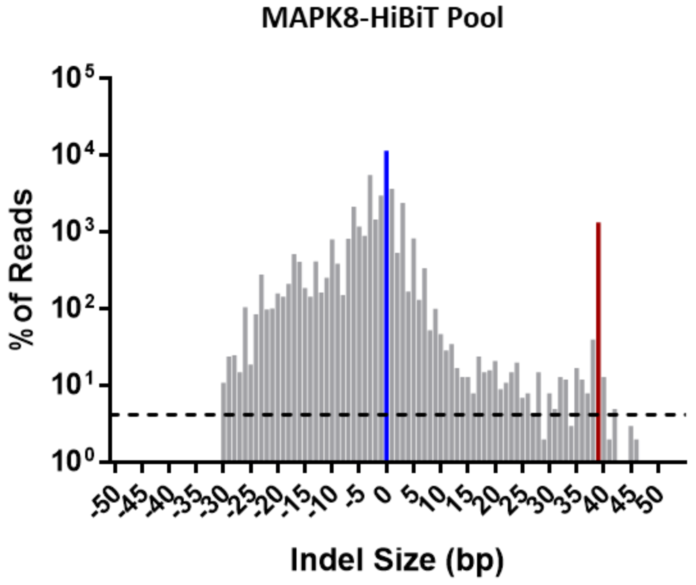

Supplementary Table S9. Next-generation sequencing of edited K-562 pools.

Target	Total Reads	% Wild Type	% HiBiT	% Indel
AKT1	1230686	35	4.0	61
FOS	970722	75	22	3.0
IRAK4	639026	71	2.0	27
MAPK8	41618	28	3	69

Supplementary Figure S10. Comparison of luminescence between edited pools and clones. Data represent single luminescence read relative to cell number obtained from either pools (white bars) or clones (blue bars) of the indicated target in (a) HeLa, (b) K-562, and (c) Jurkat cells. Dashed line denotes luminescence of unedited cells.

a

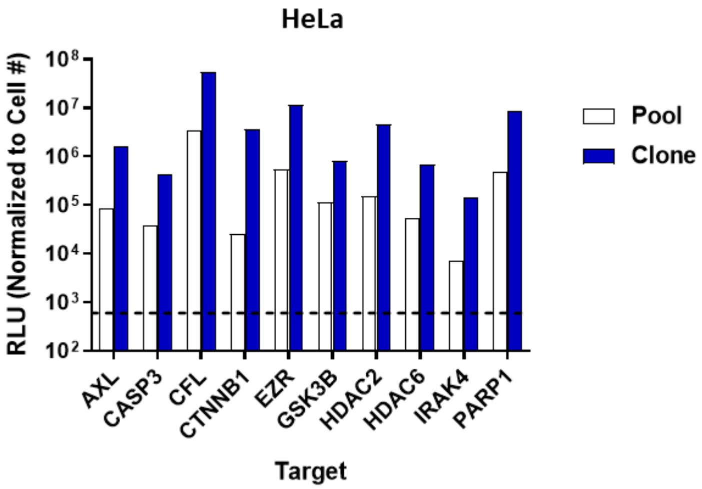

b

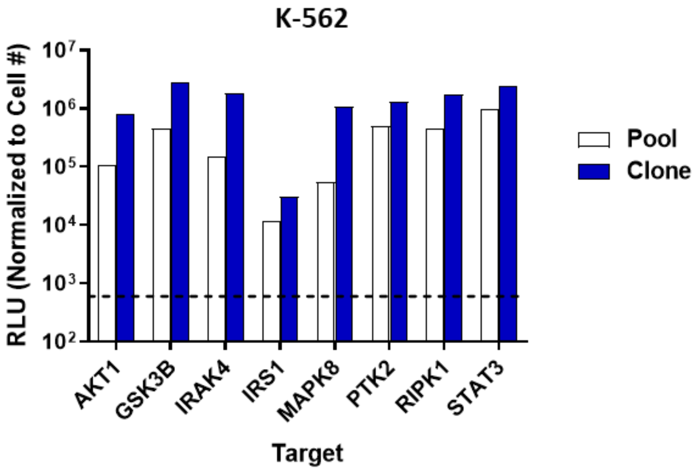

c

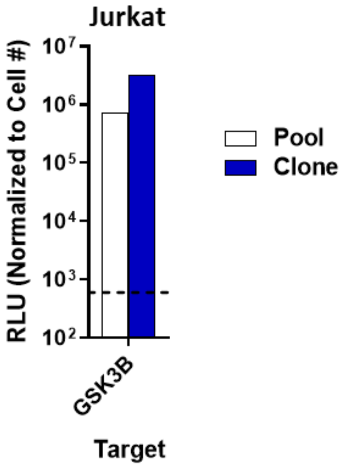

Supplementary Figure S11. HiBiT signal stability in clones. (a) HeLa, (b) K-562, and (c) Jurkat clones were maintained in culture for the indicated time period. At each passage, luminescence was measured in lytic format. Data represent individual luminescence reads normalized to cell number for the specified target.

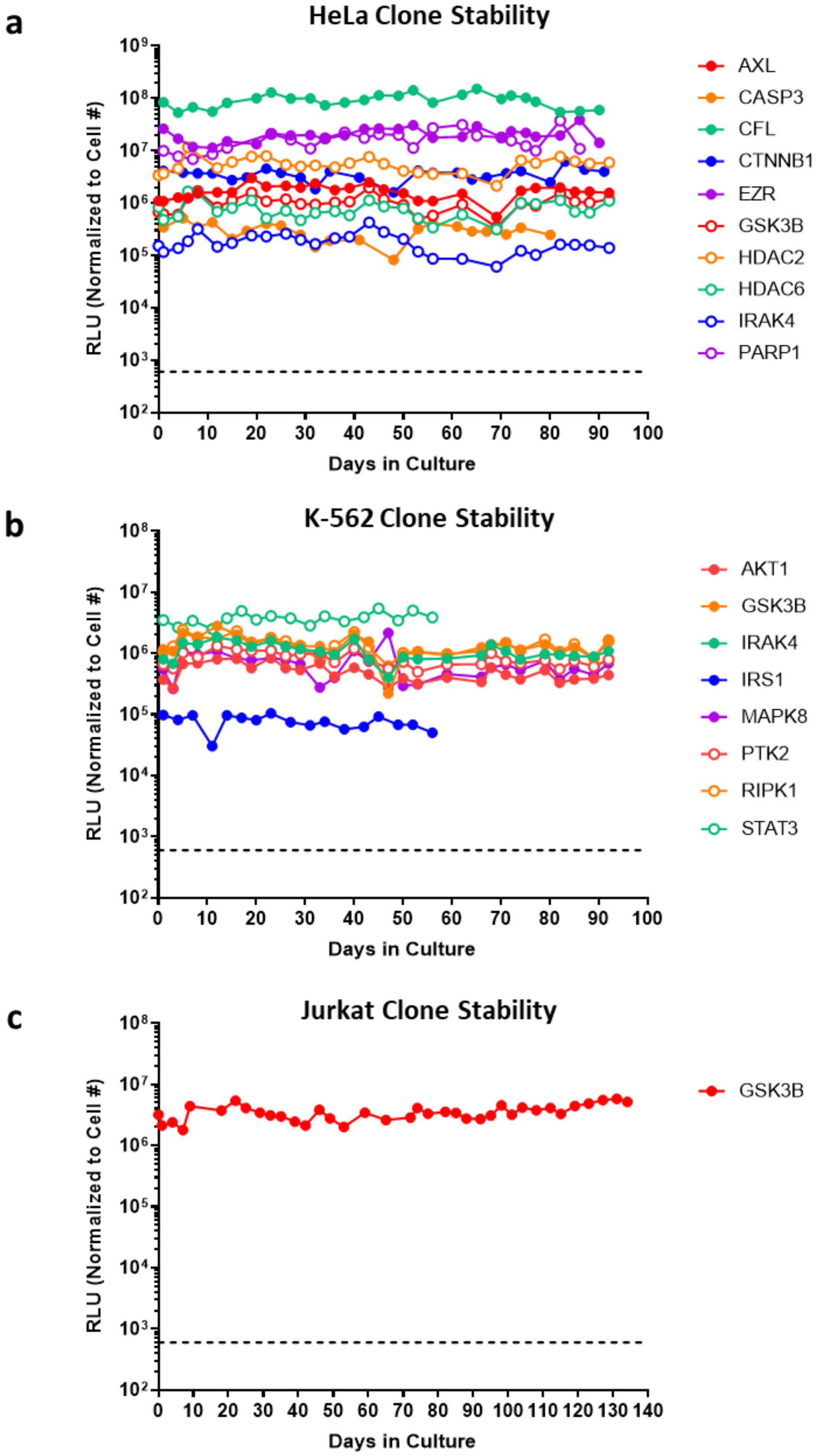

Supplementary Table S10. Sanger sequencing of clones.

Target	Cell Line	# of Sequencing Reads						
		Total	No HiBiT			HiBiT Integration		
		--	Intact CDS	Mutated CDS	Mutated 3'UTR	Intact CDS	Mutated CDS	Mutated 3' UTR
AKT1Clone 1	K-562	12	7	0	0	5	0	0
AKT1 Clone 2	K-562	22	12	0	12	10	0	10
AKT1 Clone 3	K-562	22	13	0	13	9	0	1
AKT1 Clone 4	K-562	19	10	0	0	9	0	9
AKT1 Clone 5	K-562	18	10	0	10	8	0	8
AKT1 Clone 6	K-562	16	0	10	10	6	0	0
AKT1 Clone 7	K-562	23	0	0	0	23	0	0
AKT1 Clone 8	K-562	19	8	0	0	11	0	11
AKT1 Clone 9	K-562	21	10	0	0	11	0	11
AXL	HeLa	22	7	0	7	15	0	0
CASP3	HeLa	23	0	0	0	23	0	0
CFL1	HeLa	23	17	2	3	4	0	0
CTNNB1	HeLa	21	5	0	0	16	0	0
EZR	HeLa	16	0	0	0	16	0	0
FOS	K-562	23	0	0	0	23	0	0
GSK3B	HeLa	17	10	0	0	7	0	0
GSK3B	K-562	24	0	0	0	24	0	0
GSK3B	Jurkat	23	0	15	0	8	0	0
HDAC2	HeLa	22	0	11	0	11	0	0
HDAC6	HeLa	22	0	0	0	22	0	0
IRAK4	HeLa	23	20	0	0	3	0	0
IRAK4	K-562	19	3	0	0	16	0	0
IRS1	K-562	24	0	0	0	24	0	0
MAPK8	K-562	13	5	0	0	8	0	0
NFKBIA	HeLa	14	0	9	0	5	0	0
PTK2	K-562	24	0	2	0	22	0	0
RIPK1	HeLa	15	0	14	0	1	0	0
RIPK1	K-562	23	0	0	0	23	0	0
STAT3	K-562	16	0	0	0	16	0	1

Supplementary Table S11. Droplet digital PCR analysis of HiBiT clones.

Gene	Cell Line	Clone #	Total Alleles	HiBiT Alleles	HiBiT Frequency
AKT1	K-562	1	196 ± 32	103 ± 15	52%
		2	519 ± 65	519 ± 65	100%
		3	282 ± 20	148 ± 12	52%
		4	796 ± 36	389 ± 33	49%
		5	487 ± 36	237 ± 38	49%
		6	2624 ± 94	2621 ± 95	100%
		7	512 ± 36	272 ± 19	53%
		8	7631 ± 131	3737 ± 58	49%
		unedited	1247 ± 64	0 ± 0	0%
FOS	K562	1	3390 ± 280	3388 ± 279	100%
		2	1170 ± 239	601 ± 122	51%
		3	2742 ± 223	1361 ± 98	50%
		4	984 ± 80	496 ± 42	50%
		5	552 ± 104	254 ± 24	46%
		6	322 ± 43	158 ± 10	49%
		7	591 ± 65	287 ± 41	49%
		8	5484 ± 1107	2725 ± 583	50%
		unedited	1138 ± 96	0 ± 0	0%
IRAK4	K562	1	937 ± 134	301 ± 38	32%
		2	928 ± 250	313 ± 86	34%
		3	278 ± 100	93 ± 35	33%
		4	519 ± 47	353 ± 13	68%
		5	1425 ± 148	946 ± 94	66%
		6	580 ± 66	187 ± 31	32%
		7	41 ± 3	28 ± 6	69%
		unedited	1241 ± 134	0 ± 0	0%
GSK3B	K-562	1	4909	3252	66%
		unedited	3915	0	0%
RIPK1	K-562	1	9948	9938	100%
		unedited	20410	0	0%
STAT3	K-562	1	1305	1305	100%
		unedited	2668	0	0%
CASP3	HeLa	1	4502	4494	100%
		unedited	989	0	0%
HDAC2	HeLa	1	4912	2370	48%
		unedited	5570	0	0%
IRAK4	HeLa	1	4578	1083	24%
		unedited	9017	0	0%
NFKBIA	HeLa	1	1518	1002	66%
		unedited	4673	0	0%
RIPK1	HeLa	1	5321	1642	31%
		unedited	1712	0	0%

Supplementary Figure S12. 2D Droplet-digital PCR plots of 11 clones (a-k). Fluorescence amplitude plots show droplets containing the target gene (blue) and droplets containing the HiBiT insertion and target insertion (orange). Negative ddPCR droplets are in gray. Total alleles, HiBiT alleles, and HiBiT frequency are summarized in Supplementary Table S11.

a

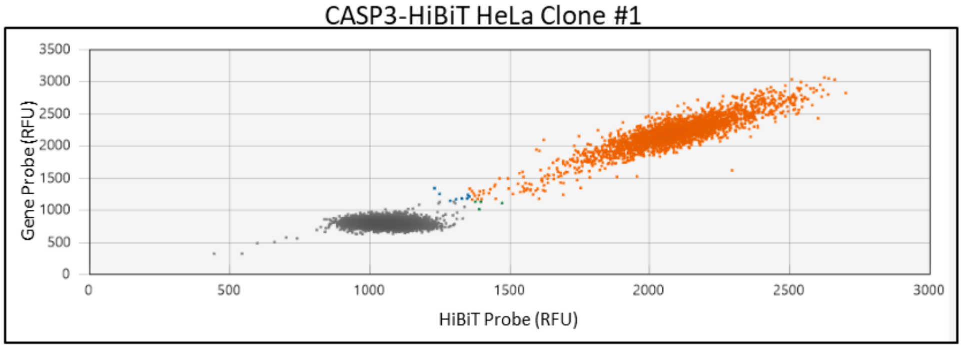

b

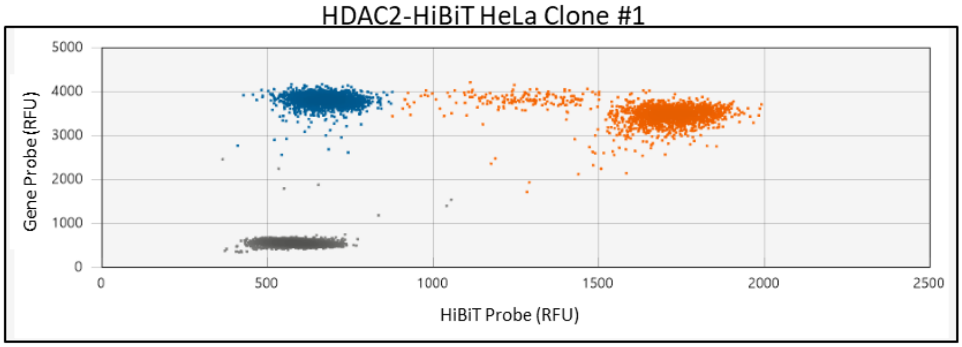

c

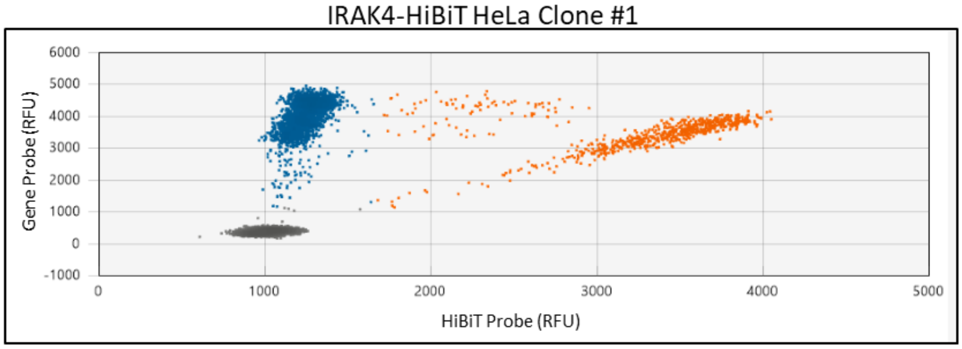

d

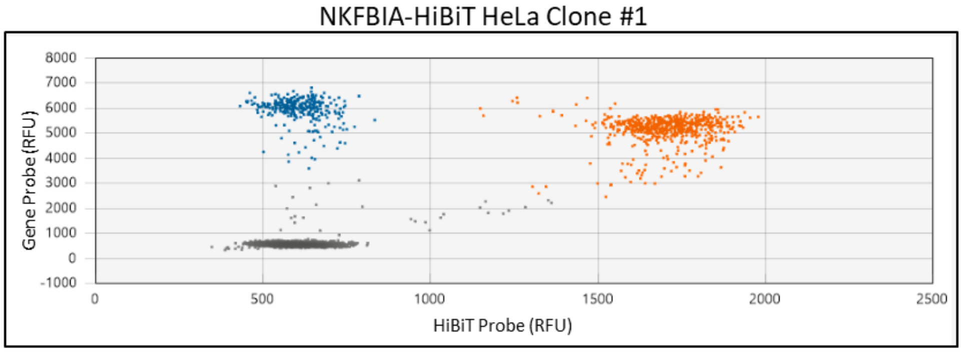

Supplementary Figure S12 (continued).

e

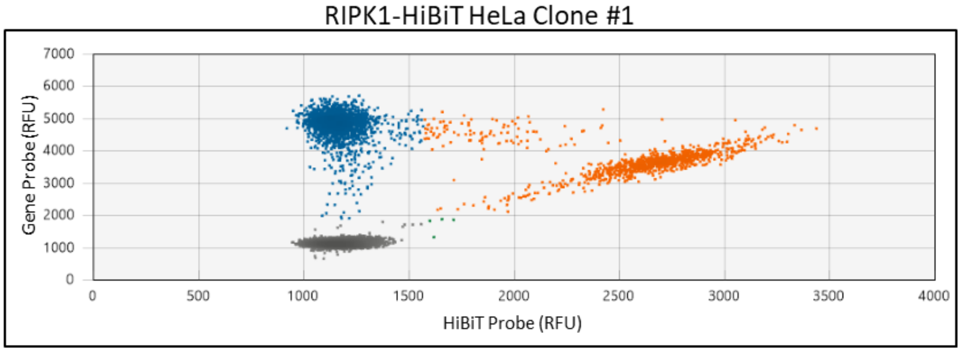

f

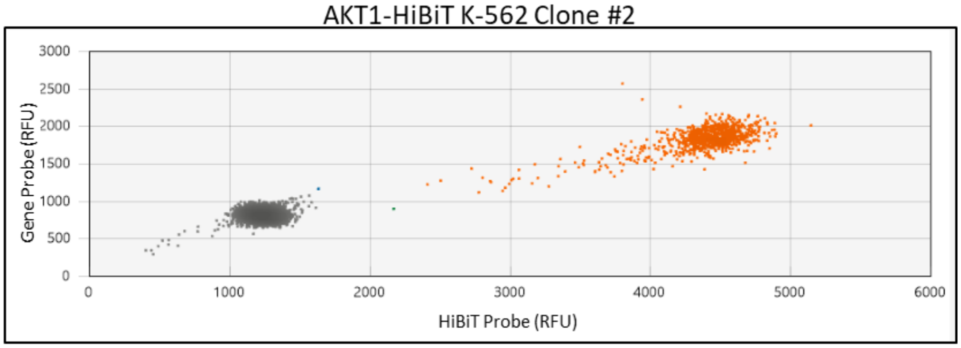

g

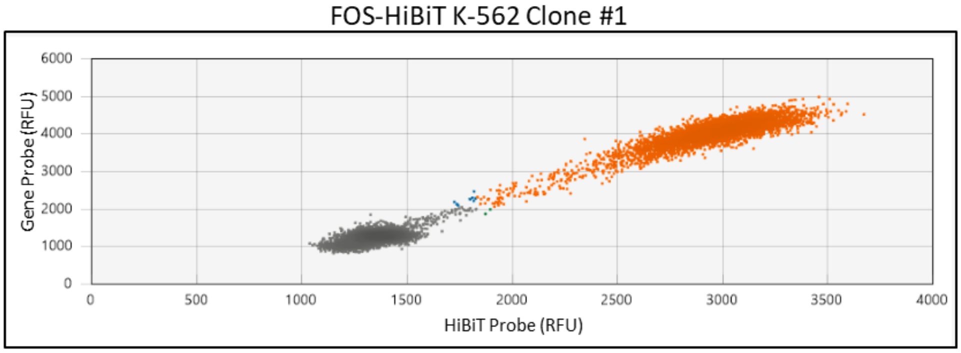

h

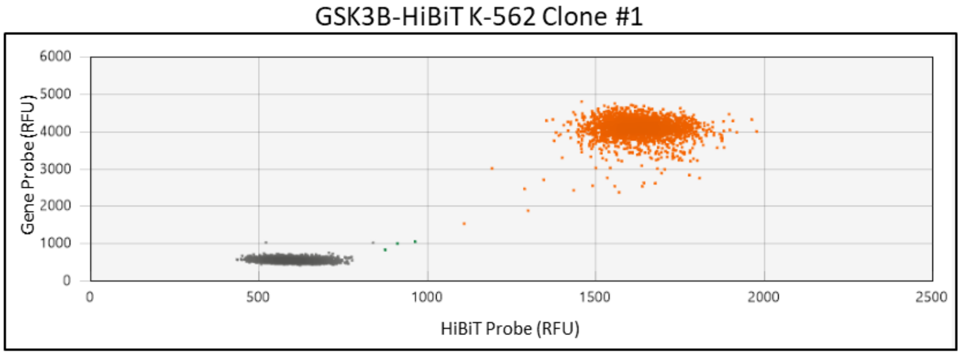

Supplementary Figure S12 (continued).

i

IRAK4-HiBiT K-562 Clone #1

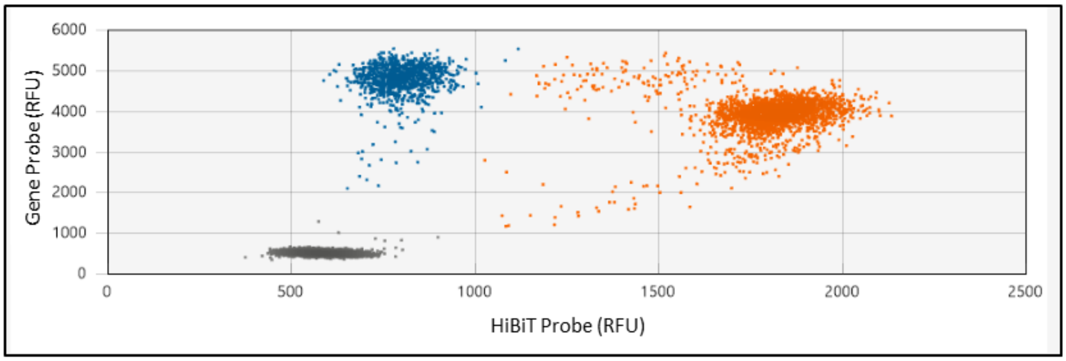

j

RIPK1-HiBiT K-562 Clone #1

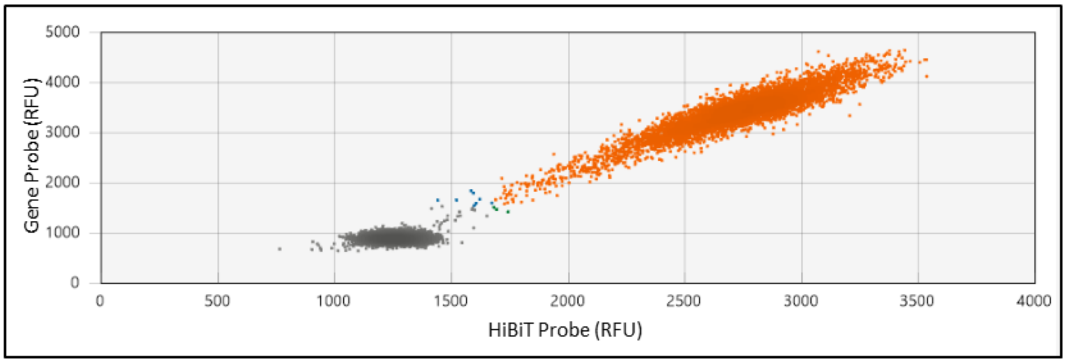

k

STAT3-HiBiT K-562 Clone #1

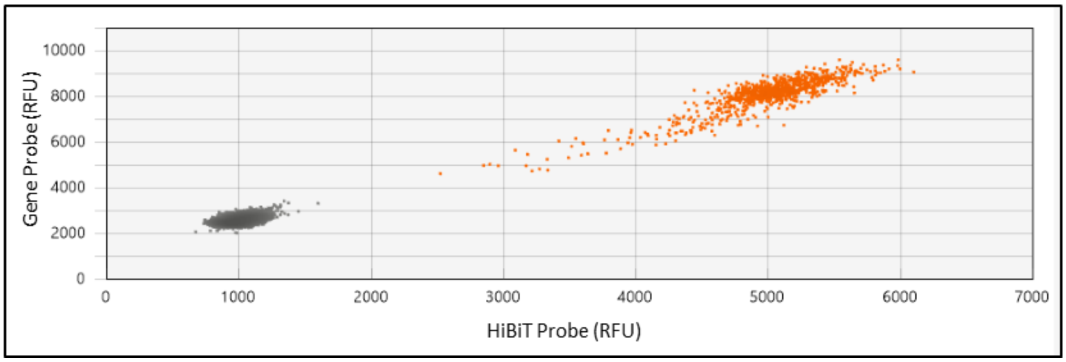

Supplementary Figure S13. Full-size unedited Western blots shown in in Fig. 5b. (a) Blot for c-FOS from cells treated with increasing concentrations of PMA. (b) Blot for COX IV loading control. (c) Brightfield image of plot showing molecular weight ladder (kb). Red boxes denote where blots were cropped to generate Fig. 5b.

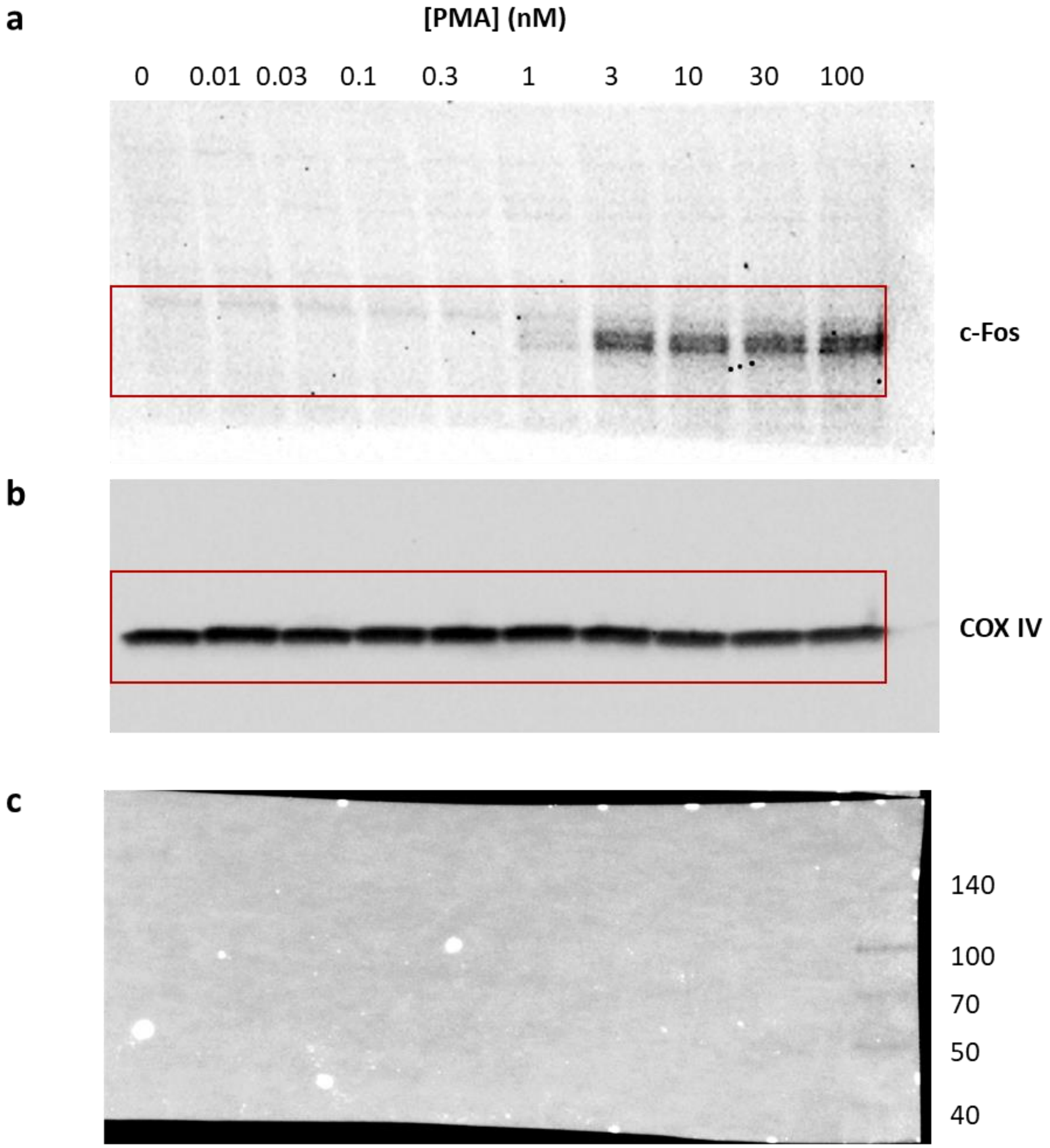

Supplementary Figure S14. Full-size unedited Western blots shown in Fig. 6b and Fig. 6c. (a) Blot for IκBα from cells treated with increasing concentrations of TNFα (left side) or 20 ng/ml TNFα for various times (ride side). (b) Blot for COX IV loading control. Red boxes denote where blots were cropped to generate Fig. 6b and Fig. 6c.

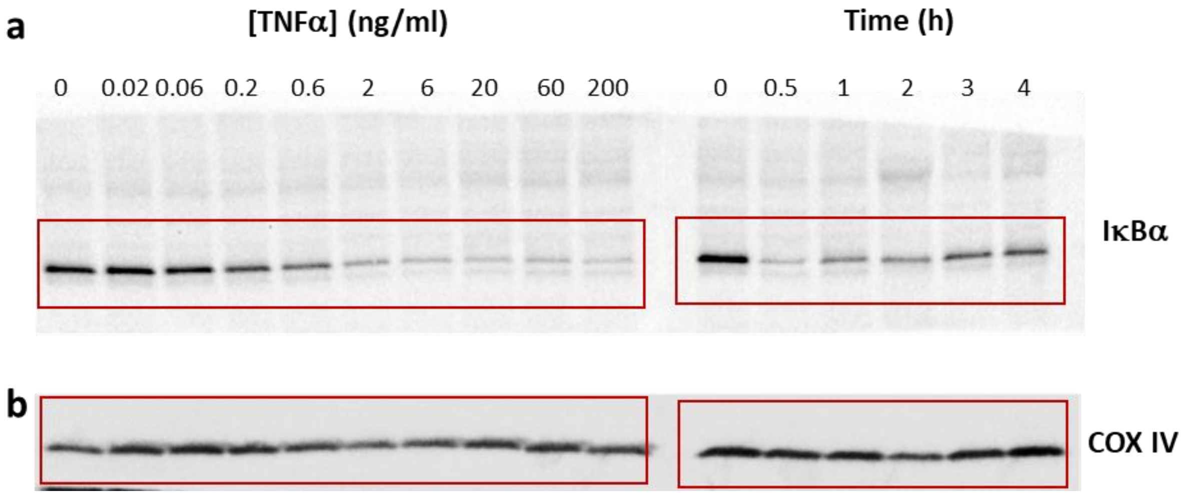

Supplementary Table S12. Amplicon sequencing primers.

Target	Forward Primer	Reverse Primer
AKT1	GTATTAATACGACTCACTATAGGGCTAGCGG AGGTTGGCTTCCTACTGGAG	CGGATCAGCTTGCATGCCTGCAGGTCGACTG CCCACAGCACAAAAACGTC
AXL	GTATTAATACGACTCACTATAGGGCTAGCGC AACCCAGCCAGACCCTAAGG	CGGATCAGCTTGCATGCCTGCAGGTCGACTT CCAACCCCTTCCTTTTACAGATG
CASP3	GTATTAATACGACTCACTATAGGGCTAGCGA GTCGCTTTGTGCCATGC	CGGATCAGCTTGCATGCCTGCAGGTCGACTT GCCTCTCATAATGACTGCACC
CFL	GTATTAATACGACTCACTATAGGGCTAGCGG CTTTGATTGGCTCCTTCCC	CGGATCAGCTTGCATGCCTGCAGGTCGACTC AAGGGATGGAGGGAGAAGG
CTNNB1	GTATTAATACGACTCACTATAGGGCTAGCGG GGCTGCCTCCAGGTGACAG	CGGATCAGCTTGCATGCCTGCAGGTCGACTA CTTCCACACATGATCACATAACAACC
EZR	GTATTAATACGACTCACTATAGGGCTAGCGA GGGACTAGACCAATGCTGC	CGGATCAGCTTGCATGCCTGCAGGTCGACTT GGTTCCCAGCCCAGAATG
FOS	GTATTAATACGACTCACTATAGGGCTAGCGT ACTCCCAGCTGCACTGC	CGGATCAGCTTGCATGCCTGCAGGTCGACTA CGCACAGATAAGGTCCTCC
GSK3B	GTATTAATACGACTCACTATAGGGCTAGCGG GACTTTTTGACTTGCAGATGCTAATAC	CGGATCAGCTTGCATGCCTGCAGGTCGACTG AGAGATTGTATGTTCTAGTGCTCCGC
HDAC2	GTATTAATACGACTCACTATAGGGCTAGCGC CAGCCTTTACAGGATAACTTGGAATAG	CGGATCAGCTTGCATGCCTGCAGGTCGACTG CCAAAGTAGTATAAAATGAAGCCAGAAG
HDAC6	GTATTAATACGACTCACTATAGGGCTAGCGG GGATAGTCCAGAAGACAGAGTG	CGGATCAGCTTGCATGCCTGCAGGTCGACTT CCACAATTAATCGTCGCAGTTC
IRAK4	GTATTAATACGACTCACTATAGGGCTAGCGT TAAAAAGTGAAAGGGGTGGGG	CGGATCAGCTTGCATGCCTGCAGGTCGACTT GCTTGAAGGAGATTTCTCAGGG
IRS1	GTATTAATACGACTCACTATAGGGCTAGCGC TGGGGGTTTGGAGAATGGTC	CGGATCAGCTTGCATGCCTGCAGGTCGACTG GAAGGGGCAGAGGCGAAG
MAPK8	GTATTAATACGACTCACTATAGGGCTAGCGC CTCATAGCTGTCAATGAAGAGTG	CGGATCAGCTTGCATGCCTGCAGGTCGACTC GACTCCCCATCCCTCC
PTK2	GTATTAATACGACTCACTATAGGGCTAGCGG CTGACTGCTGCTCACGCC	CGGATCAGCTTGCATGCCTGCAGGTCGACTG GTTAATTCCTCGCTGCTGGTG
RIPK1	GTATTAATACGACTCACTATAGGGCTAGCGT GGGCTTCACACAGTCTCAGATTG	CGGATCAGCTTGCATGCCTGCAGGTCGACTG CAGACACAGAACCCCTATCAGTG
STAT3	GTATTAATACGACTCACTATAGGGCTAGCGC CTTAAGGGGCAGGAGATTGG	CGGATCAGCTTGCATGCCTGCAGGTCGACTG CAGATCACCCACATTCACTC

Supplementary Table S13. Droplet digital PCR primer and probes.

Target	Forward Primer	Reverse Primer	Gene Probe	HiBiT Probe
AKT1	CTGTCTACACCC ACAGATGAC	CTCAAATGCACC CGAGAAATAAA	/56-FAM/ATGGAGTGT/ZEN/GTGGACAGCGAGC/3IABkFQ/	/5-HEX/TAATCTTCT/ZEN/TGAACAGCCGCCAGCC/3IABkFQ/
CASP3	TTACCCGGGTTA ACCGAAAG	CCTGACTGGAAG TTTGAGGTAG	/56-FAM/TTTGACCTA/ZEN/CTCTCATGCTGCAGAGG/3IABkFQ/	/5-HEX/TAATCTTCT/ZEN/TGAACAGCCGCCAGCC/3IABkFQ/
FOS	GTCTTCCTTCGTC TTCACCTAC	CTCCTCTCTGTA ATGCACCAG	/56-FAM/AGTCAGAGG/ZEN/AAGGCTCATTGCTGC/3IABkFQ/	/5-HEX/TAATCTTCT/ZEN/TGAACAGCCGCCAGCC/3IABkFQ/
GSK3B	TAGCATTGCTCA TGCTCTCTC	TCTTTCCAAACG TGACCAGT	/56-FAM/TGCTAATAC/ZEN/TGGAGACCGTGGACAGA/3IABkFQ/	/5-HEX/TAATCTTCT/ZEN/TGAACAGCCGCCAGCC/3IABkFQ/
HDAC2	GCATTTAAACCA AGATTGTGCCA	ACAGTCCATGCC AAAGTAGTATAA	/56-FAM/CCAAATCAG/ZEN/AACAGCTCAGCAACCC/3IABkFQ/	/5-HEX/TAATCTTCT/ZEN/TGAACAGCCGCCAGCC/3IABkFQ/
IRAK4	CCGCTCCGGAAG CTTAAC	CAGTGTGGATAT AAGTACACCCTT TA	/56-FAM/AGGTTCAAC/ZEN/AGCTGCTGCAAGAGA/3IABkFQ/	/5-HEX/TAATCTTCT/ZEN/TGAACAGCCGCCAGCC/3IABkFQ/
NFKBIA	CCGCTCCGGAAG CTTAAC	CAGTGTGGATAT AAGTACACCCTT TA	/56-FAM/TGATGACTG/ZEN/TGTGTTTGGAGGCCA/3IABkFQ/	/5-HEX/TAATCTTCT/ZEN/TGAACAGCCGCCAGCC/3IABkFQ/
RIPK1	GGTGATGAGGG AAGGCATAAA	GAGTCCAGAAG TTTGAGACCAG	/56-FAM/TTGGCTGCC/ZEN/TCAGAGCATTCAGAA/3IABkFQ/	/5-HEX/TAATCTTCT/ZEN/TGAACAGCCGCCAGCC/3IABkFQ/
STAT3	AGAGGGTGGAC AACTGAACTA	GGGTTTGGCTGT GTGAGG	/5-HEX/TTTCCCTGT/ZEN/CTGTCCCTCCAGAGT/3IABkFQ/	/56-FAM/TAATCTTCT/ZEN/TGAACAGCCGCCAGCC/3IABkFQ/
